# Endurance exercise and selective breeding for longevity extend *Drosophila* healthspan by overlapping mechanisms

**DOI:** 10.18632/aging.100789

**Published:** 2015-08-08

**Authors:** Alyson Sujkowski, Brian Bazzell, Kylie Carpenter, Robert Arking, Robert J Wessells

**Affiliations:** ^1^ Department of Physiology, Wayne State School of Medicine, Detroit, MI 48201, USA; ^2^ Department of Biological Science, Wayne State University, Detroit, MI 48201, USA

**Keywords:** endurance training, selective breeding, healthspan, cardiac performance, mobility

## Abstract

Endurance exercise has emerged as a powerful intervention that promotes healthy aging by maintaining the functional capacity of critical organ systems. In addition, long-term exercise reduces the incidence of age-related diseases in humans and in model organisms. Despite these evident benefits, the genetic pathways required for exercise interventions to achieve these effects are still relatively poorly understood. Here, we compare gene expression changes during endurance training in *Drosophila melanogaster* to gene expression changes during selective breeding for longevity. Microarrays indicate that 65% of gene expression changes found in flies selectively bred for longevity are also found in flies subjected to three weeks of exercise training. We find that both selective breeding and endurance training increase endurance, cardiac performance, running speed, flying height, and levels of autophagy in adipose tissue. Both interventions generally upregulate stress defense, folate metabolism, and lipase activity, while downregulating carbohydrate metabolism and odorant receptor expression. Several members of the *methuselah-like (mthl)* gene family are downregulated by both interventions. Knockdown of *mthl-3* was sufficient to provide extension of negative geotaxis behavior, endurance and cardiac stress resistance. These results provide support for endurance exercise as a broadly acting anti-aging intervention and confirm that exercise training acts in part by targeting longevity assurance pathways.

## INTRODUCTION

Modern, sedentary lifestyles and highly nutritive diets have contributed to an increase in the incidence of obesity, cardiovascular disease, and diabetes [1]–[3]. These problems are particularly evident in the aging population, where these and other age-related diseases reduce independence and quality of life for the elderly [4], [5]. Endurance exercise has emerged as a powerful intervention to promote healthy aging [6]–[8]. Exercise training has been found to extend the healthy function of multiple organ systems, including heart [9], skeletal muscle [10], and brain [11], [12]. These functional effects are associated with substantial metabolic remodeling in humans [13]–[15] and in vertebrate models [16]–[18]. Less well understood are the changes to gene expression that are necessary for this remodeling to occur.

Although several important single genes and pathways have been identified using vertebrate models [19]–[22], the lack of an endurance exercise paradigm for an invertebrate species has impaired the use of large-scale genetic screens for exercise-induced factors. We have developed an endurance training paradigm for *Drosophila*, using a machine known as the Power Tower [23], that uses reiterated induction of the negative geotaxis instinct to allow controlled, daily training of fruit fly cohorts [23].

Following a three-week period of daily, ramped endurance exercise activity, wild-type flies display increased climbing speed [24], endurance [25], cardiac performance [24], [26], and mitochondrial enzyme activity [24]. Furthermore, endurance training has been demonstrated to increase mitochondrial number and reduce accumulated oxidative stress in fly cardiac muscle [27]. Exercise also induces consumption of accumulated fat stores in animals with excess lipid [26]. Taken together, these observations support the idea that endurance training in *Drosophila* induces similar effects as training in humans [28]–[30] and vertebrate models [15, 31–33].

These observations raise two important questions about this model: 1) what are the genes and pathways induced by exercise in the *Drosophila* model? 2) Which of these genes are associated with increased healthspan of the animal? One way to answer these questions would be to concurrently, but independently, induce longevity extension and exercise training in genetically isogenous fly cohorts, then compare the overlap in gene expression changes between the two interventions. The intersection of gene expression changes induced by both endurance training and longevity extension should be enriched for genes that act as intermediates to assure healthspan improve-ments in exercise trained animals.

To do this, we took advantage of a fly line generated by selective breeding for longevity, known as the *La* line [34]. This line exhibits extended longevity, particularly in the early, healthy portion of the survival curve [35]. This profile is characteristic of an intervention that genuinely extends healthspan, rather than simply extending the time between senescence and death. Longevity extension in *La* flies acts through mechanisms that are partially overlapping with those induced by dietary restriction [36] and curcumin feeding [37], and are also dependent, in part, on changes to mitochondrial efficiency [36], [38]. Dietary restriction, in turn, induces changes to wild type exercise capacity that are similar to those seen following exercise training [39].

We have previously shown that the *La* line climbs higher than wild-type in a rapid negative geotaxis assay [24], while its parental strain, known as *Ra*, behaves normally in negative geotaxis assays. Importantly, the *Ra* strain exhibits a robust exercise response, improving in speed following a three-week training program, while the *La* line shows much smaller improvement when trained [24], suggesting that *La* flies may already be receiving some of the benefits of exercise training through their selective breeding.

Here, we measure physiological changes to endurance and cardiac function induced by exercise training to *Ra* or *La* flies, then compare changes in gene expression induced in *Ra* flies by exercise to differences induced in *Ra* flies by selective breeding.

## RESULTS

### Lifespan/mortality

**Figure 1:**
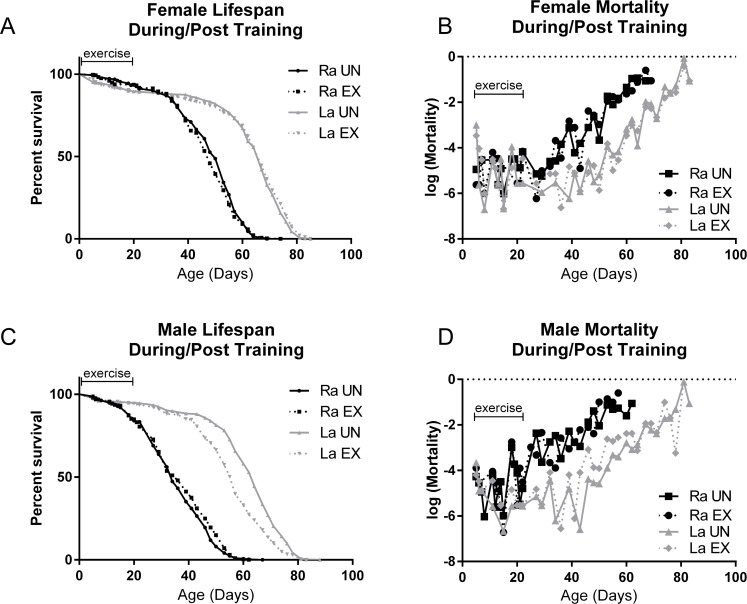
Selectively bred *La* flies increase longevity whether exercised or not (**A**) Female *La* flies live longer than the control *Ra* females after three weeks of exposure to the Power Tower training machine (logrank; p <.0001). Training has little effect on longevity of either line. (**B**) *La* females have a delayed increase in mortality with age that is independent of exercise. Mortality during the training period is similar for all groups. (**C**) Male *La* flies live longer than control *Ra* males after three weeks of exposure to the training machine (logrank; p <.0001). Training does not increase longevity, and slightly decreases longevity in *La* males. (**D**) Mortality of *La* males is similar to *Ra* males during training, but is consistently lower than control *Ra* males after day 20. Exercised *La* males had somewhat increased mortality between days 40 and 65 compared to unexercised *La* males. *n* ≥ 250 for all survival and mortality experiments. Mortality and survival data are from same cohorts.

*La* flies are a product of selective breeding for longevity and are longer-lived than the parental *Ra* lines on standard laboratory diets [40]–[42]. We subjected *La* and *Ra* flies to three weeks of endurance exercise, using a ramped daily protocol [24]. Survival and age-specific mortality were measured during training and continued to be measured for the duration of the lifespan following the training period. *Ra* longevity was not significantly affected by three weeks of exercise training, when compared to control siblings that were placed on the machine for the same time, but restrained from exercising by a foam stopper pushed down into the vial. *La* longevity was not further extended by three weeks of exercise training, and *La* flies lived longer than *Ra* whether exercised or not (Figure 1A, B). In male flies only, *La* flies trended toward a slightly lower longevity following training, although this effect is small in comparison to the longevity difference between *La* and *Ra* males (Figure 1C).

It is possible that the limitation of exercise to a three-week period may have limited the impact of training on longevity, as compared to lifelong exercise. However, in order to best visualize exercise-induced changes to gene expression, independent of changes induced late in life by aging, we stopped exercise training at three weeks for all experiments in this study and performed all assessments on age-matched animals immediately following training.

Because we often observe age-independent deaths due to damage during the training of any fly line, we wondered whether a selection effect for the fittest flies might contribute to the improved physiology of trained flies. To address this question, we measured age-specific mortality during the training period. If exercise acts by selecting flies that are resistant to training stress, then exercised *Ra* flies should have higher mortality during training than unexercised *Ra* flies. Furthermore, the *La* flies, previously established as long-lived and stress-resistant, should have lower mortality during training than *Ra* flies.

Age-specific mortality measures reveal that *La* flies have a delayed induction of age-related mortality increase rather than a decrease in the slope of age-related mortality increase (Figure 1B, D), in agreement with previous observations [43]. Mortality slope of both lines during the training period was sporadic and showed no consistent difference between exercised and unexercised (Figure 1B, D).

### Negative geotaxis performance of exercised and long-lived Flies

**Figure 2:**
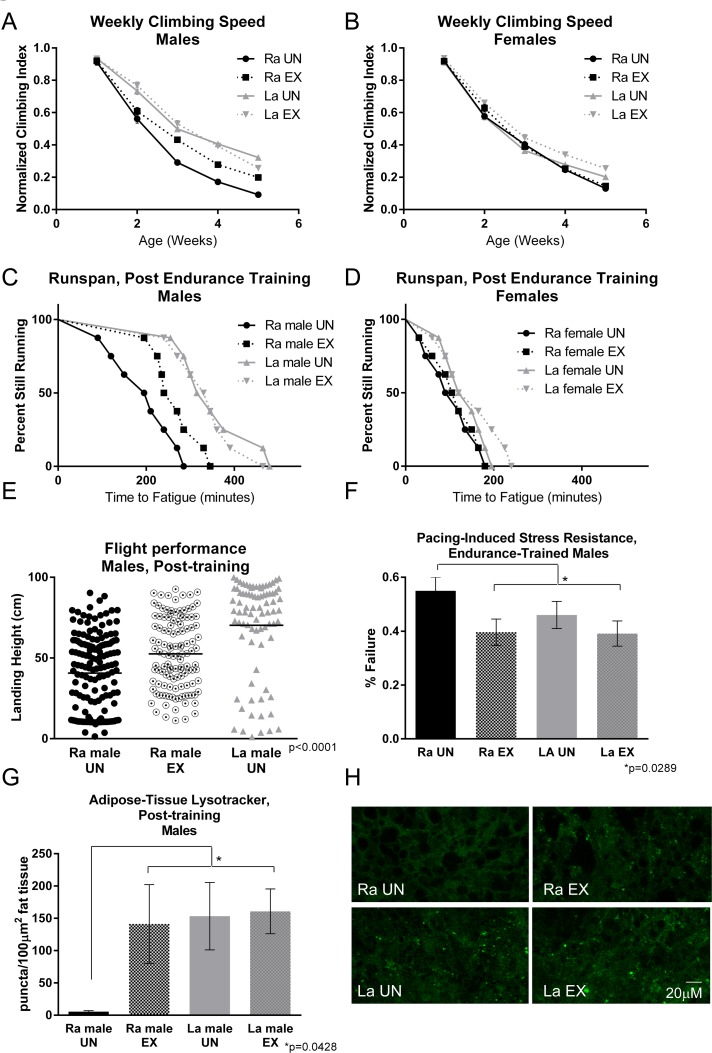
Exercise training increases performance of male control flies, but not long‐lived *La* flies (**A**) Exercised *Ra* males are protected against declining negative geotaxis speed with age compared to unexercised controls (two‐way ANOVA, p <.0001, *n* ≥ 100). *La* males have higher negative geotaxis speed than *Ra* males across ages and receive no further benefit from exercise. (**B**) Female control flies were unaffected by training in negative geotaxis speed across five weeks of age, while female *La* flies showed a slight trend toward increased speed. (**C**) *Ra* males improved endurance significantly following training (log‐rank; p <.0001, *n* ≥ 160). *La* males showed higher endurance than *Ra* (log‐rank; p <.0001), but received no further benefit from exercise. (**D**) Neither *Ra* nor *La* females receives a benefit to endurance following training, although *La* showed a trend toward an increase (log‐rank; p = .2290, *n* ≥ 160). (**E**) *Ra* males improved flight ability following training. *La* males display higher landing height than both untrained and trained *Ra* flies. (ANOVA, p <.0001, *n* ≥ 160). (**F**) Unexercised *La* males had significantly less cardiac failure in response to electrical pacing stress than did unexercised *Ra* (error bars indicate ±SD, t‐test; p = .0289, *n* ≥ 100). Following training, *Ra* males had statistically similar failure rates to exercised or unexercised *La*. (**G**) Unexercised *La* males showed significantly higher Lysotracker staining in dissected abdominal adipose tissue than unexercised *Ra* (error bars indicate ±SD, t‐test; p =.0428, *n* ≥ 10). Following training, *Ra* flies showed statistically similar Lysotracker staining to exercised or unexercised *La*.

*La* flies have a higher climbing index than *Ra* flies in an acute test of climbing speed measured longitudinally across five weeks (Figure 2A,B), as previously observed [24]. However, when exercise trained, male *Ra* flies improve their speed significantly (Figure 2A). Age-matched *La* males, by contrast, derive no further benefit from training. Female flies of either genotype do not significantly benefit from training (Figure 2B).

In order to examine endurance directly, we developed a fatigue tolerance assay [23], in which flies are placed on the exercise training device in vials of 20 and allowed to run to exhaustion. Vials are removed from the machine when less than five flies are still running and considered “fatigued”. Vial removal is analyzed as a time-to-event curve with each removal treated as an event, equivalent to a death in a survival curve. We refer to the resulting curve as a “runspan”.

Male *La* flies showed clear improvements in endurance compared to age-matched, three-week old *Ra* flies (Figure 2C). Exercise training caused a dramatic increase in the endurance of three-week old male *Ra* flies compared to unexercised sibs (Figure 2C), although they still did not equal the endurance of *La* males. *La* males do not gain further benefit from training. Three-week old female flies of either genotype showed less endurance than male sibs, and neither responded significantly to exercise training, although *La* females showed a slight trend toward improvement (Figure 2D).

### Flight performance

Untrained three-week old *La* flies have significantly higher flight index than age-matched, untrained *Ra* flies (Figure 2E). Three-week old male *Ra* flies improve their score on a flight index significantly following exercise training, indicating that running induces a systemic response that improves other functional assays as well (Figure 2E). This result is consistent with a genuine endurance training response, not simply a behavioral improvement in negative geotaxis.

### Cardiac performance

We have previously established that exercise training reduces cardiac failure in response to external electrical pacing [24]. External pacing is a cardiac stress assay that paces hearts to twice their normal heartbeat, then measures the percentage of flies that undergo arrest following treatment [44]. The percentage of hearts that undergo arrest following pacing is highly age-dependent [45] and acts as a marker for overall cardiac health. Three-week old *La* flies have a lower failure rate than age-matched unexercised *Ra* flies (Figure 2F) and do not receive further benefit from training (Figure 2F). However, after three weeks of exercise training, Three-week old *Ra* males had a similar failure rate to that of age-matched *La* males, indicating that exercise training in males can mimic the cardioprotective effect seen in *La* flies. Three-week old female *Ra* flies did not respond to training with significant improvement.

Exercise training increases Lysotracker staining in adipose tissue, but not heart, of wild type male flies [26]. Lysotracker staining is also high in adipose tissue of three-week old *La* flies in both males and females, consistent with a genetic increase in fat-specific autophagy levels in the *La* line (Figure 2G, H).

In both the *Ra* and *La* lines, males showed a stronger physiological response to exercise training than females, a phenomenon that we have previously observed across genotypes. This difference is due to a dimorphic behavioral response to the exercise stimulus which will be described in a separate manuscript. Therefore, we concentrated on males for microarray analysis.

### Gene expression profiles of exercised and long-lived flies

Whole-fly transcript levels from untreated three-week old male *Ra* flies were compared to whole-fly transcript levels from untreated three-week old male *La* flies, and separately to whole-fly transcript levels from male *Ra* flies that were subjected to exercise training for the first three weeks of life. Three weeks was selected as a timepoint because it is a time before log-phase of mortality begins in either line, allowing a look at transcript changes occurring during the healthy phase of life, rather than at changes in response to advanced age and increasing pathology. Three weeks of training is sufficient to generate training-induced improvements to mobility and cardiac function in *Ra* flies (Figure 2B, D-F).

**Figure 3:**
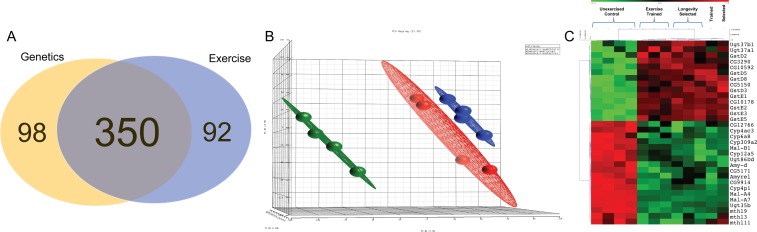
Transcriptional changes induced by exercise training and by selective breeding for longevity are highly overlapping (**A**) Venn diagram showing transcripts altered by breeding from *Ra* to *La* (beige), transcripts altered by exercise training *Ra* males (blue), and the transcripts commonly altered by both interventions (overlap). (**B**) Plot of variance in gene expression explained by each treatment, when each is treated as the principal component (PCA). Training creates an expression pattern in *Ra* that is substantially similar to *La*. *Ra* (green), exercise trained *Ra* (blue) and selectively bred *La* (red). (**C**) Heat map of genes contained in KEGG pathways that are significantly altered by both interventions.

448 genes had altered expression following selective breeding from *Ra* to the *La* line (Supplementary Table S1). 442 genes had altered expression following exercise training in *Ra* flies in comparison to isogenic sibs (Supplementary Table S2). In order to highlight genes that may be involved in the effects of training on healthspan, we looked for the intersection of genes whose transcription was altered similarly by both selective breeding for longevity and by exercise training. 350 genes, or 65% of genes with transcription altered by breeding alone, were altered by both interventions in the same direction (Figure 3A). Orthogonal linear transformations of the input data were performed in order to assess portions of the variability associated with each principal component. The result of this Principal Component Analysis was consistent with the idea that selective breeding and exercise produce a similar pattern of variability in gene expression, represented graphically by the colored clouds in Figure 3B. Members of four KEGG pathways were significantly upregulated by both interventions, including Xenobiotic/Drug metabolism, Glutathione Metabolism and Folate biosynthesis (Table 1, Figure 3C). Members of four KEGG pathways were significantly downregulated by both interventions, including several aspects of carbohydrate metabolism (Table 1, Figure 3C).

**Table 1:** KEGG pathway transcripts altered in the same direction by both endurance exercise and longevity selection

	Pathway	Hits	KEGG ID
Upregulated KEGG pathway genes	metabolism of xenobiotics drug metabolism	10	Ugt37b1, Ugt37a1, [GstD2, GstD5, GstD8, GstD3, GstE1, GstE2, GstE3, GstE5]
	[glutathione metabolism]	8	
	folate biosynthesis	3	CG3290, CG5150, CG10592
Downregulated KEGG pathway genes	starch/sucrose metabolism^•^	8	CG12766^•^, CG9914^•^, CG5171^•^, Mal-A4^•‡^, Mal-B1^•^‡, Amy-d^•‡^, Amyrel^•‡^, Mal-A7^•^*, CG10178*, Ugt35b*, Ug86Dd*
	galactose metabolism^‡^	4	
	pentose/glucuronate interconversions*	4	
	limonene/pinene degradation	5	Cyp4ac3, Cyp6a8, Cyp309a2, Cyp12a5, Cyp4p1

Brackets indicate genes that are members of both the glutathione metabolism and the metabolism of xenobiotics KEGG pathways. Gray upregulated genes are part of the KEGG folate biosynthesis pathway. Dots, crosses and stars indicate membership in downregulated KEGG pathways. Some genes are members of multiple pathways. Gray downregulated genes are members of the KEGG limonene/pinene degradation pathway.

In addition to families identified by KEGG pathway analysis, we also noted several cases where multiple genes involved in similar functions were altered by both interventions. We chose two such functional groups, sensory receptors and methuselah-like genes, and confirmed the expression changes of individual genes from each group by qRT-PCR in order to validate the microarray results (Supplementary Figure 1A-H). These were chosen because they had previously been associated with longevity and movement behaviors, but not specifically linked to exercise capacity. Each is discussed separately below.

### Sensory genes

**Figure 4:**
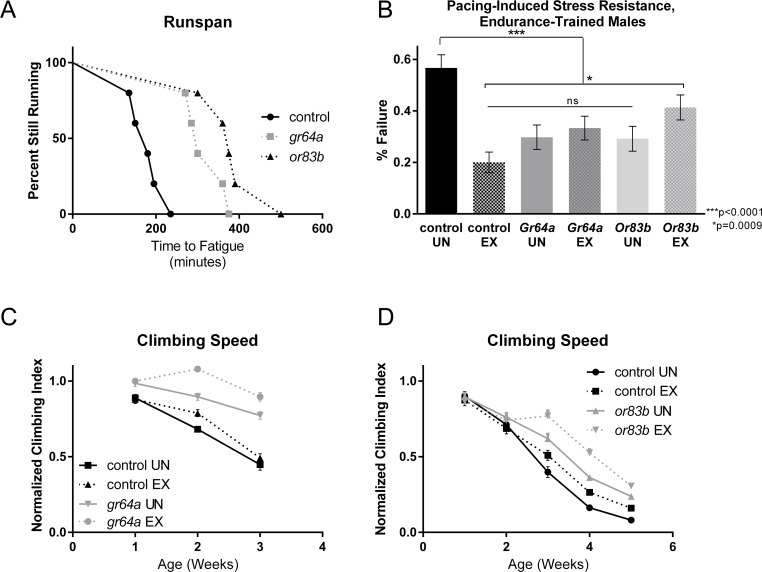
Sensory receptor mutations increase exercise capacity (**A**) Endurance of both *gr64a* and *or83b* mutants is significantly higher than a *w^1118^* background control (log-rank; p <.0001 for each, *n* ≥ 100). (**B**) Exercised *w^1118^* background control males had significantly less cardiac failure in response to electrical pacing stress than did unexercised cohorts (ANOVA with Tukey post-hoc comparison, *p* <.0001). Both *Gr64a* and *Or83b* flies had significantly less cardiac failure than unexercised *w^1118^* controls whether exercised or not (ANOVA with Tukey post-hoc comparison, *p* <.0001). Exercised *Or83b* flies displayed a higher failure rate than exercised background controls (*t*-test, p=.0009) while maintaining a failure rate significantly lower than unexercised *w^1118^* flies (*t*-test, *p* <.0001). (**C**) Unexercised *gr64a* males have a slower decline in negative geotaxis speed than control males (two-way ANOVA, p <.0001). Nevertheless, *gr64a* males still display significant improvement in negative geotaxis following training (two-way ANOVA, p <.0001, *n* ≥ 100). Exercised controls have higher climbing index across ages compared to unexercised controls (two-way ANOVA, p <.0001, *n* ≥ 100). (**D**) Unexercised *or83b* males have a slower decline in negative geotaxis speed than control males (two-way ANOVA, p <.0001, *n* ≥ 100). Nevertheless, *or83b* males still display significant improvement in negative geotaxis following training (two-way ANOVA, p <.0001, *n* ≥ 100). Exercised controls have higher climbing index across ages compared to unexercised controls (two-way ANOVA, p <.0001, *n* ≥ 100).

We noted several changes to the expression of odorant or gustatory receptors in exercised flies, including Or59a, Or59b, Gr98a and Gr32a, leading us to wonder if alterations to nutrient sensing could improve exercise capacity. Since alteration of several receptors was detected, we reasoned that a general reduction in odorant sensing might be induced by exercise. Therefore, we tested a null mutation in *Or83b*, a co-receptor with heteromeric G protein-coupled receptors, including odorant receptors. This mutation is thought to generally reduce detection of a wide variety of odorants [46], [47] and *Or83b* mutants have extended longevity [48]. *Or83b* mutants showed significant improvement over background controls in runspan of one-week old flies (Figure 4A), and in cardiac stress resistance of three-week old flies (Figure 4B). As previously observed [49], *Or83b* mutants also have a preserved climbing index at older ages (Figure 4C).

Because we find that genes involved in carbohydrate metabolism are downregulated by exercise, and because several gustatory receptors showed altered expression in both interventions, we wondered whether reduction in sugar tasting might potentiate exercise capacity. Flies mutant for *Gr64a*, a receptor for sweet taste involved in sensing of carbohydrate moieties [50], [51], showed improvement to runspan in one-week old flies (Figure 4A), to cardiac stress tolerance in three-week old flies (Figure 4B), and to climbing index across five weeks of age (Figure 4C). This result is also consistent with a link between nutrient sensing and exercise behavior.

### Methuselah-like genes

Three members of the G-protein coupled receptor-encoding *methuselah-like* gene family, *mthl-3*, *mthl-9* and *mthl-11* were downregulated by both interventions. As members of this gene family have variously been associated with changes to negative geotaxis behavior and longevity [52]–[54], we tested these genes individually for their ability to extend lifespan or performance. Although wild-type females do not respond to exercise training with physiological improvements, this lack of response is due to their failure to complete the training program, not necessarily due to dimorphic responses to the training (see Discussion). Therefore, we measured the effects of altering *mthl-3* gene expression in both females and males.

**Figure 5:**
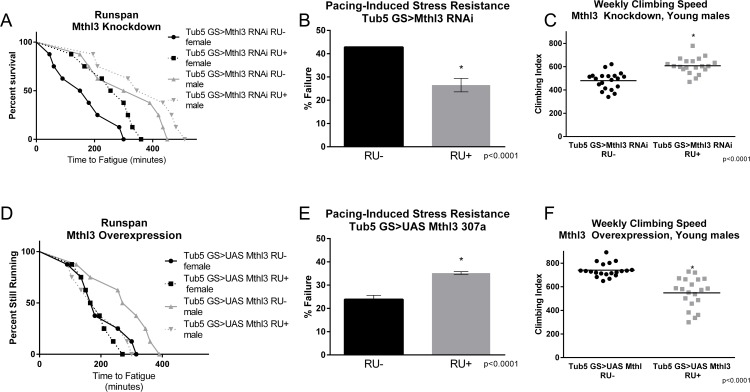
*methuselah-like 3* knockdown mimics healthspan benefits of exercise training (**A**) Ubiquitous expression of RNAi against *mthl3* using an RU-inducible driver increases the endurance of both males (log-rank; p = .0452) and females (log-rank; p <.0001) compared to RU- controls. *n* ≥ 160 for all endurance assays. (**B**) *mthl3* knockdown reduces cardiac failure in response to pacing stress compared to RU- controls (*t*-test; p <.0001, *n* ≥ 100). (**C**) *mthl3* knockdown increases negative geotaxis in males compared to RU- controls (2-way ANOVA; p <.0001, *n* ≥ 100). (**D**) Ubiquitous overexpression of *mthl3* reduces the endurance of males compared to RU- controls (log-rank; p =.0005, *n* ≥ 160). (**E**) Ubiquitous overexpression of *mthl3* increases cardiac failure in response to pacing (error bars indicate ±SD, *t*-test; p <.0001, *n* ≥ 100). (**F**) Ubiquitous overexpression of *mthl3* decreases negative geotaxis in males compared to RU- controls (2-way ANOVA; p <.0001, *n* ≥ 100).

**Figure 6:**
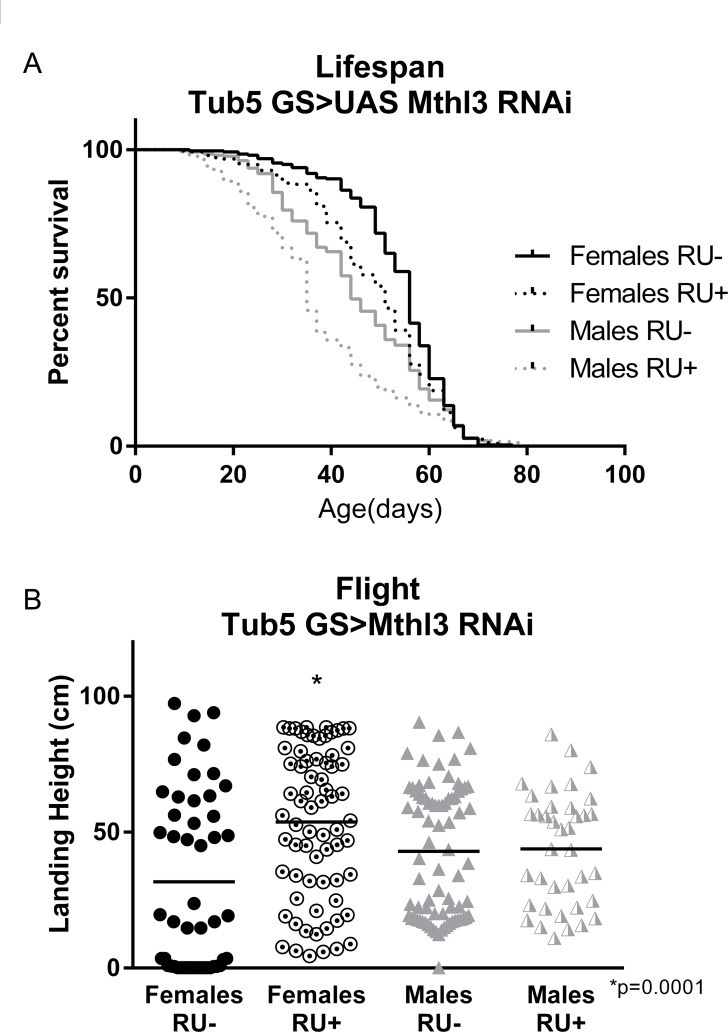
*methuselah-like 3* knockdown fails to extend longevity (**A**) Ubiquitous knockdown of *mthl3* does not extend lifespan, and in fact reduces longevity of both males and females (log-rank; p <.0001 for each, *n* ≥ 260). (**B**) Ubiquitous knockdown of *mthl3* improves flight ability of females compared to RU- control (*t*-test, p = .0001, *n* ≥ 160), but not males.

Knockdown of either *mthl-9* or *mthl-11* by ubiquitous expression of an RNAi construct did not improve climbing index or runspan (data not shown). However, ubiquitous knockdown of *mthl-3* alone in adult flies with an RU-inducible *tub5*-*Gal4* significantly improved runspan (Figure 5A), cardiac stress resistance (Figure 5B) and climbing index (Figure 5C) compared to RU- controls, despite only inducing a partial knockdown (Supplementary Figure S1I). Conversely, ubiquitous overexpression in adult flies (*tub5*-*Gal4* >UAS-*mthl-3*) was sufficient to reduce runspan (Figure 5D), cardiac stress resistance (Figure 5E) and climbing index (Figure 5F) compared to RU- controls. By contrast, overexpression of two different UAS-*mthl-3* lines with *tub5-Gal4* had no significant effect on longevity (Supplementary Figure S2A, C) or flight performance (Supplementary Figure S2B, D). However, RNAi against *mthl-3* somewhat reduced longevity (Figure 6A), while extending three-week old flight performance in females only (Figure 6B).

## DISCUSSION

### Exercise and longevity

These results demonstrate that a three-week training program is sufficient to induce a change in gene expression that confers a gene expression profile resembling that of long-lived flies. This change is associated with a boost to healthspan, as measured by several indices of performance that persist after training, although age-related decline proceeds at a normal rate once exercise is discontinued [24]. These results further support the use of the *Drosophila* model to probe mechanisms of healthspan extension.

Persistent induction of negative geotaxis involves repetitive falling and loud noise, conditions which are likely to induce some degree of stress in experimental flies. Indeed, some degree of early mortality was observed in all training cohorts. This mortality was sporadic and not consistently different between genotypes, or between sibs that trained and sibs that were placed on the training device but prevented from running. This strongly suggests that increased mortality in flies of all genotypes during training is likely to be caused by acute damage (*e.g*. damage to a leg causing flies to get stuck in food), and not by a systemic stress response. Importantly, this result strongly excludes the possibility that the Power Tower training device has a “selection” effect that removes the weakest flies from the cohort. Nevertheless, all “unexercised” groups throughout the study, including the flies used for the arrays, were placed on the machine at the same time as “exercised” groups, but prevented from running by a foam stopper. This controlled for any potential exercise-independent effects of the training regimen.

### Individual genes

*Or83b* mutant phenotypes are similar to those seen in dietary restricted flies, and are thought to act through similar mechanisms [48]. Dietary restriction also increases exercise capacity acutely, both in negative geotaxis assays and in runspan [39]. Furthermore, longevity extension of *La* flies acts through mechanisms that overlap with dietary restriction [36]. These results are consistent with a link between the sensing of food odors and exercise behavior. In cases where the sensory perception of food is reduced, flies increase movement behavior, exercise capacity and longevity.

Several genes encoding odorant binding proteins are altered specifically by selective breeding, but not exercise, including *Obp99b*. Interestingly, *Obp99b* has recently been associated with extended longevity and proposed to act as a humoral signaling factor [55]. A possible role for *Obp99b* in mediating longevity in *La* flies warrants further study.

These results implicate *mthl-3* as a potentially important mediator of a subset of exercise-induced improvements to healthspan. *mthl-3* is a member of the *methuselah* family of Secretin-like G-protein coupled receptors. Mutation of the founding member of this family, *methuselah*, has been associated with increased longevity [56] and extended sensorimotor function [57]. *mthl-3* has previously been identified as a gene whose transcription is increased during aging [58], but *mthl-3* has not previously been examined for longevity or motor phenotypes.

Here, we observe that *mthl-3* is downregulated by exercise training in *Ra* flies and reduced in *La* flies. Endurance, negative geotaxis and cardiac performance were significantly improved by knockdown of *mthl-3.* Unlike the original *methuselah* or its ligand *stunted* [59], knockdown of *mthl-3* did not significantly extend lifespan, and instead somewhat reduced it. While this result may be surprising in this context, it is important to note that two other *mthl* genes were similarly downregulated by both exercise and selective breeding. It may be that the combined effects of several *mthl* knockdowns would have a different effect on longevity and/or healthspan. It is also possible that *mthl-3* would extend longevity under different temperature conditions or different activity levels, as the effects of *methuselah* are conditional [60], [61]. Alternately, *mthl-3* may simply contribute to healthy physiology without extending longevity.

In addition to the individually tested genes above, we also noted several other interesting trends in the array data that would be useful to follow up further in the future. We discuss these separately below.

### Lipases

Although microarrays were performed on whole flies, one gene classification that was highly upregulated in both interventions was lipases. Several of these are normally enriched in gut, where they contribute to breakdown of food components, including *magro*, an important gene for homeostasis of both triglycerides and cholesterol [62]. Declining function of the fly gut has recently been described as a highly relevant biomarker for aging and an efficient predictor of time of death [63]. Lipase expression in enterocytes has also been proposed to be important in maintenance of gut stem cell function in flies, with JNK and Foxo activity as important intermediates [64], [65]. Interestingly, the exercise-associated PGC-1αhomolog, *spargel*, has been shown to extend lifespan when activated in the gut [63], suggesting that the gut may be a highly relevant target tissue for exercise-induced changes in gene expression.

### Folate metabolism

Folate biosynthesis is a significantly upregulated KEGG pathway in both interventions. This is intriguing in light of other recent results identifying folate metabolism as upregulated in the rapid response to dietary restriction in flies [66]. In *C. elegans*, knockdown of *SAMe*, a folate cycle inhibitor, increases lifespan by a mechanism that is not additive with dietary restriction [67], [68] In addition, the mechanism by which metformin extends lifespan in *C. elegans* is dependent on modifications to folate metabolism in the worms' *E. coli* food source [69]. Folate is an essential precursor to methionine, and thus necessary for methylation [70]. Hypomethylation has been proposed to be a general feature in metazoan aging [71]. Taken together, these results may point to availability of methylation precursors as a common target in multiple anti-aging mechanisms, including dietary restriction, endurance exercise, selective breeding for longevity, and metformin treatment.

### Stress response

*La* flies have previously been observed to be resistant to multiple forms of stress [40, 72–75], and this is reflected in the upregulation of genes in KEGG pathways that promote stress resistance. Here we observe that endurance training also upregulates a set of genes in common with *La* that are associated with stress resistance pathways. These include Xenobiotic metabolism, drug metabolism, and glutathione metabolism genes (Table 1). Glutathione metabolism is particularly intriguing here, since endurance exercise has been proposed to have a hormetic effect on oxidative stress resistance in vertebrates [76]–[78].

### Autophagy

It is perhaps surprising that we did not identify upregulation of pathways associated with autophagy in exercised flies. We have observed upregulation of lysosomal activity in adipose tissue of both *La* flies and exercised *Ra* flies (Figure 2G, H), and we have previously observed similar upregulation in wild-type exercised animals [26].

Upregulation of autophagy in muscle has been proposed to be an important mediator of the benefits of exercise in vertebrate muscle [19], [22]. Fly cardiac muscle mitochondria show evidence of less accumulation of oxidative damage in chronically exercised flies [27], consistent with increased mitophagy in cardiac muscle with exercise. In mutant flies with increased intra-myocellular lipid stores, cardiac muscle displays increased autophagy [26], but exercise training reverses lipid accumulation and lowers autophagy correspondingly in this model.

These results are consistent with a model in which autophagy in muscle is only upregulated in a transitory fashion by exercise, and autophagy genes are not chronically upregulated in muscle by training. Although upregulation in adipose tissue appears to be continuous during exercise training, this tissue alone may not be sufficient to provide statistically significant upregulation in a whole-animal gene expression analysis.

### Sexual dimorphism

Ra flies show a strong sexual dimorphism in response to exercise (Figure 2A-D), as we have previously observed for several other wild-type genetic backgrounds. Males respond to training with consistent improvement in several functional assays, while females typically show a reduced response or no response. The lack of female response is not due to a reduced initial capacity, because initial negative geotaxis speed is nearly identical between males and females. Instead, we have observed sex-specific differences in the behavioral response to repeated negative geotaxis stimuli, with females frequently pausing or failing to respond. This is consistent with exercise experiments in other model systems that also show substantial effects of sex, age, and diet on both the amount of voluntary exercise and on the resulting adaptations, e.g. [79], [80]. Interestingly, knockdown of *mthl3* improves runspan of both sexes, suggesting that females could benefit from training if they successfully executed the program long enough to induce the gene expression changes observed in males.

### Conserved effects of exercise/selective breeding

The large degree of overlap between genes induced by exercise and those induced by breeding for longevity is highly suggestive of common genetic mechanisms. This is consistent with work from a rodent model in which rats were selectively bred for oxidative capacity based on performance on a treadmill test. High capacity running (HCR) rats also gained an extension of longevity in the process [81]. Thus, breeding for longevity confers exercise capacity and vice versa.

HCR rats also demonstrated improved cardiac performance and delayed cardiac senescence [81], as did *La* flies. Furthermore, improvements to aerobic efficiency were also observed in both models [38], [82]. Aging mitochondria *in vivo* are also protected from accumulated oxidation exposure by exercise training in both mouse skeletal muscle and fly cardiac muscle [27], indicating that the relationship between longevity, mitochondrial function and exercise capacity is highly conserved.

## MATERIALS AND METHODS

### Fly stocks and maintenance

All fly lines were reared and aged at 25°C; 50% humidity with a 12 hour light-dark cycle and provided with a standard 10% yeast/10% sucrose diet. Brewer's Yeast was obtained from MP Biomedicals (Solon, OH). *Or83b* and *Gr64a* lines were provided by Scott Pletcher and John Carlson, respectively [48], [50] and backcrossed into a *w^1118^*background. *UAS-mthl3(307a)* and *UAS-mthl3(365)* were provided by Mark Vanberkum. *UAS-mthl3 RNAi (Bloomington Stock #36822), Tub5* GS *gal-4* and y^1^w^1^ flies were obtained from the Bloomington *Drosophila* Stock Center. All UAS and Gal4 containing lines were backcrossed into the *y^1^w^1^* background for ten generations before analysis.

### Microarray analysis

Total RNA was extracted using Trizol (Invitrogen, Carlsbad, CA, USA) for each genotype. RNA was then column purified using the RNeasy Plus Mini Kit (QIAGEN, Redwood City, CA, USA). At least three independent RNA extractions were prepared for each sample. Total RNA was analyzed by the Wayne State University School of Medicine Karmanos Cancer Institute using *Drosophila* GeneChip (Affymetrix) assay. The Affymetrix expression image files were uploaded to Partek^®^ software, version 6.6 Copyright © 1993–2012 Partek Inc., St. Louis, MO, USA. Data was normalized using the quantile normalization method and the RMA background subtraction was applied. Differentially expressed genes were identified using a 1-way ANOVA. Genes were filtered according to differential *p*-value with FDR correction < 0.05 [83] as well as fold-change +/− 1.5. Heatmaps were generated using TMeV [84] software with Pearson Correlation distance metric.

### qRT-PCR

Total RNA was prepared from 20 whole, age-matched flies of indicated genotype and treatment using Trizol (Invitrogen, Carlsbad, USA). At least three independent RNA extractions were prepared for each sample. Relative message abundance was determined by amplification and staining with SYBR Green I using an ABI 7000 SDS (Applied Biosystems). Expression of Rp49 was used for normalization. Differences between genotypes were assessed by *t*-test or nested ANOVA. Primer sequences are listed below.

Mthl3:

Forward: 5′-GATCCCCGCCCATTTGACAG-3′

Reverse: 5′-GGCTCGCCACCTTCTCCTTC-3′

Mthl9:

Forward: 5′-TACGCCCACACGGTCAACAT-3′

Reverse: 5′-GCCCGGTACTCCACTCCATC-3′

Mthl11:

Forward: 5′-GCAAGCGGTGGGTTTTCTGT-3′

Reverse 5′-TTCTACGTCGGCCATTTTCTCA-3′

Gr64a:

Forward: 5′-ACGGCGGCGGACATCAAT-3′

Reverse: 5′-CTCCACCTCGACGCACCAG-3′

Gr98a:

Forward: 5′-CATGCGGCGACTGATGAAGTGT-3′

Reverse: 5′-CGAAGCTGAAGCGCCAGTAGC-3′

Gr32a:

Forward: 5′-TCGCATCGGCTTTGCTCAGG-3′

Reverse: 5′-CGCCTCGCTCGTGCTCCAC-3′

Or59b:

Forward: 5′-GCCGGGCGAGTTCCTTACCT-3′

Reverse: 5′-CGTTCGCCAGCCTCTTGTCC-3′

Or83b:

Forward: 5′-CCAGAAAGAACAGCTTCCTCATCT-3′

Reverse: 5′-CGAGTCGGATGCTCGTTACC-3′

Rp49:

Forward: 5′-ACTCAATGGATACTGCCCAAGAA-3′

Reverse: 5′-CAAGGTGTCCCACTAATGCATAAT-3′

### Survival analysis

Prior to all experiments, fly cultures were maintained at a constant density for at least two generations. 15 virgin females and 5 males were mated in 300 mL bottles with 50 mL standard 10% sucrose 10% yeast, with or without mifepristone or vehicle as described in text/figures [85]. Adult progeny were synchronized by collecting within 2 hours of eclosion, over a 24 hour time period. During and after three weeks of exercise training, groups of 20 age and gender-matched exercised or control unexercised flies were transferred into narrow polypropylene vials containing 5mL of appropriate food medium. Food vials were changed every second day, at which time dead flies were removed and counted. Flies were housed in a 25°C incubator on a 12:12 light:dark cycle at 50% relative humidity. Differences in survival were analyzed by log-rank. *n* > 280 for all longevity experiments.

### Endurance exercise

Exercise was performed as previously described [23], [86]. Cohorts of at least 320 flies were collected under light CO_2_ anesthesia within 2 hours of eclosion and separated into vials of 20. Flies were then further separated into 2 large cohorts of at least 160 flies divided into exercised and unexercised groups. The unexercised groups were placed on the exercise training device, but were prevented from running by the placement of a foam stopper low in the vial in order to control for exercise-independent environmental factors. Exercised flies were placed in identical vials with normal cotton stoppers. The exercise device drops the vials of flies every 15 seconds, inducing a repetitive, innate negative geotaxis response. Exercised flies are free to run to the top of the vial. A ramped program of gradually increasing daily exercise time was previously established to generate significant alterations in mobility and cardiac performance [24].

### Negative geotaxis behavior

Adult flies were collected with light CO_2_ anesthesia within 2 hours of eclosion and housed in appropriate fresh food vials. Negative geotaxis was assessed in Rapid Negative Geotaxis (RING) assays in groups of 100–120 flies as described [87]. Flies were transferred to individual polypropylene vials in a RING apparatus and allowed to equilibrate for 1 minute. Negative geotaxis was elicited by sharply rapping the RING apparatus four times in rapid succession. The positions of the flies were captured in digital images taken 2s after eliciting the behavior. Images were analyzed using ImageJ. The relative distance climbed by each fly was converted into quadrants using Microsoft Excel. The performance of 20 flies was calculated as the average of four consecutive trials to generate a single datum. Flies were tested 5 times per week for 5 weeks to assess decline in negative geotaxis speed with age. Between assessments, flies were returned to food vials and housed until the following RING test. Negative geotaxis results were analyzed using two-way ANOVA analysis with *post hoc* Tukey multiple comparison tests in GraphPad Prism (San Diego, CA, USA).

### Endurance

Climbing endurance was measured using the fatigue assay described previously [23]. Eight vials of flies from each cohort were subjected to the fatigue assay at two time points: once in the first week of life, and once in the fourth week of life. For each assessment, the flies were placed on the Power Tower exercise machine and made to climb until they were fatigued, or no longer responded to the negative geotaxis stimulus. Monitored at 15 min intervals, a vial of flies was visually determined to be “fatigued” when five or fewer flies could climb higher than 1 cm after four consecutive drops. The time from the start of the assay to the time of fatigue was recorded for each vial, and the data analyzed using log-rank analysis in Prism.

### Flight performance

Flight was analyzed as described in Babcock *et al.* [88]. Cohorts of at least 160 flies were aged and/or exercise trained in narrow vials housing groups of 20 age-matched siblings. Acrylic sheeting with paintable adhesive was placed in the flight tube, and fly cohorts were ejected into the apparatus to record flight performance and subsequent landing height after release. Fly cohorts were introduced to the flight tester one vial at a time using a gravity-dependent drop tube in order to reduce variability [88]. After a full cohort of flies was captured on the adhesive, the sheeting was removed to a white surface in order to digitally record the landing height of each fly. Images were analyzed using ImageJ. Landing height was averaged and compared in Prism using a student *t*-test.

### Electrical pacing

Once per week a minimum of 100 males and 100 females were removed from the cohort and subjected to electrical pacing as described [44]. The percentage of fly hearts that responded to pacing with either fibrillation or arrest was recorded as “% failure”. Percent failure is a marker for stress sensitivity [44]. Data were analyzed in Prism using student's *t*-test.

### Lysotracker

Lysotracker staining of adult fat bodies was performed as in Sujkowski *et al.* [26] Adult flies separated by age, genotype, and or treatment were dissected, ventral side up, in room temperature PBS. Having exposed fat bodies, partially dissected flies were rinsed 1X in fresh PBS. Lysotracker green (Molecular Probes, Eugene, OR) was diluted to 0.01 μM in PBS and applied to dissected preps for 30 seconds. Samples were washed 3 times in fresh PBS. Stained fat bodies were subsequently removed and mounted in Vectashield reagent (Vector Laboratories, Burlingame, CA, USA). Confocal work was done at the Microscopy, Imaging and Cytometry Resources Core at Wayne State University, School of Medicine on a Zeiss Laser Scanning LSM 780 (Jena, Germany) using a 100X oil immersion objective. Images were analyzed using ImageJ. A minimum of 10 samples were analyzed for each sample. Data were subjected to student *t*-test following quantification.

## SUPPLEMENTARY INFORMATION FIGURES AND TABLES








