# Supplementary material for: Endurance exercise and selective breeding for longevity extend Drosophila healthspan by overlapping mechanisms
Source: Aging (Albany NY). 2015 Aug 8;7(8):535–50. doi: 10.18632/aging.100789 (PMC4586100; doi:10.18632/aging.100789)
Supplement: Supplementary file 1 [file aging-07-535-s001.pdf]

SUPPLEMENTARY INFORMATION

Please browse the full text version of this manuscript to see the Supplementary Tables S1-S3.

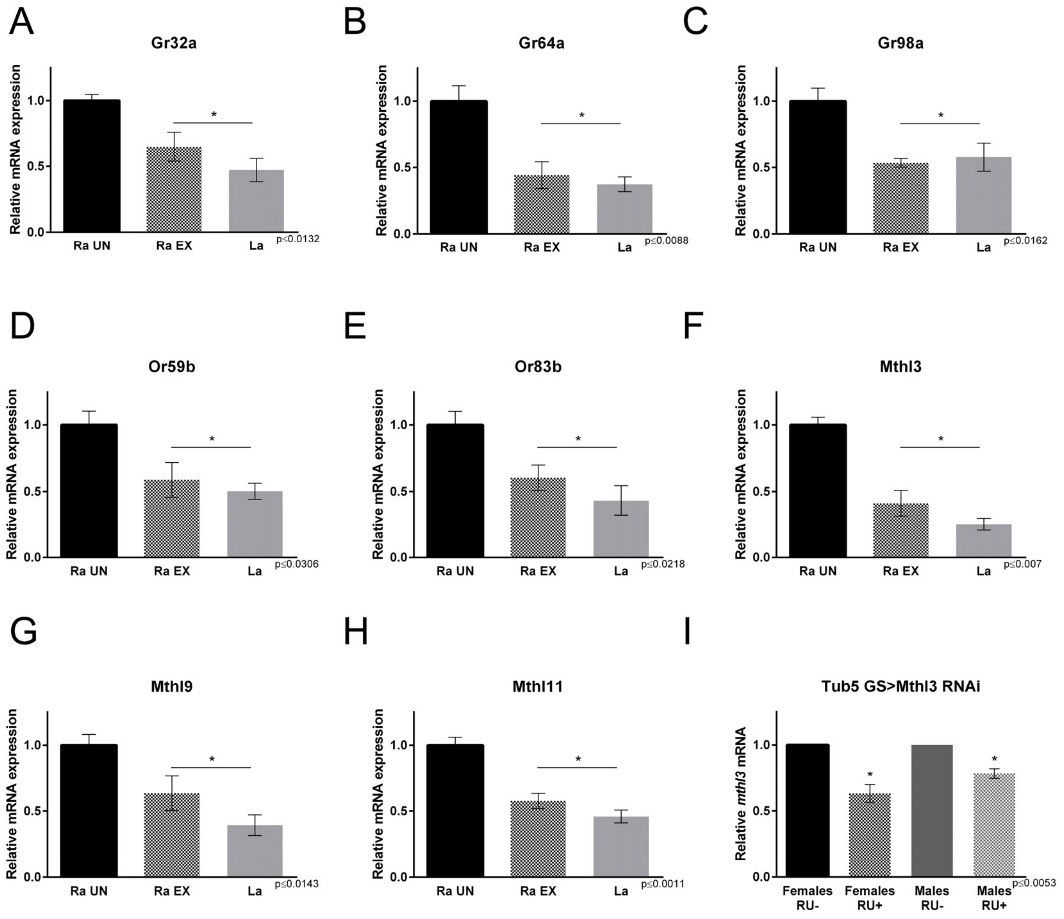

Supplementary Figure S1. qRT-PCR confirmation of microarray results. RNA was isolated from 3-week old Ra unexercised, 3-week old Ra exercised and 3-week old La flies. Gene expression levels from the following genes were compared. *Gr32a* (**A**), *Gr64a* (**B**), *Gr98a* (**C**), *Or59b* (**D**), *Or83b* (**E**), *Mthl3* (**F**), *Mthl9* (**G**), *Mthl11* (**H**). In all cases exercised Ra and La had significantly lower expression than unexercised Ra flies (t-test, p<.0362). (**I**) *Tub5 GS>UAS Mthl3* RNAi flies were collected and exposed to either RU486 (RU+) or vehicle (RU-) for 5 days prior to RNA isolation and qRT-PCR analysis. RU+ flies had significantly lower *Mthl3* expression than RU- controls (t-test, p≤.0053).

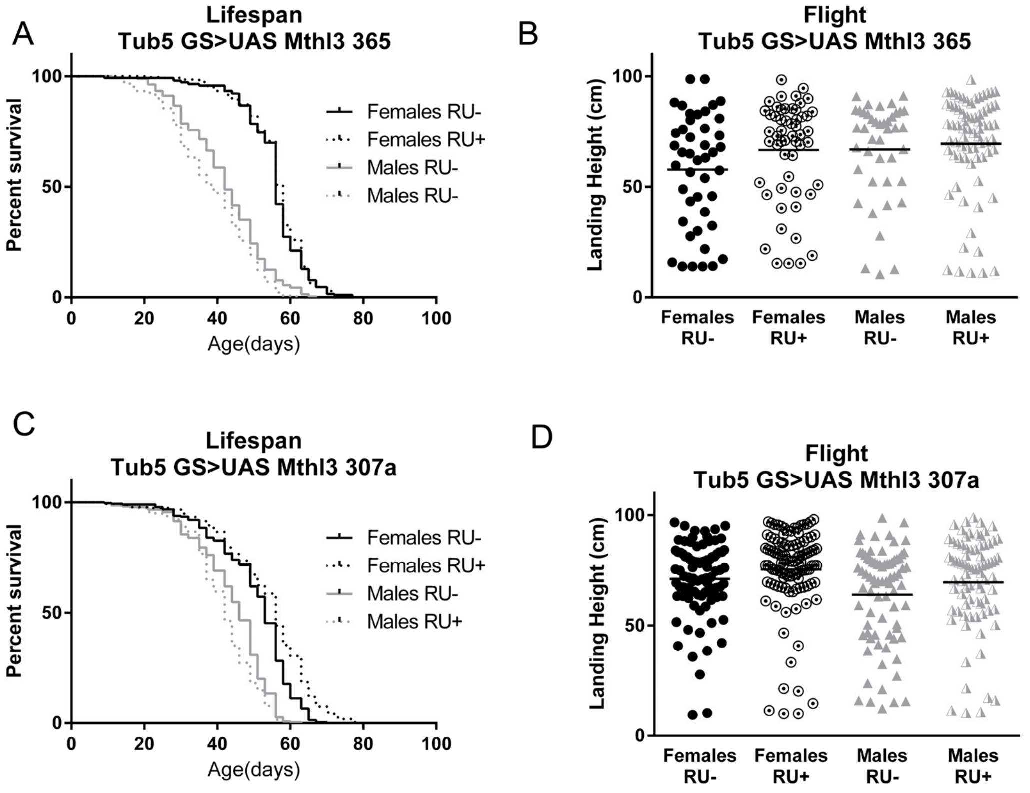

Supplementary Figure S2. Overexpression of *methuselah-like 3* does not affect longevity or flight ability. (**A**, **C**) Ubiquitous overexpression of *mthl3* does not significantly alter longevity of males or females compared to RU- controls using either of two lines containing UAS-*mthl3* at different insertion sites. (**B**, **D**) Ubiquitous overexpression of *mthl3* does not significantly alter flight ability of males or females compared to RU- controls using either of two lines containing UAS-*mthl3* at different insertion sites.
