# Supplementary material for: Endurance exercise and selective breeding for longevity extend Drosophila healthspan by overlapping mechanisms
Source: Aging (Albany NY). 2015 Aug 8;7(8):535–50. doi: 10.18632/aging.100789 (PMC4586100; doi:10.18632/aging.100789)
Supplement: Supplementary file 2 [file aging-07-535-s002.docx]

**Supplementary Table S1**

| Probe | Symbol | Description | GenBank | Gene |
| --- | --- | --- | --- | --- |
| 18149978 | CG12934 | CG12934 | NM_136757 | 36124 |
| 18173516 | snoRNA:CG32479-b | ncRNA | NR_048214 | 12798553 |
| 18158990 | Mal-A7 | Maltase A7 | NM_136539 | 35829 |
| 18156154 | Cyp6a2 | Cytochrome P450-6a2 | NM_078904 | 35587 |
| 18178314 | Cpr72Ec | Cuticular protein 72Ec | NM_140627 | 39816 |
| 18156279 | Lcp1 | Larval cuticle protein 1 | NM_057271 | 35817 |
| 18141036 | CG11911 | CG11911 | NM_134674 | 33206 |
| 18146555 | CR43263 | ncRNA | NR_047871 | 12797874 |
| 18149502 | Mal-A4 | Maltase A4 | NM_136537 | 35827 |
| 18190346 | Npc2e | Niemann-Pick type C-2e | NM_169323 | 326136 |
| 18151339 | CG10910 | CG10910 | NM_137436 | 37052 |
| 18189615 | CG18754 | CG18754 | NM_144395 | 59145 |
| 18182534 | osk | oskar | NM_169248 | 41066 |
| 18141537 | CG16704 | CG16704 | NM_134953 | 33589 |
| 18147926 | AttA | Attacin-A | NM_079021 | 36636 |
| 18151405 | GstE1 | Glutathione S transferase E1 | NM_137479 | 37106 |
| 18180537 | CG33969 | CG33969 | NM_001038945 | 40252 |
| 18205966 | Yp1 | Yolk protein 1 | NM_078548 | 31939 |
| 18158457 | CG11211 | CG11211 | NM_136364 | 35545 |
| 18172304 | CG8628 | CG8628 | NM_139826 | 38783 |
| 18142869 | CG5846 | CG5846 | NM_135489 | 34326 |
| 18135984 | hgo | homogentisate 1,2-dioxygenase | NM_078820 | 34552 |
| 18215100 | CG9360 | CG9360 | NM_132518 | 32128 |
| 18159265 | CG1888 | CG1888 | NM_136651 | 35979 |
| 18133827 | CG11034 | CG11034 | NM_135117 | 33810 |
| 18161213 | CG16898 |  | NM_137608 | 37276 |
| 18143473 | CG9377 | CG9377 | NM_135796 | 34743 |
| 18146471 | CG42876 | CG42876 | NM_001201889 | 10178914 |
| 18192300 | CG43175 | CG43175 | NM_001260225 | 12798449 |
| 18218028 | snoRNA:Psi28S-3436a | ncRNA | NR_002464 | 3772323 |
| 18198686 | fit | female-specific independent of transformer | NM_142736 | 42578 |
| 18156749 | epsilonTry | epsilonTrypsin | NM_080373 | 49080 |
| 18153106 | AttC | Attacin-C | NM_079005 | 36484 |
| 18132600 | CG15263 | CG15263 | NM_135904 | 34898 |
| 18217524 | Muc4B | Mucin 4B | NM_167006 | 31368 |
| 18160806 | CG10936 | CG10936 | NM_137395 | 37000 |
| 18188283 | CG11854 | CG11854 | NM_143101 | 43037 |
| 18168689 | CG18180 | CG18180 | NM_140084 | 39125 |
| 18212963 | Ser6 | Serine protease 6 | NM_078702 | 33073 |
| 18216520 | CG15618 | CG15618 | NM_134517 | 33001 |
| 18140731 | nimB2 | nimrod B2 | NM_165075 | 260645 |
| 18186819 | CG14322 | CG14322 | NM_142385 | 42124 |
| 18141290 | CG3597 | CG3597 | NM_134830 | 33420 |
| 18192535 | CR43475 | ncRNA | NR_048466 | 12798512 |
| 18188365 | CG5107 | CG5107 | NM_143144 | 43084 |
| 18131045 | ninaD | neither inactivation nor afterpotential D | NM_165237 | 326160 |
| 18171442 | Drsl2 | Drosomycin-like 2 | NM_168019 | 38408 |
| 18180657 | snoRNA:Psi28S-1837b | ncRNA | NR_003860 | 5740763 |
| 18178712 | Cyp12c1 | CG4120 | NM_140795 | 40037 |
| 18135842 | Tsp39D | Tetraspanin 39D | NM_078888 | 35405 |
| 18149971 | Elp2 | Elongator complex protein 2 | NM_136756 | 36123 |
| 18192073 | CG42821 | CG42821 | NM_001038971 | 3885579 |
| 18174210 | Ubi-p63E | Ubiquitin-63E | NM_079185 | 38456 |
| 18148567 | IM2 | Immune induced molecule 2 | NM_166277 | 49802 |
| 18182240 | Act88F | Actin 88F | NM_079643 | 41885 |
| 18149383 | CG2064 | CG2064 | NM_136466 | 35708 |
| 18203391 | CG43166 | CG43166 | NM_001260364 | 12798255 |
| 18210765 | CG34026 | CG34026 | NM_001038750 | 3885569 |
| 18135978 | lectin-28C | CG7106 | NM_144379 | 53542 |
| 18161552 | CG4363 | CG4363 | NM_137769 | 37488 |
|  |  |  |  |  |
| 18165360 | Est-6 | Esterase 6 | NM_176322 | 39392 |
| 18208265 | Obp8a | Odorant-binding protein 8a | NM_167184 | 31860 |
| 18189219 | CG15539 | CG15539 | NM_143547 | 43627 |
| 18153188 | PGRP-SC2 | CG14745 | NM_136566 | 35862 |
| 18186681 | CG17560 | CG17560 | NM_142310 | 42022 |
| 18187580 | CG7054 | CG7054 | NM_142793 | 42643 |
| 18190815 | snoRNA:Psi28S-3405b | ncRNA | NR_003908 | 5740543 |
| 18161522 | pirk | poor Imd response upon knock-in | NM_137754 | 37468 |
| 18145006 | CG31955 | CG31955 | NM_164552 | 319045 |
| 18157154 | Cyp9b1 | Cytochrome P450-9b1 | NM_078921 | 35634 |
| 18197461 | CG6912 | CG6912 | NM_142162 | 41820 |
| 18197578 | CG6125 | CG6125 | NM_169656 | 41882 |
| 18153861 | CG30411 | CG30411 | NM_166605 | 246600 |
| 18197227 | CG14369 | CG14369 | NM_142038 | 41660 |
| 18163021 | CG30392 | CG30392 | NM_137728 | 246587 |
| 18159997 | CG13324 | CG13324 | NM_136989 | 36434 |
| 18134388 | CG13091 | CG13091 | NM_135397 | 34188 |
| 18208389 | CG15209 | CG15209 | NM_132426 | 32011 |
| 18155112 | CG42566 | CG42566 | NM_001103951 | 8674120 |
| 18171337 | edin | elevated during infection | NM_168735 | 317900 |
| 18216848 | CG3690 | CG3690 | NM_130520 | 31047 |
| 18160937 | IM23 | Immune induced molecule 23 | NM_137474 | 37099 |
| 18142324 | CG7025 | CG7025 | NM_135289 | 34042 |
| 18157486 | Amyrel | CG8221 | NM_057914 | 36863 |
| 18187155 | CG4783 | CG4783 | NM_142594 | 42381 |
| 18131750 | Lectin-galC1 | Galactose-specific C-type lectin | NM_057852 | 35216 |
| 18205607 | Lsp1alpha | Larval serum protein 1 alpha | NM_078583 | 32199 |
| 18163701 | CG34216 | CG34216 | NM_001103743 | 5740103 |
| 18155662 | CG43188 | CG43188 | NM_001259331 | 12798403 |
| 18153190 | PGRP-SC1a | CG14746 | NM_136563 | 35859 |
| 18155614 | CG43114 | CG43114 | NM_001259335 | 12798406 |
| 18202278 | CG34291 | CG34291 | NM_001104468 | 5740331 |
| 18181599 | CG43085 | CG43085 | NM_001259889 | 12797926 |
| 18206366 | Spat | Serine pyruvate aminotransferase | NM_078507 | 31587 |
| 18164218 | CG30059 | CG30059 | NM_137028 | 246425 |
| 18150304 | Cpr49Ag | Cuticular protein 49Ag | NM_136932 | 36353 |
| 18207870 | Apc7 | Anaphase promoting complex 7 | NM_132101 | 31594 |
| 18153043 | CG17580 | CG17580 | NM_144127 | 50228 |
| 18168685 | CG8329 | CG8329 | NM_140082 | 39123 |
| 18185797 | HisCl1 | Histamine-gated chloride channel subunit 1 | NM_169429 | 41426 |
| 18177554 | CG11652 | CG11652 | NM_168471 | 39317 |
| 18195001 | Ugt35b | UDP-glycosyltransferase 35b | NM_079589 | 41333 |
| 18188602 | CG17189 | CG17189 | NM_143277 | 43263 |
| 18199522 | CG10560 | CG10560 | NM_143127 | 43065 |
| 18199618 | CcapR | Cardioacceleratory peptide receptor | NM_206574 | 2768688 |
| 18170639 | CG11131 | CG11131 | NM_141172 | 40511 |
| 18182621 | Jon99Ciii | Jonah 99Ciii | NM_170451 | 43543 |
| 18172117 | CG34391 | CG34391 | NM_001104038 | 5740816 |
| 18176593 | CG10477 | CG10477 | NM_139753 | 38679 |
| 18190088 | LSm3 | CG31184 | NM_170107 | 42842 |
| 18154453 | CG34227 | CG34227 | NM_001103799 | 36186 |
| 18175633 | mthl9 | methuselah-like 9 | NM_138185 | 38056 |
| 18144307 | lectin-24A | CG3410 | NM_144380 | 53543 |
| 18153198 | Uhg1 | U snoRNA host gene 1 | NR_001754 | 3772106 |
| 18198987 | CG4408 |  | NM_142884 | 42762 |
| 18184424 | CG1092 | CG1092 | NM_164325 | 40538 |
| 18188807 | CG9989 | CG9989 | NM_143370 | 43390 |
| 18207981 | CG4593 | CG4593 | NM_132149 | 31649 |
| 18144513 | Obp22a | Odorant-binding protein 22a | NM_001014457 | 246672 |
| 18137060 | lectin-37Da | CG33532 | NM_001014489 | 3346222 |
| 18150004 | RpS15Ab | Ribosomal protein S15Ab | NM_136772 | 36142 |
| 18193419 | Jon99Cii | Jonah 99Cii | NM_079830 | 43544 |
| 18208270 | CG15369 | CG15369 | NM_132314 | 31862 |
| 18165929 | mtrm | matrimony | NM_139966 | 38958 |
| 18170952 | Nplp3 | Neuropeptide-like precursor 3 | NM_144453 | 59235 |
| 18144930 | CG31832 | CG31832 | NM_165098 | 318970 |
| 18205969 | Yp3 | Yolk protein 3 | NM_078593 | 32339 |
| 18167122 | CG9168 | CG9168 | NM_138262 | 38163 |
| 18216382 | CG8028 | CG8028 | NM_133147 | 32923 |
| 18132911 | nAcRbeta-21C | nicotinic acetylcholine receptor beta 21C | NM_080359 | 33228 |
| 18167954 | CG5150 | CG5150 | NM_139720 | 38633 |
| 18210607 | CG32698 | CG32698 | NM_001258664 | 31915 |
| 18169211 | CG10741 | CG10741 | NM_140401 | 39521 |
| 18192125 | mir-2494 | mir-2494 stem loop | NR_048409 | 12798429 |
| 18140214 |  |  | NM_078753 |  |
| 18190740 | CG18749 | CG18749 | NM_001031983 | 3771984 |
| 18136636 | CG31704 | CG31704 | NM_164973 | 318906 |
| 18161878 | Or59b | Odorant receptor 59b | NM_079098 | 37715 |
| 18171545 | CG32368 | CG32368 | NM_168244 | 317997 |
| 18213397 | CG18031 | CG18031 | NM_143764 | 45707 |
| 18144431 | Cyp309a2 | CG18559 | NM_134845 | 33439 |
| 18176519 | CG10592 | CG10592 | NM_139719 | 38632 |
| 18198450 | CG4335 | CG4335 | NM_142629 | 42421 |
| 18131649 | Try29F | Trypsin 29F | NM_078794 | 34226 |
| 18154273 | GstE2 | Glutathione S transferase E2 | NM_137480 | 37107 |
| 18147667 | Mdr50 | Multi drug resistance 50 | NM_079016 | 36582 |
| 18216707 | CG14615 | CG14615 | NM_134615 | 33129 |
| 18151514 | DptB | Diptericin B | NM_079063 | 37184 |
| 18147689 | betaTry | betaTrypsin | NM_080165 | 47901 |
| 18176684 | eIF4E-4 | CG10124 | NM_139795 | 38743 |
| 18164663 | CG43103 | CG43103 | NM_001259456 | 12798325 |
| 18168687 | CG18179 | CG18179 | NM_140083 | 39124 |
| 18216879 | eIF4E-7 | CG32859 | NM_166870 | 31059 |
| 18200456 | CG15553 | CG15553 | NM_143584 | 43677 |
| 18151443 | CG10924 | CG10924 | NM_001103897 | 37130 |
| 18207604 | CG15784 | CG15784 | NM_131995 | 31461 |
| 18151163 | CG6967 | CG6967 | NM_166214 | 36922 |
| 18156712 | Def | Defensin | NM_078948 | 36047 |
| 18210813 | GlcAT-I | CG32775 | NM_167003 | 251900 |
| 18138552 | CR43420 | ncRNA | NR_047990 | 12798000 |
| 18153181 | Obp57b | Odorant-binding protein 57b | NM_166394 | 246669 |
| 18174309 | LysX | Lysozyme X | NM_079157 | 38122 |
| 18176029 | Cyp4d20 | CG16761 | NM_139466 | 38311 |
| 18151402 | CG18107 | CG18107 | NM_145336 | 37101 |
| 18154281 | Act57B | Actin 57B | NM_079076 | 37368 |
| 18189936 | CG31091 | CG31091 | NM_170248 | 318591 |
| 18200433 | CG15546 | CG15546 | NM_143574 | 43659 |
| 18167389 | CG1143 | CG1143 | NM_139478 | 38329 |
| 18214931 | Psf3 | CG2222 | NM_132396 | 31967 |
| 18169554 | CG5895 | CG5895 | NM_140571 | 39752 |
| 18140949 | Spn28F | Serpin 28F | NM_080218 | 49807 |
| 18143158 | Ast-CC | Allatostatin double C | NM_135639 | 34538 |
| 18153511 | CG30154 | CG30154 | NM_145874 | 246487 |
| 18193421 | Jon99Ci | Jonah 99Ci | NM_079831 | 43545 |
| 18137009 | CG33282 | CG33282 | NM_001103597 | 2768939 |
| 18173370 | CR43306 | ncRNA | NR_048294 | 12797933 |
| 18188653 | Gr98a | Gustatory receptor 98a | NM_143307 | 43305 |
| 18164897 | CR43421 | ncRNA | NR_048168 | 12798123 |
| 18143895 | fon | fondue | NM_136115 | 35211 |
| 18182219 | CG42598 | CG42598 | NM_001170386 | 8674042 |
| 18144354 | Peritrophin-15a | CG17814 | NM_080244 | 50433 |
| 18182134 | CG40002 | CG40002 | NM_001015476 | 3355155 |
| 18212788 | Yp2 | Yolk protein 2 | NM_078547 | 31938 |
| 18154395 | Acp54A1 | CG34098 | NM_001043089 | 4379879 |
| 18142708 | CG9525 | CG9525 | NM_135416 | 34217 |
| 18153786 | CG30354 | CG30354 | NM_165616 | 246560 |
| 18216626 | CG1304 | CG1304 | NM_134574 | 33074 |
| 18138288 | CG43055 | CG43055 | NM_001258938 | 12798556 |
| 18171905 | CG33926 | CG33926 | NM_001031947 | 39092 |
| 18140935 | CG15254 | CG15254 | NM_135913 | 34915 |
| 18144052 | CG10659 | CG10659 | NM_136174 | 35285 |
| 18216750 | vanin-like | CG32754 | NM_132069 | 31551 |
| 18192132 | CG43061 | CG43061 | NM_001260053 | 12798058 |
| 18151186 | Gbp | Growth-blocking peptide | NM_137350 | 36936 |
| 18202554 | Dup99B | Ductus ejaculatorius peptide 99B | NM_001260430 | 2768691 |
| 18163737 | CG34236 | CG34236 | NM_001103833 | 5740183 |
| 18189622 | CG18749 | CG18749 | NM_001031983 | 3771984 |
| 18213753 | antdh | CG1386 | NM_132467 | 32058 |
| 18151370 | CG14500 | CG14500 | NM_137465 | 37087 |
| 18165645 | LysE | Lysozyme E | NM_057479 | 38128 |
| 18185920 | CG11598 | CG11598 | NM_001043245 | 41554 |
| 18131665 | kek2 | kekkon-2 | NM_078827 | 34582 |
| 18188796 | CG1894 | CG1894 | NM_143363 | 43378 |
| 18154945 | CG42362 | CG42362 | NM_001144259 | 7354407 |
| 18154949 | CG42362 | CG42362 | NM_001144259 | 7354407 |
| 18134874 | CG17107 | CG17107 | NM_135591 | 34467 |
| 18162601 | CG30039 | CG30039 | NM_165862 | 246411 |
| 18177135 | CG5653 | CG5653 | NM_140012 | 39024 |
| 18198056 | CG7142 | CG7142 | NM_142446 | 42194 |
| 18138528 | CG43401 | CG43401 | NM_001258908 | 12797875 |
| 18137500 | CG34180 | CG34180 | NM_001103626 | 5740541 |
| 18158274 | mthl3 | methuselah-like 3 | NM_145335 | 36961 |
| 18183287 | Hsp70Ab | Heat-shock-protein-70Ab | NM_080059 | 44920 |
| 18154123 | CG33462 | CG33462 | NM_206122 | 2768841 |
| 18190299 | Hsp70Bbb | CG5834 | NM_176486 | 50022 |
| 18166757 | Vha16-3 | Vacuolar H[+] ATPase subunit 16-3 | NM_001202157 | 317846 |
| 18169788 | CG7580 | CG7580 | NM_140728 | 39950 |
| 18199512 | CG10559 | CG10559 | NM_143125 | 43063 |
| 18180949 | CG42255 | CG42255 | NM_168477 | 39334 |
| 18209853 | Sec61gamma | CG14214 | NM_134493 | 32968 |
| 18171092 | Cpr66D | Cuticular protein 66D | NM_168288 | 38990 |
| 18172199 | CG34462 | CG34462 | NM_001104078 | 5740319 |
| 18186079 | CG8141 | CG8141 | NM_169496 | 41622 |
| 18146450 | CG42844 | CG42844 | NM_001201821 | 10178811 |
| 18132956 | CG13947 | CG13947 | NM_134698 | 33250 |
| 18164944 | snoRNA:lola-b | ncRNA | NR_048083 | 12798398 |
| 18214944 | CG15296 | CG15296 | NM_132408 | 31986 |
| 18135013 | CG16965 | CG16965 | NM_135684 | 34607 |
| 18143040 | CG5322 | CG5322 | NM_135563 | 34436 |
| 18219555 |  |  |  |  |
| 18183067 | GstD5 | Glutathione S transferase D5 | NM_080175 | 48338 |
| 18194195 | Hsp70Ba | Heat-shock-protein-70Ba | NM_169469 | 44921 |
| 18197727 | CG10317 | CG10317 | NM_142282 | 41987 |
| 18177484 | CG6168 | CG6168 | NM_140202 | 39273 |
| 18190328 | CG31370 | CG31370 | NM_170216 | 43060 |
| 18141034 | CG11912 | CG11912 | NM_134673 | 33205 |
| 18166760 | Vha16-2 | Vacuolar H[+] ATPase subunit 16-2 | NM_168459 | 39282 |
| 18208317 | CG9689 | CG9689 | NM_132359 | 31922 |
| 18186765 | CG5860 | CG5860 | NM_142365 | 42095 |
| 18160127 | CG6337 | CG6337 | NM_137061 | 36530 |
| 18141318 | Cyp309a1 | CG9964 | NM_134844 | 33438 |
| 18202995 | ymp | yellow-emperor | NM_143147 | 43088 |
| 18169560 | CG13075 | CG13075 | NM_140574 | 39756 |
| 18211864 | CR43297 | ncRNA | NR_047780 | 12797952 |
| 18154314 | Nop60B | Nucleolar protein at 60B | NM_001169818 | 37873 |
| 18212581 | snRNA:U5:14B | small nuclear RNA U5 at 14B | NR_002129 | 3771949 |
| 18163019 | CG30391 | CG30391 | NM_137722 | 37423 |
| 18141540 | CG3513 | CG3513 | NM_134954 | 33590 |
| 18146108 | Sfp24Bb | Seminal fluid protein 24Bb | NM_001169390 | 8674094 |
| 18152409 | CG13540 | CG13540 | NM_137929 | 37686 |
| 18176058 | spz5 | spatzle 5 | NM_139496 | 38350 |
| 18184996 | CG10919 | CG10919 | NM_141476 | 40917 |
| 18154229 | snoRNA:snR38:54Eb | ncRNA | NR_001764 | 3771777 |
| 18146305 | CG42688 | CG42688 | NM_001201914 | 10178824 |
| 18146521 | CR43097 | ncRNA | NR_048013 | 12798019 |
| 18201624 | CG31380 | CG31380 | NM_170242 | 318701 |
| 18136172 | CG12617 | CG12617 | NM_144332 | 50470 |
| 18154607 | CG34423 | CG34423 | NM_001103961 | 5740313 |
| 18152233 | CG4269 | CG4269 | NM_137839 | 37578 |
| 18149513 | Cyp6a13 | CG2397 | NM_136546 | 35837 |
| 18169436 | CG33259 | CG33259 | NM_206368 | 2768950 |
| 18160064 | CG4712 | CG4712 | NM_137012 | 36469 |
| 18186987 | Cyp12a5 | CG11821 | NM_142525 | 42293 |
| 18154348 | snoRNA:Psi28S-1175c | ncRNA | NR_003829 | 5740697 |
| 18154349 |  |  |  |  |
| 18168152 | CG15829 | CG15829 | NM_139825 | 38782 |
| 18138451 | CR43262 | ncRNA | NR_047923 | 12798304 |
| 18209071 | CG15641 | CG15641 | NM_132801 | 32477 |
| 18207682 | CG12729 | CG12729 | NM_132043 | 31515 |
| 18164668 | CG43106 | CG43106 | NM_001259589 | 12797866 |
| 18136839 | Ada1-2 | CG31866 | NM_164980 | 318992 |
| 18164729 | CG43195 | CG43195 | NM_001259509 | 12798124 |
| 18172039 | CG34238 | CG34238 | NM_001104106 | 5740238 |
| 18203186 | CG42824 | CG42824 | NM_001202312 | 10178873 |
| 18190607 | CG33333 | CG33333 | NM_206503 | 2768672 |
| 18186801 | CG14329 | CG14329 | NM_142379 | 42116 |
| 18180564 | CG34025 | CG34025 | NM_001038898 | 3885633 |
| 18185094 | CG11671 | CG11671 | NM_141521 | 40976 |
| 18195572 | CG2663 | CG2663 | NM_141278 | 40649 |
| 18171391 | CG32238 | CG32238 | NM_168100 | 326203 |
| 18200221 | capa | capability | NM_079828 | 43541 |
| 18155829 | CR43399 | ncRNA | NR_048144 | 12798344 |
| 18211858 | CG43288 | CG43288 | NM_001258593 | 12798106 |
| 18191517 | Sfp87B | Seminal fluid protein 87B | NM_001170124 | 8674076 |
| 18193265 | mus308 | mutagen-sensitive 308 | NM_079609 | 41571 |
| 18164936 | snoRNA:2R:9445410 | ncRNA | NR_048104 | 12798444 |
| 18154364 | snoRNA:Psi18S-1347b | ncRNA | NR_003807 | 5740220 |
| 18202247 | CG34279 | CG34279 | NM_001104349 | 5740605 |
| 18136286 | Gr32a | Gustatory receptor 32a | NM_078819 | 34545 |
| 18202078 | CG34034 | CG34034 | NM_001170207 | 3885584 |
| 18205120 | CG40249 | CG40249 | NM_001015114 | 3354883 |
| 18154231 | snoRNA:Me28S-G3277a | ncRNA | NR_001763 | 3772500 |
| 18208684 | CG2543 | CG2543 | NM_132586 | 32212 |
| 18134439 | CG12439 | CG12439 | NM_135430 | 34234 |
| 18142015 | CG9500 | CG9500 | NM_135174 | 33888 |
| 18141236 | CG4259 | CG4259 | NM_134801 | 33385 |
| 18208796 | dmrt11E | doublesex-Mab related 11E | NM_078591 | 32291 |
| 18203550 | CG43441 | CG43441 | NM_001260080 | 12798078 |
| 18143308 | rho-6 | rhomboid-6 | NM_176024 | 34640 |
| 18146105 | Sfp24Ba | Seminal fluid protein 24Ba | NM_001169388 | 8673984 |
| 18202255 | CG34283 | CG34283 | NM_001104361 | 5740817 |
| 18151208 | CG10764 | CG10764 | NM_137369 | 36962 |
| 18183289 | Hsp70Bb | Heat-shock-protein-70Bb | NM_080188 | 48582 |
| 18213037 | CG15865 | CG15865 | NM_132915 | 32641 |
| 18151681 | CG15120 | CG15120 | NM_137584 | 37248 |
| 18144137 | Oseg5 | CG9333 | NM_136220 | 35349 |
| 18154267 | GstE5 | Glutathione S transferase E5 | NM_137483 | 37110 |
| 18179789 | CG32036 | CG32036 | NM_168348 | 39105 |
| 18160078 | CG17048 | CG17048 | NM_137022 | 36479 |
| 18213687 | CG17636 | CG17636 | NM_001258513 | 5740847 |
| 18187053 | CG7342 | CG7342 | NM_001260267 | 42335 |
| 18151379 | CG18536 | CG18536 | NM_137468 | 37093 |
| 18146241 | Sfp24Bc | Seminal fluid protein 24Bc | NM_001169391 | 8673990 |
| 18142139 | CG11322 | CG11322 | NM_135227 | 33949 |
| 18161564 | CG13494 | CG13494 | NM_137777 | 37497 |
| 18148601 | CkIIbeta2 | Casein kinase II beta2 subunit | NM_058059 | 37300 |
| 18192162 | mir-34 | mir-34 stem loop | NR_048362 | 12798087 |
| 18177529 | Muc68Ca | Mucin 68Ca | NM_206332 | 2768980 |
| 18172085 | CG34269 | CG34269 | NM_001103996 | 5740713 |
| 18141305 | CG18641 | CG18641 | NM_134838 | 33429 |
| 18144739 | CG31683 | CG31683 | NM_165330 | 261623 |
| 18155617 | CG43123 | CG43123 | NM_001259253 | 12798080 |
| 18167307 | CG8960 | CG8960 | NM_139441 | 38280 |
| 18167064 | CG9129 | CG9129 | NM_138244 | 38138 |
| 18174590 | can | cannonball | NM_080088 | 45432 |
| 18173244 | CG43146 | CG43146 | NM_001259809 | 12798239 |
| 18162802 | CG30196 | CG30196 | NM_166541 | 37595 |
| 18179568 | CG15024 | CG15024 | NM_144079 | 50172 |
| 18154142 | Vkor | Vitamin-K epoxide reductase | NM_001014533 | 3346188 |
| 18138545 | CR43412 | ncRNA | NR_047960 | 12798233 |
| 18130619 |  |  |  |  |
| 18179592 | CG13465 | CG13465 | NM_080261 | 50282 |
| 18157994 | Tim10 | Translocase of inner membrane 10 | NM_166474 | 37478 |
| 18181104 | CG42536 | CG42536 | NM_001169940 | 8673967 |
| 18183291 | Hsp70Bc | Heat-shock-protein-70Bc | NM_141952 | 48583 |
| 18164232 | CG42559 | CG42559 | NM_001169797 | 246505 |
| 18131155 | snRNA:U3:22A | small nuclear RNA U3 at 22A | NR_001600 | 3772609 |
| 18132226 | Ugt37b1 | UDP-glycosyltransferase 37b1 | NM_080269 | 53584 |
| 18146698 | CR43621 | ncRNA | NR_047928 | 12798311 |
| 18185993 | Cyp304a1 | CG7241 | NM_169484 | 41586 |
| 18157088 | Cyp6a8 | Cytochrome P450-6a8 | NM_079025 | 36666 |
| 18145527 | CheB38b | Chemosensory protein B 38b | NM_206013 | 2768933 |
| 18175717 | CG13898 | CG13898 | NM_138214 | 38092 |
| 18152962 | lectin-46Cb | CG1652 | NM_144375 | 53522 |
| 18217804 | Sdic1 | Sperm-specific dynein intermediate chain 1 | NM_079931 | 43984 |
| 18165426 | Hsp67Bb | Heat shock gene 67Bb | NM_001202148 | 3771872 |
| 18181330 | CG42718 | CG42718 | NM_001202190 | 10178940 |
| 18136867 | CG31909 | CG31909 | NM_001169429 | 319020 |
| 18154227 | snoRNA:snR38:54Ec | ncRNA | NR_001769 | 3772210 |
| 18161305 | CG13430 | CG13430 | NM_001032270 | 3772580 |

**Supplementary Table S1:** 350 transcripts were altered by selective breeding and endurance training in the same direction. Gene names, GenBank and NCBI accession numbers are provided. Fold-changes and direction of change relative to control flies for individual entries can be found in Supplementary Tables S2 and S3.
