# Supplementary material for: Endurance exercise and selective breeding for longevity extend Drosophila healthspan by overlapping mechanisms
Source: Aging (Albany NY). 2015 Aug 8;7(8):535–50. doi: 10.18632/aging.100789 (PMC4586100; doi:10.18632/aging.100789)
Supplement: Supplementary file 3 [file aging-07-535-s003.docx]

**Supplementary Table S2**

| Column ID | gene_  assignment | Gene Symbol | RefSeq | FlyBaseID | p-value | Ratio | Fold-Change |
| --- | --- | --- | --- | --- | --- | --- | --- |
| 18161552 | FBtr0071740 // CG4363 | CG4363 | FBtr0071740 | FBtr0071740 | 2.13E-13 | 2.52696 | 2.52696 |
| 18158990 | NM_136539 // Mal-A7 | Mal-A7 | NM_136539 | FBtr0088757 | 1.16E-12 | 0.0101533 | -98.49 |
| 18178314 | FBtr0075424 // Cpr72E | Cpr72Ec | FBtr0075424 | FBtr0075424 | 1.55E-12 | 35.1381 | 35.1381 |
| 18173516 | NR_048214 // snoRNA: | snoRNA:CG32479-b | NR_048214 | FBtr0309775 | 7.27E-12 | 23.6198 | 23.6198 |
| 18147926 | FBtr0087437 // AttA // | AttA | FBtr0087437 | FBtr0087437 | 1.20E-11 | 0.0189742 | -52.7033 |
| 18149502 | NM_136537 // Mal-A4 | Mal-A4 | NM_136537 | FBtr0088748 | 1.21E-11 | 0.0328135 | -30.4752 |
| 18134388 | FBtr0079684 // CG1309 | CG13091 | FBtr0079684 | FBtr0079684 | 1.37E-11 | 0.484666 | -2.06328 |
| 18132600 | FBtr0080729 // CG1526 | CG15263 | FBtr0080729 | FBtr0080729 | 1.91E-11 | 9.57312 | 9.57312 |
| 18191517 | FBtr0300319 // Sfp87B | Sfp87B | FBtr0300319 | FBtr0300319 | 3.52E-11 | 2.00147 | 2.00147 |
| 18157159 | FBtr0089055 // Cyp9b2 | Cyp9b2 | FBtr0089055 | FBtr0089055 | 5.28E-11 | 1.58352 | 1.58352 |
| 18149978 | FBtr0088213 // CG1293 | CG12934 | FBtr0088213 | FBtr0088213 | 9.09E-11 | 14.5259 | 14.5259 |
| 18156951 | FBtr0072455 // PebII // | PebII | FBtr0072455 | FBtr0072455 | 1.22E-10 | 2.10981 | 2.10981 |
| 18180537 | FBtr0307394 // CG3396 | CG33969 | FBtr0307394 | FBtr0307394 | 1.34E-10 | 0.293632 | -3.40563 |
| 18146471 | FBtr0304142 // CG4287 | CG42876 | FBtr0304142 | FBtr0304142 | 1.56E-10 | 8.06424 | 8.06424 |
| 18199522 | FBtr0084852 // CG1056 | CG10560 | FBtr0084852 | FBtr0084852 | 1.57E-10 | 0.319422 | -3.13066 |
| 18192300 | FBtr0305917 // CG4317 | CG43175 | FBtr0305917 | FBtr0305917 | 1.61E-10 | 13.7957 | 13.7957 |
| 18153188 | FBtr0088709 // PGRP-S | PGRP-SC2 | FBtr0088709 | FBtr0088709 | 1.69E-10 | 2.3513 | 2.3513 |
| 18135984 | NM_078820 // hgo // h | hgo | NM_078820 | FBtr0080201 | 1.92E-10 | 0.259405 | -3.85497 |
| 18188283 | FBtr0084811 // CG1185 | CG11854 | FBtr0084811 | FBtr0084811 | 1.97E-10 | 0.121017 | -8.2633 |
| 18192073 | FBtr0303828 // CG4282 | CG42821 | FBtr0303828 | FBtr0303828 | 2.70E-10 | 3.39964 | 3.39964 |
| 18176684 | FBtr0076963 // eIF4E-4 | eIF4E-4 | FBtr0076963 | FBtr0076963 | 2.71E-10 | 3.06576 | 3.06576 |
| 18151339 | FBtr0089630 // CG1091 | CG10910 | FBtr0089630 | FBtr0089630 | 3.22E-10 | 6.67976 | 6.67976 |
| 18188365 | FBtr0084879 // CG5107 | CG5107 | FBtr0084879 | FBtr0084879 | 4.67E-10 | 2.55816 | 2.55816 |
| 18170188 | FBtr0074843 // Spn77B | Spn77Bb | FBtr0074843 | FBtr0074843 | 5.07E-10 | 1.73835 | 1.73835 |
| 18133827 | FBtr0306841 // CG1103 | CG11034 | FBtr0306841 | FBtr0306841 | 6.22E-10 | 3.8824 | 3.8824 |
| 18163186 | FBtr0089419 // Cyp12d | Cyp12d1-p | FBtr0089419 | FBtr0089419 | 6.50E-10 | 0.517924 | -1.93079 |
| 18218028 | NR_002464 // snoRNA: | snoRNA:Psi28S-3436a | NR_002464 | FBtr0091752 | 6.54E-10 | 2.4004 | 2.4004 |
| 18151405 | NM_137479 // GstE1 // | GstE1 | NM_137479 | FBtr0086669 | 7.13E-10 | 3.43184 | 3.43184 |
| 18198686 | NM_142736 // fit // fe | fit | NM_142736 | FBtr0084153 | 7.58E-10 | 5.33435 | 5.33435 |
| 18152233 | FBtr0071854 // CG4269 | CG4269 | FBtr0071854 | FBtr0071854 | 8.16E-10 | 2.61486 | 2.61486 |
| 18214629 | FBtr0333310 // CG2233 | CG2233 | FBtr0333310 | FBtr0333310 | 8.26E-10 | 1.68805 | 1.68805 |
| 18141537 | FBtr0077550 // CG1670 | CG16704 | FBtr0077550 | FBtr0077550 | 8.71E-10 | 14.3401 | 14.3401 |
| 18156154 | FBtr0086133 // Cyp6a2 | Cyp6a2 | FBtr0086133 | FBtr0086133 | 8.99E-10 | 8.51131 | 8.51131 |
| 18154395 | FBtr0110768 // Acp54A | Acp54A1 | FBtr0110768 | FBtr0110768 | 9.26E-10 | 4.78639 | 4.78639 |
| 18210607 | FBtr0307489 // CG3269 | CG32698 | FBtr0307489 | FBtr0307489 | 9.88E-10 | 1.96403 | 1.96403 |
| 18156279 | FBtr0333557 // Lcp1 // | Lcp1 | FBtr0333557 | FBtr0333557 | 1.18E-09 | 27.1899 | 27.1899 |
| 18147462 | NM_057460 // Dpt // D | Dpt | NM_057460 | FBtr0086620 | 1.27E-09 | 0.551419 | -1.8135 |
| 18202278 | FBtr0112487 // CG3429 | CG34291 | FBtr0112487 | FBtr0112487 | 1.31E-09 | 3.36065 | 3.36065 |
| 18188602 | FBtr0085105 // CG1718 | CG17189 | FBtr0085105 | FBtr0085105 | 1.74E-09 | 0.327115 | -3.05703 |
| 18193421 | FBtr0085511 // Jon99Ci | Jon99Ci | FBtr0085511 | FBtr0085511 | 1.86E-09 | 2.53351 | 2.53351 |
| 18200150 | NM_143456 // Obp99b | Obp99b | NM_143456 | FBtr0085462 | 1.91E-09 | 2.22427 | 2.22427 |
| 18195001 | NM_079589 // Ugt35b | Ugt35b | NM_079589 | FBtr0082375 | 1.91E-09 | 0.392391 | -2.54848 |
| 18171442 | FBtr0073059 // Drsl2 // | Drsl2 | FBtr0073059 | FBtr0073059 | 2.26E-09 | 0.0676459 | -14.7829 |
| 18184257 | FBtr0083971 // TotA // | TotA | FBtr0083971 | FBtr0083971 | 2.46E-09 | 2.32018 | 2.32018 |
| 18158049 | FBtr0088458 // CG1516 | CG1516 | FBtr0088458 | FBtr0088458 | 3.08E-09 | 0.645241 | -1.54981 |
| 18165360 | NM_001274820 // Est- | Est-6 | NM_001274820 | FBtr0333383 | 3.64E-09 | 1.93125 | 1.93125 |
| 18160806 | FBtr0086894 // CG1093 | CG10936 | FBtr0086894 | FBtr0086894 | 3.77E-09 | 4.3134 | 4.3134 |
| 18161522 | FBtr0071682 // pirk // p | pirk | FBtr0071682 | FBtr0071682 | 3.79E-09 | 1.93891 | 1.93891 |
| 18146555 | NR_047871 // CR43263 | CR43263 | NR_047871 | FBtr0306546 | 3.82E-09 | 0.0520367 | -19.2172 |
| 18202078 | FBtr0300407 // CG3403 | CG34034 | FBtr0300407 | FBtr0300407 | 3.99E-09 | 1.79168 | 1.79168 |
| 18196255 | FBtr0082101 // Fst // Fr | Fst | FBtr0082101 | FBtr0082101 | 4.11E-09 | 0.473995 | -2.10973 |
| 18138585 | NR_073764 // CR43051 | CR43051 | NR_073764 | FBtr0334410 | 4.13E-09 | 0.237699 | -4.207 |
| 18216520 | FBtr0308650 // CG1561 | CG15618 | FBtr0308650 | FBtr0308650 | 4.20E-09 | 0.116248 | -8.6023 |
| 18145006 | FBtr0077540 // CG3195 | CG31955 | FBtr0077540 | FBtr0077540 | 4.54E-09 | 3.45873 | 3.45873 |
| 18168685 | FBtr0076387 // CG8329 | CG8329 | FBtr0076387 | FBtr0076387 | 4.60E-09 | 4.38778 | 4.38778 |
| 18144930 | FBtr0080726 // CG3183 | CG31832 | FBtr0080726 | FBtr0080726 | 4.65E-09 | 0.250999 | -3.98409 |
| 18134298 | FBtr0079595 // CG1427 | CG14277 | FBtr0079595 | FBtr0079595 | 4.97E-09 | 2.21484 | 2.21484 |
| 18140731 | FBtr0080618 // nimB2 / | nimB2 | FBtr0080618 | FBtr0080618 | 5.18E-09 | 3.21318 | 3.21318 |
| 18215100 | FBtr0073613 // CG9360 | CG9360 | FBtr0073613 | FBtr0073613 | 5.23E-09 | 0.194374 | -5.14473 |
| 18214734 | FBtr0071240 // CG1211 | CG12116 | FBtr0071240 | FBtr0071240 | 5.51E-09 | 0.568748 | -1.75825 |
| 18172304 | FBtr0076914 // CG8628 | CG8628 | FBtr0076914 | FBtr0076914 | 5.65E-09 | 0.18678 | -5.35389 |
| 18138279 | NR_073764 // CR43051 | CR43051 | NR_073764 | FBtr0334410 | 6.32E-09 | 0.191111 | -5.23256 |
| 18186681 | FBtr0083330 // CG1756 | CG17560 | FBtr0083330 | FBtr0083330 | 6.88E-09 | 0.431442 | -2.31781 |
| 18153861 | FBtr0072016 // CG3041 | CG30411 | FBtr0072016 | FBtr0072016 | 6.96E-09 | 0.307088 | -3.2564 |
| 18182175 | FBtr0305959 // CG1737 | CG17374 | FBtr0305959 | FBtr0305959 | 7.14E-09 | 0.551736 | -1.81246 |
| 18208904 | FBtr0073811 // CG1116 | CG11162 | FBtr0073811 | FBtr0073811 | 7.31E-09 | 0.477098 | -2.096 |
| 18142324 | FBtr0079515 // CG7025 | CG7025 | FBtr0079515 | FBtr0079515 | 7.32E-09 | 0.155207 | -6.44301 |
| 18142869 | FBtr0079891 // CG5846 | CG5846 | FBtr0079891 | FBtr0079891 | 7.63E-09 | 0.178951 | -5.58812 |
| 18216226 | FBtr0074626 // CG1504 | CG15043 | FBtr0074626 | FBtr0074626 | 7.82E-09 | 1.66588 | 1.66588 |
| 18170952 | NM_144453 // Nplp3 // | Nplp3 | NM_144453 | FBtr0075369 | 8.67E-09 | 2.48467 | 2.48467 |
| 18147689 | FBtr0088122 // betaTry | betaTry | FBtr0088122 | FBtr0088122 | 8.86E-09 | 0.351687 | -2.84344 |
| 18153106 | FBtr0305795 // AttC // | AttC | FBtr0305795 | FBtr0305795 | 8.92E-09 | 0.343957 | -2.90734 |
| 18209643 | FBtr0074631 // CG6891 | CG6891 | FBtr0074631 | FBtr0074631 | 1.02E-08 | 2.02638 | 2.02638 |
| 18178712 | FBtr0075091 // Cyp12c | Cyp12c1 | FBtr0075091 | FBtr0075091 | 1.02E-08 | 0.160799 | -6.21895 |
| 18141036 | FBtr0078098 // CG1191 | CG11911 | FBtr0078098 | FBtr0078098 | 1.05E-08 | 0.225761 | -4.42946 |
| 18210765 | FBtr0100081 // CG3402 | CG34026 | FBtr0100081 | FBtr0100081 | 1.06E-08 | 2.22953 | 2.22953 |
| 18216848 | FBtr0070185 // CG3690 | CG3690 | FBtr0070185 | FBtr0070185 | 1.14E-08 | 2.07117 | 2.07117 |
| 18217524 | FBtr0307050 // Muc4B | Muc4B | FBtr0307050 | FBtr0307050 | 1.17E-08 | 0.0751415 | -13.3082 |
| 18166626 | FBtr0073157 // Fie // Fi | Fie | FBtr0073157 | FBtr0073157 | 1.19E-08 | 1.84403 | 1.84403 |
| 18136636 | FBtr0080273 // CG3170 | CG31704 | FBtr0080273 | FBtr0080273 | 1.34E-08 | 2.50734 | 2.50734 |
| 18205607 | FBtr0073651 // Lsp1alp | Lsp1alpha | FBtr0073651 | FBtr0073651 | 1.41E-08 | 6.32631 | 6.32631 |
| 18131170 | NM_057431 // Uro // U | Uro | NM_057431 | FBtr0079486 | 1.47E-08 | 1.86592 | 1.86592 |
| 18174309 | FBtr0072637 // LysX // | LysX | FBtr0072637 | FBtr0072637 | 1.60E-08 | 0.180508 | -5.53991 |
| 18161336 | FBtr0086249 // CG1679 | CG16799 | FBtr0086249 | FBtr0086249 | 1.66E-08 | 1.80941 | 1.80941 |
| 18144661 | FBtr0079207 // CG3163 | CG31639 | FBtr0079207 | FBtr0079207 | 1.73E-08 | 0.433096 | -2.30896 |
| 18162032 | FBtr0072196 // CG3907 | CG3907 | FBtr0072196 | FBtr0072196 | 1.94E-08 | 0.637141 | -1.56951 |
| 18135978 | FBtr0308065 // lectin-2 | lectin-28C | FBtr0308065 | FBtr0308065 | 1.98E-08 | 0.113287 | -8.8271 |
| 18161213 | FBtr0086325 // CG1689 | CG16898 | FBtr0086325 | FBtr0086325 | 2.01E-08 | 3.80385 | 3.80385 |
| 18210519 | FBtr0073716 // CG3264 | CG32643 | FBtr0073716 | FBtr0073716 | 2.01E-08 | 1.756 | 1.756 |
| 18173880 | FBtr0076496 // Hsp26 / | Hsp26 | FBtr0076496 | FBtr0076496 | 2.10E-08 | 2.11956 | 2.11956 |
| 18131750 | FBtr0081201 // Lectin-g | Lectin-galC1 | FBtr0081201 | FBtr0081201 | 2.10E-08 | 2.55567 | 2.55567 |
| 18146108 | FBtr0300297 // Sfp24B | Sfp24Bb | FBtr0300297 | FBtr0300297 | 2.17E-08 | 1.64248 | 1.64248 |
| 18141540 | FBtr0077549 // CG3513 | CG3513 | FBtr0077549 | FBtr0077549 | 2.18E-08 | 2.37967 | 2.37967 |
| 18149383 | FBtr0088923 // CG2064 | CG2064 | FBtr0088923 | FBtr0088923 | 2.31E-08 | 4.01949 | 4.01949 |
| 18154453 | FBtr0112420 // CG3422 | CG34227 | FBtr0112420 | FBtr0112420 | 2.33E-08 | 2.87109 | 2.87109 |
| 18154273 | NM_137480 // GstE2 // | GstE2 | NM_137480 | FBtr0086670 | 2.33E-08 | 2.78734 | 2.78734 |
| 18135428 | FBtr0081068 // CG1017 | CG10178 | FBtr0081068 | FBtr0081068 | 2.50E-08 | 1.6801 | 1.6801 |
| 18182240 | NM_079643 // Act88F / | Act88F | NM_079643 | FBtr0083143 | 2.57E-08 | 2.42543 | 2.42543 |
| 18179653 | NM_079225 // Prat2 // | Prat2 | NM_079225 | FBtr0076945 | 2.65E-08 | 1.76428 | 1.76428 |
| 18131649 | FBtr0301665 // Try29F | Try29F | FBtr0301665 | FBtr0301665 | 2.77E-08 | 0.320033 | -3.12468 |
| 18143664 | FBtr0081004 // CG6639 | CG6639 | FBtr0081004 | FBtr0081004 | 2.91E-08 | 1.54052 | 1.54052 |
| 18131045 | NM_165237 // ninaD // | ninaD | NM_165237 | FBtr0081031 | 2.93E-08 | 0.316009 | -3.16446 |
| 18173514 | NR_048215 // snoRNA: | snoRNA:CG32479-a | NR_048215 | FBtr0309760 | 3.19E-08 | 2.41772 | 2.41772 |
| 18197461 | FBtr0083038 // CG6912 | CG6912 | FBtr0083038 | FBtr0083038 | 3.24E-08 | 3.82355 | 3.82355 |
| 18217333 | FBtr0073474 // CG3266 | CG32667 | FBtr0073474 | FBtr0073474 | 3.30E-08 | 1.54634 | 1.54634 |
| 18180657 | NR_003860 // snoRNA: | snoRNA:Psi28S-1837b | NR_003860 | FBtr0113608 | 3.39E-08 | 2.91405 | 2.91405 |
| 18160937 | NM_137474 // IM23 // | IM23 | NM_137474 | FBtr0086729 | 3.64E-08 | 2.10995 | 2.10995 |
| 18169560 | FBtr0301031 // CG1307 | CG13075 | FBtr0301031 | FBtr0301031 | 3.74E-08 | 0.413556 | -2.41805 |
| 18156749 | FBtr0088160 // epsilon | epsilonTry | FBtr0088160 | FBtr0088160 | 3.76E-08 | 2.56646 | 2.56646 |
| 18168689 | FBtr0076389 // CG1818 | CG18180 | FBtr0076389 | FBtr0076389 | 3.82E-08 | 3.42585 | 3.42585 |
| 18159265 | FBtr0310033 // CG1888 | CG1888 | FBtr0310033 | FBtr0310033 | 4.43E-08 | 0.0771045 | -12.9694 |
| 18162478 | NM_166079 // Obp51a | Obp51a | NM_166079 | FBtr0087420 | 4.96E-08 | 1.56378 | 1.56378 |
| 18208270 | FBtr0071309 // CG1536 | CG15369 | FBtr0071309 | FBtr0071309 | 5.84E-08 | 7.88639 | 7.88639 |
| 18197227 | FBtr0082789 // CG1436 | CG14369 | FBtr0082789 | FBtr0082789 | 6.29E-08 | 0.146608 | -6.82092 |
| 18183287 | FBtr0082482 // Hsp70A | Hsp70Aa | FBtr0082482 | FBtr0082482 | 6.82E-08 | 2.91856 | 2.91856 |
| 18157154 | FBtr0089056 // Cyp9b1 | Cyp9b1 | FBtr0089056 | FBtr0089056 | 6.86E-08 | 0.182262 | -5.48661 |
| 18144307 | FBtr0077539 // lectin-2 | lectin-24A | FBtr0077539 | FBtr0077539 | 7.82E-08 | 4.26344 | 4.26344 |
| 18134568 | FBtr0079864 // CG1311 | CG13117 | FBtr0079864 | FBtr0079864 | 7.87E-08 | 2.12984 | 2.12984 |
| 18175717 | FBtr0072599 // CG1389 | CG13898 | FBtr0072599 | FBtr0072599 | 8.18E-08 | 1.68784 | 1.68784 |
| 18171545 | FBtr0076749 // CG3236 | CG32368 | FBtr0076749 | FBtr0076749 | 8.80E-08 | 2.44354 | 2.44354 |
| 18185920 | FBtr0110781 // CG1159 | CG11598 | FBtr0110781 | FBtr0110781 | 9.34E-08 | 2.22352 | 2.22352 |
| 18148066 | NM_080089 // Cyp4p1 | Cyp4p1 | NM_080089 | FBtr0088592 | 1.04E-07 | 0.660644 | -1.51367 |
| 18154281 | NM_079076 // Act57B | Act57B | NM_079076 | FBtr0071519 | 1.05E-07 | 1.95019 | 1.95019 |
| 18177554 | FBtr0076131 // CG1165 | CG11652 | FBtr0076131 | FBtr0076131 | 1.06E-07 | 3.16377 | 3.16377 |
| 18189622 | FBtr0091720 // CG3372 | CG33722 | FBtr0091720 | FBtr0091720 | 1.06E-07 | 1.75848 | 1.75848 |
| 18168029 | FBtr0077047 // CG6602 | CG6602 | FBtr0077047 | FBtr0077047 | 1.06E-07 | 1.62391 | 1.62391 |
| 18145131 | NM_175946 // NLaz // | NLaz | NM_175946 | FBtr0077942 | 1.07E-07 | 0.60167 | -1.66204 |
| 18151449 | FBtr0086706 // CG5323 | CG5323 | FBtr0086706 | FBtr0086706 | 1.16E-07 | 1.66247 | 1.66247 |
| 18183289 | FBtr0082637 // Hsp70B | Hsp70Bc | FBtr0082637 | FBtr0082637 | 1.20E-07 | 3.46032 | 3.46032 |
| 18189615 | FBtr0303151 // CG1875 | CG18754 | FBtr0303151 | FBtr0303151 | 1.30E-07 | 29.3479 | 29.3479 |
| 18175633 | FBtr0072574 // mthl9 / | mthl9 | FBtr0072574 | FBtr0072574 | 1.33E-07 | 0.27528 | -3.63267 |
| 18136172 | FBtr0081351 // CG1261 | CG12617 | FBtr0081351 | FBtr0081351 | 1.35E-07 | 2.91457 | 2.91457 |
| 18133047 | FBtr0308697 // CG4896 | CG4896 | FBtr0308697 | FBtr0308697 | 1.37E-07 | 1.57328 | 1.57328 |
| 18132911 | NM_080359 // nAcRbet | nAcRbeta-21C | NM_080359 | FBtr0078064 | 1.41E-07 | 2.17097 | 2.17097 |
| 18212963 | NM_078702 // Ser6 // S | Ser6 | NM_078702 | FBtr0077272 | 1.45E-07 | 0.205725 | -4.86085 |
| 18158996 | NM_136545 // Cyp6a1 | Cyp6a14 | NM_136545 |  | 1.49E-07 | 2.83715 | 2.83715 |
| 18161086 | NM_137543 // Jheh3 // | Jheh3 | NM_137543 | FBtr0086622 | 1.53E-07 | 0.549867 | -1.81862 |
| 18143473 | FBtr0080474 // CG9377 | CG9377 | FBtr0080474 | FBtr0080474 | 1.53E-07 | 0.149444 | -6.69149 |
| 18140474 | FBtr0079900 // CG5885 | CG5885 | FBtr0079900 | FBtr0079900 | 1.56E-07 | 0.622392 | -1.6067 |
| 18144513 | NM_001014457 // Obp | Obp22a | NM_001014457 | FBtr0100503 | 1.68E-07 | 3.04388 | 3.04388 |
| 18135842 | FBtr0273404 // Tsp39D | Tsp39D | FBtr0273404 | FBtr0273404 | 1.68E-07 | 1.9284 | 1.9284 |
| 18177484 | FBtr0076182 // CG6168 | CG6168 | FBtr0076182 | FBtr0076182 | 1.69E-07 | 1.79839 | 1.79839 |
| 18185797 | NM_169429 // HisCl1 / | HisCl1 | NM_169429 | FBtr0082470 | 1.72E-07 | 0.282929 | -3.53446 |
| 18187712 | FBtr0084340 // CG1724 | CG17244 | FBtr0084340 | FBtr0084340 | 1.74E-07 | 0.570014 | -1.75434 |
| 18171391 | FBtr0073344 // CG3223 | CG32238 | FBtr0073344 | FBtr0073344 | 1.77E-07 | 1.83988 | 1.83988 |
| 18205966 | FBtr0071419 // Yp1 // Y | Yp1 | FBtr0071419 | FBtr0071419 | 1.77E-07 | 0.24457 | -4.0888 |
| 18149971 | NM_136756 // Elp2 // | Elp2 | NM_136756 | FBtr0088212 | 1.79E-07 | 3.23559 | 3.23559 |
| 18163019 | FBtr0071621 // CG3039 | CG30391 | FBtr0071621 | FBtr0071621 | 1.79E-07 | 1.78357 | 1.78357 |
| 18154271 | NM_137481 // GstE3 // | GstE3 | NM_137481 | FBtr0086671 | 1.81E-07 | 1.80121 | 1.80121 |
| 18142708 | FBtr0332188 // CG9525 | CG9525 | FBtr0332188 | FBtr0332188 | 1.81E-07 | 3.00486 | 3.00486 |
| 18177459 | NM_140194 // Plod // | Plod | NM_140194 | FBtr0076187 | 1.91E-07 | 1.5743 | 1.5743 |
| 18197727 | FBtr0300677 // CG1031 | CG10317 | FBtr0300677 | FBtr0300677 | 1.92E-07 | 1.62285 | 1.62285 |
| 18190299 | FBtr0082636 // Hsp70B | Hsp70Bbb | FBtr0082636 | FBtr0082636 | 1.95E-07 | 4.57203 | 4.57203 |
| 18192125 | NR_048409 // mir-2494 | mir-2494 | NR_048409 | FBtr0304304 | 1.98E-07 | 0.230512 | -4.33817 |
| 18190622 | FBtr0302527 // CG3334 | CG33346 | FBtr0302527 | FBtr0302527 | 1.99E-07 | 1.84656 | 1.84656 |
| 18159541 | FBtr0088211 // Spn47C | Spn47C | FBtr0088211 | FBtr0088211 | 2.01E-07 | 0.560502 | -1.78412 |
| 18181599 | FBtr0304976 // CG4308 | CG43085 | FBtr0304976 | FBtr0304976 | 2.04E-07 | 0.18884 | -5.29549 |
| 18169001 | FBtr0310535 // CG1412 | CG14125 | FBtr0310535 | FBtr0310535 | 2.10E-07 | 0.616735 | -1.62144 |
| 18141290 | FBtr0330667 // CG3597 | CG3597 | FBtr0330667 | FBtr0330667 | 2.16E-07 | 0.342605 | -2.91882 |
| 18140864 | FBtr0080606 // CG1809 | CG18095 | FBtr0080606 | FBtr0080606 | 2.17E-07 | 0.568538 | -1.7589 |
| 18155112 | FBtr0301270 // CG4256 | CG42566 | FBtr0301270 | FBtr0301270 | 2.21E-07 | 2.48871 | 2.48871 |
| 18184925 | FBtr0081637 // CG1943 | CG1943 | FBtr0081637 | FBtr0081637 | 2.24E-07 | 1.551 | 1.551 |
| 18147667 | NM_079016 // Mdr50 / | Mdr50 | NM_079016 | FBtr0087537 | 2.24E-07 | 1.9316 | 1.9316 |
| 18149265 | FBtr0089036 // Tsp42Er | Tsp42Er | FBtr0089036 | FBtr0089036 | 2.27E-07 | 1.92453 | 1.92453 |
| 18174628 | FBtr0334100 // Gem3 / | Gem3 | FBtr0334100 | FBtr0334100 | 2.31E-07 | 1.88054 | 1.88054 |
| 18186819 | FBtr0331346 // CG1432 | CG14322 | FBtr0331346 | FBtr0331346 | 2.46E-07 | 1.98515 | 1.98515 |
| 18179676 | FBtr0077060 // Cpr65A | Cpr65Au | FBtr0077060 | FBtr0077060 | 2.55E-07 | 1.82595 | 1.82595 |
| 18203550 | FBtr0309077 // CG4344 | CG43441 | FBtr0309077 | FBtr0309077 | 2.67E-07 | 3.35263 | 3.35263 |
| 18151370 | FBtr0086752 // CG1450 | CG14500 | FBtr0086752 | FBtr0086752 | 2.71E-07 | 2.82458 | 2.82458 |
| 18137009 | NM_001103597 // CG3 | CG33282 | NM_001103597 | FBtr0113458 | 2.72E-07 | 0.390584 | -2.56027 |
| 18150198 | FBtr0333061 // CG1834 | CG18343 | FBtr0333061 | FBtr0333061 | 3.08E-07 | 0.6382 | -1.56691 |
| 18158457 | FBtr0086005 // CG1121 | CG11211 | FBtr0086005 | FBtr0086005 | 3.18E-07 | 0.101432 | -9.8588 |
| 18208389 | FBtr0073378 // CG1520 | CG15209 | FBtr0073378 | FBtr0073378 | 3.19E-07 | 0.384124 | -2.60333 |
| 18151889 | FBtr0071520 // CG9344 | CG9344 | FBtr0071520 | FBtr0071520 | 3.25E-07 | 0.610688 | -1.6375 |
| 18190815 | NR_003908 // snoRNA: | snoRNA:Psi28S-3405b | NR_003908 | FBtr0113589 | 3.30E-07 | 0.214024 | -4.67237 |
| 18189219 | FBtr0085655 // CG1553 | CG15539 | FBtr0085655 | FBtr0085655 | 3.34E-07 | 0.292642 | -3.41714 |
| 18199455 | FBtr0113285 // CG9996 | CG9996 | FBtr0113285 | FBtr0113285 | 3.41E-07 | 0.54239 | -1.84369 |
| 18164897 | NR_048168 // CR43421 | CR43421 | NR_048168 | FBtr0308772 | 3.41E-07 | 2.68636 | 2.68636 |
| 18164663 | FBtr0304891 // CG4310 | CG43103 | FBtr0304891 | FBtr0304891 | 3.45E-07 | 2.29552 | 2.29552 |
| 18172117 | FBtr0333611 // CG3439 | CG34391 | FBtr0333611 | FBtr0333611 | 3.73E-07 | 2.35449 | 2.35449 |
| 18146450 | FBtr0303929 // CG4284 | CG42844 | FBtr0303929 | FBtr0303929 | 3.74E-07 | 3.97035 | 3.97035 |
| 18176029 | FBtr0072967 // Cyp4d2 | Cyp4d20 | FBtr0072967 | FBtr0072967 | 4.09E-07 | 0.426931 | -2.3423 |
| 18184996 | FBtr0081739 // CG1091 | CG10919 | FBtr0081739 | FBtr0081739 | 4.20E-07 | 1.97696 | 1.97696 |
| 18202299 | FBtr0112499 // CG3430 | CG34303 | FBtr0112499 | FBtr0112499 | 4.25E-07 | 0.57526 | -1.73835 |
| 18194195 | FBtr0082679 // Hsp70B | Hsp70Ba | FBtr0082679 | FBtr0082679 | 4.27E-07 | 4.28435 | 4.28435 |
| 18206366 | NM_078507 // Spat // S | Spat | NM_078507 | FBtr0070913 | 4.60E-07 | 2.02687 | 2.02687 |
| 18152432 | FBtr0072014 // eIF2B-d | eIF2B-delta | FBtr0072014 | FBtr0072014 | 4.68E-07 | 0.396539 | -2.52182 |
| 18208265 | NM_167184 // Obp8a / | Obp8a | NM_167184 | FBtr0071307 | 4.72E-07 | 0.362759 | -2.75665 |
| 18190740 | FBtr0091720 // CG3372 | CG33722 | FBtr0091720 | FBtr0091720 | 4.83E-07 | 1.70297 | 1.70297 |
| 18164714 | FBtr0305907 // CG4317 | CG43172 | FBtr0305907 | FBtr0305907 | 5.04E-07 | 0.615482 | -1.62474 |
| 18164735 | FBtr0305907 // CG4317 | CG43172 | FBtr0305907 | FBtr0305907 | 5.04E-07 | 0.615482 | -1.62474 |
| 18176593 | FBtr0077068 // CG1047 | CG10477 | FBtr0077068 | FBtr0077068 | 5.14E-07 | 1.85072 | 1.85072 |
| 18142632 | FBtr0079701 // Bace // | Bace | FBtr0079701 | FBtr0079701 | 5.15E-07 | 1.83141 | 1.83141 |
| 18144431 | FBtr0077748 // Cyp309 | Cyp309a2 | FBtr0077748 | FBtr0077748 | 5.35E-07 | 0.275118 | -3.6348 |
| 18173370 | NR_048294 // CR43306 | CR43306 | NR_048294 | FBtr0306840 | 5.42E-07 | 2.43848 | 2.43848 |
| 18190597 | NM_176518 // MtnD // | MtnD | NM_176518 | FBtr0308064 | 5.45E-07 | 0.593605 | -1.68462 |
| 18170193 | FBtr0078172 // Spn77B | Spn77Bc | FBtr0078172 | FBtr0078172 | 5.46E-07 | 1.52174 | 1.52174 |
| 18153181 | NM_166394 // Obp57b | Obp57b | NM_166394 | FBtr0086293 | 5.50E-07 | 2.68626 | 2.68626 |
| 18207615 | FBtr0070780 // CG1273 | CG12730 | FBtr0070780 | FBtr0070780 | 5.66E-07 | 1.83672 | 1.83672 |
| 18193043 | NM_058155 // Gld // G | Gld | NM_058155 | FBtr0081596 | 5.82E-07 | 1.7695 | 1.7695 |
| 18213397 | FBtr0070423 // CG1803 | CG18031 | FBtr0070423 | FBtr0070423 | 6.78E-07 | 0.406162 | -2.46207 |
| 18181550 | FBtr0304649 // CG4306 | CG43064 | FBtr0304649 | FBtr0304649 | 6.81E-07 | 1.77272 | 1.77272 |
| 18165424 | FBtr0076454 // Hsp27 / | Hsp27 | FBtr0076454 | FBtr0076454 | 7.08E-07 | 1.7385 | 1.7385 |
| 18187061 | FBtr0273217 // CG1775 | CG17751 | FBtr0273217 | FBtr0273217 | 7.09E-07 | 0.4838 | -2.06697 |
| 18134997 | NM_135678 // Mal-B1 | Mal-B1 | NM_135678 | FBtr0080275 | 7.18E-07 | 0.489095 | -2.04459 |
| 18138545 | NR_047960 // CR43412 | CR43412 | NR_047960 | FBtr0308754 | 7.25E-07 | 2.40669 | 2.40669 |
| 18192535 | NR_048466 // CR43475 | CR43475 | NR_048466 | FBtr0309305 | 7.33E-07 | 0.228203 | -4.38206 |
| 18137487 | FBtr0112368 // CG3417 | CG34177 | FBtr0112368 | FBtr0112368 | 7.35E-07 | 1.57138 | 1.57138 |
| 18187155 | FBtr0083823 // CG4783 | CG4783 | FBtr0083823 | FBtr0083823 | 7.47E-07 | 0.45989 | -2.17443 |
| 18167122 | FBtr0072692 // CG9168 | CG9168 | FBtr0072692 | FBtr0072692 | 7.65E-07 | 2.83378 | 2.83378 |
| 18186079 | FBtr0082734 // CG8141 | CG8141 | FBtr0082734 | FBtr0082734 | 7.69E-07 | 2.29672 | 2.29672 |
| 18156905 | FBtr0072101 // l(2)efl / | l(2)efl | FBtr0072101 | FBtr0072101 | 7.72E-07 | 1.74166 | 1.74166 |
| 18216382 | NM_001103561 // CG8 | CG8028 | NM_001103561 | FBtr0332393 | 7.75E-07 | 0.290054 | -3.44763 |
| 18161878 | NM_079098 // Or59b / | Or59b | NM_079098 | FBtr0072021 | 7.95E-07 | 2.90066 | 2.90066 |
| 18136867 | FBtr0300460 // CG3190 | CG31909 | FBtr0300460 | FBtr0300460 | 8.30E-07 | 0.429982 | -2.32568 |
| 18202554 | NM_001260430 // Dup | Dup99B | NM_001260430 | FBtr0305053 | 8.33E-07 | 2.57095 | 2.57095 |
| 18180949 | NM_168477 // CG4225 | CG42255 | NM_168477 | FBtr0332131 | 8.47E-07 | 0.342816 | -2.91702 |
| 18186750 | FBtr0083405 // CG5246 | CG5246 | FBtr0083405 | FBtr0083405 | 9.01E-07 | 1.87774 | 1.87774 |
| 18215911 | FBtr0074364 // CG9676 | CG9676 | FBtr0074364 | FBtr0074364 | 9.51E-07 | 1.6351 | 1.6351 |
| 18207985 | FBtr0071013 // CG4607 | CG4607 | FBtr0071013 | FBtr0071013 | 9.86E-07 | 0.56528 | -1.76903 |
| 18172044 | FBtr0112436 // CG3424 | CG34242 | FBtr0112436 | FBtr0112436 | 9.88E-07 | 1.79194 | 1.79194 |
| 18176067 | NM_139504 // scramb2 | scramb2 | NM_139504 | FBtr0073005 | 1.04E-06 | 1.72708 | 1.72708 |
| 18171972 | FBtr0100851 // Kaz1-O | Kaz1-ORFB | FBtr0100851 | FBtr0100851 | 1.06E-06 | 1.66901 | 1.66901 |
| 18159997 | FBtr0087796 // CG1332 | CG13324 | FBtr0087796 | FBtr0087796 | 1.09E-06 | 5.77947 | 5.77947 |
| 18209724 | NM_133130 // HP1D3c | HP1D3csd | NM_133130 | FBtr0074670 | 1.11E-06 | 2.32803 | 2.32803 |
| 18188388 | FBtr0084899 // Lgr3 // | Lgr3 | FBtr0084899 | FBtr0084899 | 1.15E-06 | 2.02892 | 2.02892 |
| 18155829 | NR_048144 // CR43399 | CR43399 | NR_048144 | FBtr0308635 | 1.15E-06 | 2.74889 | 2.74889 |
| 18179865 | FBtr0076147 // CG3208 | CG32086 | FBtr0076147 | FBtr0076147 | 1.17E-06 | 1.50746 | 1.50746 |
| 18200943 | NM_001202266 // Pif1 | Pif1A | NM_001202266 | FBtr0332501 | 1.20E-06 | 0.657254 | -1.52148 |
| 18144477 | FBtr0321267 // CG1878 | CG18787 | FBtr0321267 | FBtr0321267 | 1.20E-06 | 0.528443 | -1.89235 |
| 18209071 | FBtr0074035 // CG1564 | CG15641 | FBtr0074035 | FBtr0074035 | 1.22E-06 | 0.498775 | -2.00491 |
| 18187580 | FBtr0084254 // CG7054 | CG7054 | FBtr0084254 | FBtr0084254 | 1.24E-06 | 0.287089 | -3.48325 |
| 18190088 | FBtr0084505 // LSm3 // | LSm3 | FBtr0084505 | FBtr0084505 | 1.25E-06 | 2.23998 | 2.23998 |
| 18185346 | FBtr0082025 // CG1847 | CG18473 | FBtr0082025 | FBtr0082025 | 1.25E-06 | 1.71376 | 1.71376 |
| 18144052 | FBtr0081350 // CG1065 | CG10659 | FBtr0081350 | FBtr0081350 | 1.25E-06 | 1.86688 | 1.86688 |
| 18184424 | FBtr0111222 // CG1092 | CG1092 | FBtr0111222 | FBtr0111222 | 1.25E-06 | 2.65292 | 2.65292 |
| 18188807 | FBtr0085307 // CG9989 | CG9989 | FBtr0085307 | FBtr0085307 | 1.27E-06 | 0.437277 | -2.28688 |
| 18195572 | FBtr0078796 // CG2663 | CG2663 | FBtr0078796 | FBtr0078796 | 1.28E-06 | 2.81058 | 2.81058 |
| 18210813 | FBtr0070644 // GlcAT-I | GlcAT-I | FBtr0070644 | FBtr0070644 | 1.30E-06 | 0.434291 | -2.30261 |
| 18176610 | FBtr0077063 // CG1046 | CG10467 | FBtr0077063 | FBtr0077063 | 1.35E-06 | 0.656963 | -1.52216 |
| 18216879 | FBtr0070252 // eIF4E-7 | eIF4E-7 | FBtr0070252 | FBtr0070252 | 1.44E-06 | 0.363721 | -2.74936 |
| 18160078 | FBtr0087721 // CG1704 | CG17048 | FBtr0087721 | FBtr0087721 | 1.44E-06 | 1.96135 | 1.96135 |
| 18183063 | NM_176479 // GstD3 / | GstD3 | NM_176479 | FBtr0082570 | 1.48E-06 | 2.08521 | 2.08521 |
| 18155614 | FBtr0304945 // CG4311 | CG43114 | FBtr0304945 | FBtr0304945 | 1.51E-06 | 0.44387 | -2.25291 |
| 18167345 | FBtr0072921 // CG1146 | CG1146 | FBtr0072921 | FBtr0072921 | 1.57E-06 | 0.606708 | -1.64824 |
| 18217842 | NR_003729 // snoRNA: | snoRNA:Psi28S-1060 | NR_003729 | FBtr0113560 | 1.57E-06 | 1.52185 | 1.52185 |
| 18151186 | NM_137350 // Gbp // | Gbp | NM_137350 | FBtr0086986 | 1.67E-06 | 2.23875 | 2.23875 |
| 18190328 | FBtr0290055 // CG3137 | CG31370 | FBtr0290055 | FBtr0290055 | 1.69E-06 | 0.492769 | -2.02935 |
| 18161314 | NM_080378 // Mgat1 / | Mgat1 | NM_080378 | FBtr0086259 | 1.71E-06 | 0.656706 | -1.52275 |
| 18185768 | FBtr0082423 // CG1471 | CG14715 | FBtr0082423 | FBtr0082423 | 1.77E-06 | 0.584932 | -1.7096 |
| 18131665 | FBtr0080252 // kek2 // | kek2 | FBtr0080252 | FBtr0080252 | 1.81E-06 | 0.487035 | -2.05324 |
| 18200425 | FBtr0085722 // Npc2h / | Npc2h | FBtr0085722 | FBtr0085722 | 1.83E-06 | 1.50186 | 1.50186 |
| 18215898 | FBtr0074287 // CG9777 | CG9777 | FBtr0074287 | FBtr0074287 | 1.84E-06 | 1.51017 | 1.51017 |
| 18217428 | FBtr0273437 // CG3270 | CG32702 | FBtr0273437 | FBtr0273437 | 1.87E-06 | 0.520958 | -1.91954 |
| 18137060 | FBtr0091498 // lectin-3 | lectin-37Da | FBtr0091498 | FBtr0091498 | 1.87E-06 | 2.49021 | 2.49021 |
| 18198056 | FBtr0083600 // CG7142 | CG7142 | FBtr0083600 | FBtr0083600 | 1.91E-06 | 2.23896 | 2.23896 |
| 18199503 | FBtr0084864 // CG1051 | CG10514 | FBtr0084864 | FBtr0084864 | 2.02E-06 | 1.60769 | 1.60769 |
| 18177135 | FBtr0076559 // CG5653 | CG5653 | FBtr0076559 | FBtr0076559 | 2.04E-06 | 0.332897 | -3.00393 |
| 18201624 | FBtr0084918 // CG3138 | CG31380 | FBtr0084918 | FBtr0084918 | 2.06E-06 | 0.455622 | -2.1948 |
| 18217940 | FBtr0310439 // spri // s | spri | FBtr0310439 | FBtr0310439 | 2.07E-06 | 0.599951 | -1.6668 |
| 18155662 | FBtr0306049 // CG4318 | CG43188 | FBtr0306049 | FBtr0306049 | 2.09E-06 | 0.282044 | -3.54555 |
| 18133747 | NM_135072 // Cyp4ac1 | Cyp4ac1 | NM_135072 | FBtr0079066 | 2.17E-06 | 1.5794 | 1.5794 |
| 18186402 | FBtr0083026 // CG3987 | CG3987 | FBtr0083026 | FBtr0083026 | 2.18E-06 | 1.71478 | 1.71478 |
| 18188796 | FBtr0085290 // CG1894 | CG1894 | FBtr0085290 | FBtr0085290 | 2.18E-06 | 0.290815 | -3.43861 |
| 18172039 | FBtr0112432 // CG3423 | CG34238 | FBtr0112432 | FBtr0112432 | 2.22E-06 | 2.75424 | 2.75424 |
| 18177257 | FBtr0076421 // CG3088 | CG3088 | FBtr0076421 | FBtr0076421 | 2.22E-06 | 1.54072 | 1.54072 |
| 18207981 | FBtr0071009 // CG4593 | CG4593 | FBtr0071009 | FBtr0071009 | 2.23E-06 | 4.94806 | 4.94806 |
| 18156712 | FBtr0088432 // Def // | Def | FBtr0088432 | FBtr0088432 | 2.26E-06 | 4.41065 | 4.41065 |
| 18145744 | FBtr0079266 // Sec61al | Sec61alpha | FBtr0079266 | FBtr0079266 | 2.28E-06 | 0.593438 | -1.6851 |
| 18182134 | FBtr0113773 // CG4047 | CG40472 | FBtr0113773 | FBtr0113773 | 2.34E-06 | 0.496243 | -2.01514 |
| 18214258 | FBtr0070658 // CG1269 | CG12692 | FBtr0070658 | FBtr0070658 | 2.39E-06 | 0.487451 | -2.05149 |
| 18193419 | FBtr0085512 // Jon99Ci | Jon99Cii | FBtr0085512 | FBtr0085512 | 2.40E-06 | 3.06385 | 3.06385 |
| 18145255 | FBtr0080564 // CG3330 | CG33306 | FBtr0080564 | FBtr0080564 | 2.41E-06 | 2.28614 | 2.28614 |
| 18153008 | FBtr0100554 // CG1351 | CG13516 | FBtr0100554 | FBtr0100554 | 2.45E-06 | 1.83577 | 1.83577 |
| 18143158 | FBtr0114455 // Ast-CC | Ast-CC | FBtr0114455 | FBtr0114455 | 2.46E-06 | 2.85265 | 2.85265 |
| 18161676 | FBtr0071845 // babos / | babos | FBtr0071845 | FBtr0071845 | 2.47E-06 | 1.62353 | 1.62353 |
| 18199604 | FBtr0084922 // CG1719 | CG17196 | FBtr0084922 | FBtr0084922 | 2.47E-06 | 1.56387 | 1.56387 |
| 18139434 | NR_002130 // snRNA:U | snRNA:U2:14B | NR_002130 | FBtr0074208 | 2.47E-06 | 1.84055 | 1.84055 |
| 18205954 | NR_002130 // snRNA:U | snRNA:U2:14B | NR_002130 | FBtr0074208 | 2.47E-06 | 1.84055 | 1.84055 |
| 18153190 | FBtr0088707 // PGRP-S | PGRP-SC1b | FBtr0088707 | FBtr0088707 | 2.50E-06 | 5.10352 | 5.10352 |
| 18189186 | FBtr0085644 // CG1553 | CG15534 | FBtr0085644 | FBtr0085644 | 2.53E-06 | 1.76353 | 1.76353 |
| 18199512 | FBtr0289942 // CG1055 | CG10559 | FBtr0289942 | FBtr0289942 | 2.54E-06 | 2.31811 | 2.31811 |
| 18182621 | FBtr0085502 // Jon99Ci | Jon99Cii | FBtr0085502 | FBtr0085502 | 2.55E-06 | 2.60926 | 2.60926 |
| 18133640 | FBtr0332476 // CG1219 | CG12194 | FBtr0332476 | FBtr0332476 | 2.57E-06 | 1.51965 | 1.51965 |
| 18197578 | FBtr0083106 // CG6125 | CG6125 | FBtr0083106 | FBtr0083106 | 2.64E-06 | 0.453394 | -2.20559 |
| 18133145 | FBtr0077816 // Npc2a / | Npc2a | FBtr0077816 | FBtr0077816 | 2.66E-06 | 1.60128 | 1.60128 |
| 18165226 | FBtr0308893 // CG4049 | CG40498 | FBtr0308893 | FBtr0308893 | 2.70E-06 | 1.84248 | 1.84248 |
| 18162754 | FBtr0086266 // CG3014 | CG30148 | FBtr0086266 | FBtr0086266 | 2.75E-06 | 1.65248 | 1.65248 |
| 18137600 | NR_003763 // snoRNA: | snoRNA:Me28S-A3365 | NR_003763 | FBtr0114316 | 2.77E-06 | 2.84352 | 2.84352 |
| 18187628 | FBtr0084269 // CG1384 | CG13843 | FBtr0084269 | FBtr0084269 | 2.77E-06 | 0.604799 | -1.65344 |
| 18146521 | NR_048013 // CR43097 | CR43097 | NR_048013 | FBtr0304883 | 2.77E-06 | 0.470825 | -2.12393 |
| 18187075 | FBtr0083780 // CG1672 | CG16727 | FBtr0083780 | FBtr0083780 | 2.80E-06 | 0.633428 | -1.57871 |
| 18151197 | FBtr0086916 // CG4802 | CG4802 | FBtr0086916 | FBtr0086916 | 2.83E-06 | 1.59558 | 1.59558 |
| 18154314 | FBtr0300861 // Nop60B | Nop60B | FBtr0300861 | FBtr0300861 | 2.84E-06 | 0.329895 | -3.03126 |
| 18144843 | FBtr0077542 // CG3177 | CG31777 | FBtr0077542 | FBtr0077542 | 2.93E-06 | 1.60167 | 1.60167 |
| 18167389 | FBtr0072937 // CG1143 | CG1143 | FBtr0072937 | FBtr0072937 | 2.95E-06 | 0.573327 | -1.74421 |
| 18150004 | FBtr0088226 // RpS15A | RpS15Ab | FBtr0088226 | FBtr0088226 | 2.96E-06 | 2.16967 | 2.16967 |
| 18170639 | FBtr0332052 // CG1113 | CG11131 | FBtr0332052 | FBtr0332052 | 2.96E-06 | 0.284828 | -3.51089 |
| 18134177 | FBtr0079487 // CG7164 | CG7164 | FBtr0079487 | FBtr0079487 | 2.96E-06 | 1.61545 | 1.61545 |
| 18173244 | FBtr0305484 // CG4314 | CG43146 | FBtr0305484 | FBtr0305484 | 2.99E-06 | 0.513724 | -1.94657 |
| 18164668 | FBtr0304889 // CG4310 | CG43106 | FBtr0304889 | FBtr0304889 | 3.01E-06 | 2.25412 | 2.25412 |
| 18176491 | FBtr0077141 // CG1328 | CG13285 | FBtr0077141 | FBtr0077141 | 3.02E-06 | 0.633225 | -1.57922 |
| 18146095 | FBtr0310075 // nimC1 / | nimC1 | FBtr0310075 | FBtr0310075 | 3.10E-06 | 2.01132 | 2.01132 |
| 18214944 | FBtr0071512 // CG1529 | CG15296 | FBtr0071512 | FBtr0071512 | 3.14E-06 | 0.509024 | -1.96454 |
| 18200456 | FBtr0290201 // CG1555 | CG15553 | FBtr0290201 | FBtr0290201 | 3.15E-06 | 0.516334 | -1.93673 |
| 18154267 | NM_137483 // GstE5 // | GstE5 | NM_137483 | FBtr0086673 | 3.19E-06 | 2.27071 | 2.27071 |
| 18149232 | FBtr0089030 // Tsp42Ej | Tsp42Ej | FBtr0089030 | FBtr0089030 | 3.22E-06 | 1.6921 | 1.6921 |
| 18194887 | FBtr0305991 // CREG // | CREG | FBtr0305991 | FBtr0305991 | 3.28E-06 | 0.622308 | -1.60692 |
| 18141318 | FBtr0077749 // Cyp309 | Cyp309a1 | FBtr0077749 | FBtr0077749 | 3.31E-06 | 2.14516 | 2.14516 |
| 18216705 | FBtr0077212 // Cyp6t1 | Cyp6t1 | FBtr0077212 | FBtr0077212 | 3.32E-06 | 1.94488 | 1.94488 |
| 18163698 | FBtr0112408 // CG3421 | CG34215 | FBtr0112408 | FBtr0112408 | 3.36E-06 | 1.63562 | 1.63562 |
| 18216626 | FBtr0077271 // CG1304 | CG1304 | FBtr0077271 | FBtr0077271 | 3.39E-06 | 0.222963 | -4.48504 |
| 18135670 | FBtr0081302 // CG1677 | CG16772 | FBtr0081302 | FBtr0081302 | 3.46E-06 | 0.493474 | -2.02645 |
| 18154607 | FBtr0301854 // CG3442 | CG34423 | FBtr0301854 | FBtr0301854 | 3.66E-06 | 0.375549 | -2.66277 |
| 18141968 | FBtr0302213 // CG9150 | CG9150 | FBtr0302213 | FBtr0302213 | 3.77E-06 | 1.88387 | 1.88387 |
| 18216750 | FBtr0070879 // vanin-li | vanin-like | FBtr0070879 | FBtr0070879 | 3.80E-06 | 0.466582 | -2.14325 |
| 18162258 | FBtr0072411 // Tina-1 / | Tina-1 | FBtr0072411 | FBtr0072411 | 3.85E-06 | 0.647618 | -1.54412 |
| 18185928 | FBtr0303841 // CG1160 | CG11608 | FBtr0303841 | FBtr0303841 | 3.87E-06 | 0.566145 | -1.76633 |
| 18168687 | FBtr0076388 // CG1817 | CG18179 | FBtr0076388 | FBtr0076388 | 3.87E-06 | 0.253511 | -3.9446 |
| 18153511 | FBtr0086215 // CG3015 | CG30154 | FBtr0086215 | FBtr0086215 | 3.88E-06 | 0.348341 | -2.87075 |
| 18168879 | FBtr0076173 // CG7560 | CG7560 | FBtr0076173 | FBtr0076173 | 4.04E-06 | 1.79483 | 1.79483 |
| 18154251 | NR_001756 // snoRNA: | snoRNA:Me28S-A3407a | NR_001756 | FBtr0086844 | 4.05E-06 | 0.590961 | -1.69216 |
| 18187611 | NM_001170219 // oa2 | oa2 | NM_001170219 | FBtr0301484 | 4.17E-06 | 1.7848 | 1.7848 |
| 18162334 | NM_144038 // IM4 // I | IM4 | NM_144038 | FBtr0071559 | 4.20E-06 | 0.604228 | -1.655 |
| 18171092 | FBtr0076567 // Cpr66D | Cpr66D | FBtr0076567 | FBtr0076567 | 4.21E-06 | 2.00914 | 2.00914 |
| 18187205 | FBtr0083884 // MtnC // | MtnC | FBtr0083884 | FBtr0083884 | 4.35E-06 | 1.67606 | 1.67606 |
| 18165422 | FBtr0076453 // Hsp23 / | Hsp23 | FBtr0076453 | FBtr0076453 | 4.50E-06 | 1.66409 | 1.66409 |
| 18207604 | FBtr0070777 // CG1578 | CG15784 | FBtr0070777 | FBtr0070777 | 4.58E-06 | 0.462309 | -2.16306 |
| 18215117 | FBtr0073604 // p24-1 / | p24-1 | FBtr0073604 | FBtr0073604 | 4.59E-06 | 1.60984 | 1.60984 |
| 18167954 | FBtr0077086 // CG5150 | CG5150 | FBtr0077086 | FBtr0077086 | 4.66E-06 | 2.72586 | 2.72586 |
| 18140870 | FBtr0080685 // CG1527 | CG15279 | FBtr0080685 | FBtr0080685 | 4.69E-06 | 0.595515 | -1.67922 |
| 18138773 | NR_073787 // CR43825 | CR43825 | NR_073787 | FBtr0332227 | 4.69E-06 | 3.0238 | 3.0238 |
| 18155109 | FBtr0301269 // CG4256 | CG42565 | FBtr0301269 | FBtr0301269 | 4.73E-06 | 1.94393 | 1.94393 |
| 18200147 | NM_143454 // Obp99c | Obp99c | NM_143454 | FBtr0085463 | 4.79E-06 | 1.51697 | 1.51697 |
| 18135799 | FBtr0332034 // CG9259 | CG9259 | FBtr0332034 | FBtr0332034 | 4.82E-06 | 0.615802 | -1.6239 |
| 18171345 | FBtr0075125 // CG3219 | CG32195 | FBtr0075125 | FBtr0075125 | 5.05E-06 | 0.560124 | -1.78532 |
| 18203391 | FBtr0305707 // CG4316 | CG43166 | FBtr0305707 | FBtr0305707 | 5.07E-06 | 0.170899 | -5.8514 |
| 18164052 | FBtr0299764 // CG4232 | CG42321 | FBtr0299764 | FBtr0299764 | 5.07E-06 | 0.647889 | -1.54347 |
| 18164944 | NR_048083 // snoRNA:l | snoRNA:lola-b | NR_048083 | FBtr0309780 | 5.21E-06 | 1.85559 | 1.85559 |
| 18150304 | FBtr0087922 // Cpr49A | Cpr49Ag | FBtr0087922 | FBtr0087922 | 5.24E-06 | 3.61222 | 3.61222 |
| 18142949 | FBtr0080073 // Hand // | Hand | FBtr0080073 | FBtr0080073 | 5.28E-06 | 0.527455 | -1.8959 |
| 18190376 | FBtr0084830 // CG3143 | CG31436 | FBtr0084830 | FBtr0084830 | 5.64E-06 | 0.601763 | -1.66178 |
| 18185067 | FBtr0113204 // CG7900 | CG7900 | FBtr0113204 | FBtr0113204 | 5.65E-06 | 0.622603 | -1.60616 |
| 18133508 | FBtr0300299 // CG2816 | CG2816 | FBtr0300299 | FBtr0300299 | 5.80E-06 | 1.97382 | 1.97382 |
| 18163021 | FBtr0071618 // CG3039 | CG30392 | FBtr0071618 | FBtr0071618 | 5.90E-06 | 0.397917 | -2.51309 |
| 18140214 | FBtr0100432 // Jon25Bi | Jon25Bi | FBtr0100432 | FBtr0100432 | 5.92E-06 | 2.09583 | 2.09583 |
| 18185485 | FBtr0113212 // CG1294 | CG12947 | FBtr0113212 | FBtr0113212 | 5.93E-06 | 0.566483 | -1.76528 |
| 18152257 | FBtr0309802 // CG4250 | CG4250 | FBtr0309802 | FBtr0309802 | 5.95E-06 | 1.76784 | 1.76784 |
| 18176426 | FBtr0073362 // CG7509 | CG7509 | FBtr0073362 | FBtr0073362 | 6.26E-06 | 1.61807 | 1.61807 |
| 18208442 | FBtr0100644 // Vago // | Vago | FBtr0100644 | FBtr0100644 | 6.30E-06 | 1.72257 | 1.72257 |
| 18190346 | FBtr0273369 // Npc2e / | Npc2e | FBtr0273369 | FBtr0273369 | 6.39E-06 | 0.522922 | -1.91233 |
| 18180485 | FBtr0089526 // CG3349 | CG33490 | FBtr0089526 | FBtr0089526 | 6.56E-06 | 2.02083 | 2.02083 |
| 18172227 | NR_002472 // snoRNA: | snoRNA:Psi28S-2622 | NR_002472 | FBtr0091635 | 6.80E-06 | 1.52015 | 1.52015 |
| 18144493 | FBtr0332971 // CG1885 | CG18858 | FBtr0332971 | FBtr0332971 | 6.90E-06 | 0.514408 | -1.94398 |
| 18150644 | FBtr0087547 // Arc2 // | Arc2 | FBtr0087547 | FBtr0087547 | 6.91E-06 | 1.75992 | 1.75992 |
| 18196234 | FBtr0082111 // CG9396 | CG9396 | FBtr0082111 | FBtr0082111 | 7.07E-06 | 0.643368 | -1.55432 |
| 18187053 | FBtr0304745 // CG7342 | CG7342 | FBtr0304745 | FBtr0304745 | 7.08E-06 | 0.485959 | -2.05779 |
| 18142141 | FBtr0331216 // CG1132 | CG11321 | FBtr0331216 | FBtr0331216 | 7.18E-06 | 0.556245 | -1.79777 |
| 18146105 | FBtr0300296 // Sfp24B | Sfp24Ba | FBtr0300296 | FBtr0300296 | 7.36E-06 | 2.34593 | 2.34593 |
| 18154348 | NR_003829 // snoRNA: | snoRNA:Psi28S-1175c | NR_003829 | FBtr0113529 | 7.46E-06 | 1.71866 | 1.71866 |
| 18216699 | FBtr0077219 // CG1678 | CG1678 | FBtr0077219 | FBtr0077219 | 7.88E-06 | 0.593091 | -1.68608 |
| 18207870 | NM_132101 // Apc7 // | Apc7 | NM_132101 | FBtr0070946 | 7.89E-06 | 0.38009 | -2.63096 |
| 18154245 | NR_001761 // snoRNA: | snoRNA:U29:54Ed | NR_001761 | FBtr0086849 | 7.91E-06 | 0.609862 | -1.63971 |
| 18186197 | FBtr0082826 // Cht5 // | Cht5 | FBtr0082826 | FBtr0082826 | 8.14E-06 | 1.60956 | 1.60956 |
| 18191138 | FBtr0082181 // Invadol | Invadolysin | FBtr0082181 | FBtr0082181 | 8.51E-06 | 0.553862 | -1.8055 |
| 18141034 | FBtr0078099 // CG1191 | CG11912 | FBtr0078099 | FBtr0078099 | 8.52E-06 | 0.530487 | -1.88506 |
| 18208761 | FBtr0073721 // CG2200 | CG2200 | FBtr0073721 | FBtr0073721 | 8.63E-06 | 1.69323 | 1.69323 |
| 18153198 | NR_001754 // Uhg1 // | Uhg1 | NR_001754 | FBtr0086842 | 8.77E-06 | 0.42229 | -2.36804 |
| 18189725 | FBtr0083972 // TotC // | TotC | FBtr0083972 | FBtr0083972 | 9.14E-06 | 2.86876 | 2.86876 |
| 18133081 | FBtr0077920 // CG5397 | CG5397 | FBtr0077920 | FBtr0077920 | 9.18E-06 | 1.78569 | 1.78569 |
| 18200433 | FBtr0085718 // CG1554 | CG15546 | FBtr0085718 | FBtr0085718 | 9.20E-06 | 0.419166 | -2.38569 |
| 18139452 | NR_001645 // snRNA:U | snRNA:U2:34ABa | NR_001645 | FBtr0080486 | 9.33E-06 | 1.68078 | 1.68078 |
| 18169211 | FBtr0075810 // CG1074 | CG10741 | FBtr0075810 | FBtr0075810 | 9.35E-06 | 0.376764 | -2.65418 |
| 18193673 | FBtr0083585 // gl // gla | gl | FBtr0083585 | FBtr0083585 | 9.46E-06 | 0.570428 | -1.75307 |
| 18177597 | FBtr0076084 // CG1036 | CG10361 | FBtr0076084 | FBtr0076084 | 9.51E-06 | 1.70041 | 1.70041 |
| 18202247 | FBtr0112474 // CG3427 | CG34279 | FBtr0112474 | FBtr0112474 | 9.52E-06 | 2.22308 | 2.22308 |
| 18138288 | FBtr0304637 // CG4305 | CG43055 | FBtr0304637 | FBtr0304637 | 9.72E-06 | 0.28884 | -3.46212 |
| 18146241 | FBtr0301934 // Sfp24Bc | Sfp24Bc | FBtr0301934 | FBtr0301934 | 9.77E-06 | 3.02267 | 3.02267 |
| 18180560 | FBtr0100067 // CG3401 | CG34012 | FBtr0100067 | FBtr0100067 | 1.02E-05 | 0.589392 | -1.69666 |
| 18179789 | FBtr0114525 // CG3203 | CG32036 | FBtr0114525 | FBtr0114525 | 1.03E-05 | 2.21176 | 2.21176 |
| 18187099 | FBtr0083791 // CG1145 | CG11453 | FBtr0083791 | FBtr0083791 | 1.03E-05 | 0.654624 | -1.52759 |
| 18157091 | NM_133166 // imd // i | imd | NM_133166 | FBtr0086725 | 1.06E-05 | 0.664875 | -1.50404 |
| 18151379 | FBtr0086758 // CG1853 | CG18536 | FBtr0086758 | FBtr0086758 | 1.07E-05 | 2.07795 | 2.07795 |
| 18212519 | FBtr0073461 // sisA // | sisA | FBtr0073461 | FBtr0073461 | 1.08E-05 | 2.3579 | 2.3579 |
| 18197416 | FBtr0083017 // smp-30 | smp-30 | FBtr0083017 | FBtr0083017 | 1.09E-05 | 1.52788 | 1.52788 |
| 18190561 | FBtr0100204 // CG3309 | CG33095 | FBtr0100204 | FBtr0100204 | 1.10E-05 | 0.481173 | -2.07826 |
| 18149747 | FBtr0088479 // CG1667 | CG1667 | FBtr0088479 | FBtr0088479 | 1.11E-05 | 1.72646 | 1.72646 |
| 18143895 | FBtr0081222 // fon // f | fon | FBtr0081222 | FBtr0081222 | 1.13E-05 | 0.363028 | -2.75461 |
| 18181835 | NR_048276 // CR43432 | CR43432 | NR_048276 | FBtr0309065 | 1.15E-05 | 1.86636 | 1.86636 |
| 18141419 | FBtr0077670 // alpha4 | alpha4GT1 | FBtr0077670 | FBtr0077670 | 1.20E-05 | 1.83157 | 1.83157 |
| 18152276 | FBtr0071878 // CG1352 | CG13526 | FBtr0071878 | FBtr0071878 | 1.20E-05 | 1.57756 | 1.57756 |
| 18180064 | FBtr0073322 // CG3224 | CG32243 | FBtr0073322 | FBtr0073322 | 1.22E-05 | 1.52275 | 1.52275 |
| 18197421 | FBtr0331700 // CG7362 | CG7362 | FBtr0331700 | FBtr0331700 | 1.24E-05 | 0.548534 | -1.82304 |
| 18138661 | NR_073723 // CR43713 | CR43713 | NR_073723 | FBtr0329835 | 1.27E-05 | 2.23807 | 2.23807 |
| 18169668 | FBtr0075388 // Grp // | Grp | FBtr0075388 | FBtr0075388 | 1.27E-05 | 1.63474 | 1.63474 |
| 18201360 | FBtr0084154 // CG3117 | CG31174 | FBtr0084154 | FBtr0084154 | 1.28E-05 | 1.69601 | 1.69601 |
| 18163701 | FBtr0112409 // CG3421 | CG34216 | FBtr0112409 | FBtr0112409 | 1.28E-05 | 2.65831 | 2.65831 |
| 18216707 | FBtr0077369 // CG1461 | CG14615 | FBtr0077369 | FBtr0077369 | 1.28E-05 | 0.44621 | -2.2411 |
| 18174160 | NM_057353 // Rh4 // R | Rh4 | NM_057353 | FBtr0075338 | 1.31E-05 | 1.62348 | 1.62348 |
| 18178676 | FBtr0075117 // CG4306 | CG4306 | FBtr0075117 | FBtr0075117 | 1.33E-05 | 0.558547 | -1.79036 |
| 18192076 | FBtr0303829 // CG4282 | CG42822 | FBtr0303829 | FBtr0303829 | 1.34E-05 | 1.59648 | 1.59648 |
| 18207682 | FBtr0070819 // CG1272 | CG12729 | FBtr0070819 | FBtr0070819 | 1.39E-05 | 0.593018 | -1.68629 |
| 18205969 | FBtr0073821 // Yp3 // Y | Yp3 | FBtr0073821 | FBtr0073821 | 1.40E-05 | 0.40367 | -2.47727 |
| 18176544 | FBtr0077121 // CG1057 | CG10576 | FBtr0077121 | FBtr0077121 | 1.41E-05 | 0.557877 | -1.79251 |
| 18198450 | FBtr0083894 // CG4335 | CG4335 | FBtr0083894 | FBtr0083894 | 1.46E-05 | 2.07415 | 2.07415 |
| 18135414 | FBtr0081049 // CG5783 | CG5783 | FBtr0081049 | FBtr0081049 | 1.48E-05 | 1.75659 | 1.75659 |
| 18202102 | FBtr0083346 // CG3127 | CG31275 | FBtr0083346 | FBtr0083346 | 1.50E-05 | 0.570355 | -1.75329 |
| 18131971 | NM_143795 // Reph // | Reph | NM_143795 | FBtr0077497 | 1.52E-05 | 0.658407 | -1.51882 |
| 18151514 | NM_079063 // DptB // | DptB | NM_079063 | FBtr0086621 | 1.57E-05 | 0.538593 | -1.85669 |
| 18206881 | FBtr0077242 // Mgstl / | Mgstl | FBtr0077242 | FBtr0077242 | 1.58E-05 | 0.577221 | -1.73244 |
| 18196830 | FBtr0306697 // CG1222 | CG12224 | FBtr0306697 | FBtr0306697 | 1.60E-05 | 0.661787 | -1.51106 |
| 18146744 | NM_001273008 // CG4 | CG43750 | NM_001273008 | FBtr0330686 | 1.62E-05 | 2.10803 | 2.10803 |
| 18153548 | FBtr0071967 // CG3019 | CG30192 | FBtr0071967 | FBtr0071967 | 1.64E-05 | 1.54282 | 1.54282 |
| 18177323 | FBtr0113156 // CG6709 | CG6709 | FBtr0113156 | FBtr0113156 | 1.68E-05 | 1.5104 | 1.5104 |
| 18177330 | FBtr0113156 // CG6709 | CG6709 | FBtr0113156 | FBtr0113156 | 1.68E-05 | 1.5104 | 1.5104 |
| 18146244 | FBtr0333233 // Sfp26A | Sfp26Ad | FBtr0333233 | FBtr0333233 | 1.69E-05 | 1.84137 | 1.84137 |
| 18180378 | FBtr0072518 // CG3284 | CG32845 | FBtr0072518 | FBtr0072518 | 1.74E-05 | 0.563423 | -1.77486 |
| 18200657 | FBtr0113321 // Ugt86D | Ugt86Dd | FBtr0113321 | FBtr0113321 | 1.75E-05 | 0.541519 | -1.84666 |
| 18162604 | NM_136968 // Obp49a | Obp49a | NM_136968 | FBtr0087872 | 1.76E-05 | 1.58759 | 1.58759 |
| 18176026 | FBtr0072968 // CG1676 | CG16762 | FBtr0072968 | FBtr0072968 | 1.77E-05 | 0.615406 | -1.62494 |
| 18152248 | FBtr0309799 // CG1351 | CG13510 | FBtr0309799 | FBtr0309799 | 1.78E-05 | 0.64014 | -1.56216 |
| 18155451 | FBtr0303283 // CG4275 | CG42753 | FBtr0303283 | FBtr0303283 | 1.82E-05 | 1.52058 | 1.52058 |
| 18175762 | FBtr0072635 // CG9119 | CG9119 | FBtr0072635 | FBtr0072635 | 1.89E-05 | 0.615795 | -1.62392 |
| 18142600 | FBtr0307092 // CG7627 | CG7627 | FBtr0307092 | FBtr0307092 | 1.97E-05 | 0.633081 | -1.57958 |
| 18176849 | FBtr0076866 // CG1483 | CG14835 | FBtr0076866 | FBtr0076866 | 1.97E-05 | 1.51413 | 1.51413 |
| 18150444 | FBtr0087792 // CG1332 | CG13321 | FBtr0087792 | FBtr0087792 | 1.98E-05 | 0.53165 | -1.88094 |
| 18144601 | FBtr0307507 // CG3160 | CG31601 | FBtr0307507 | FBtr0307507 | 2.00E-05 | 1.57916 | 1.57916 |
| 18188471 | FBtr0273389 // CG1455 | CG14556 | FBtr0273389 | FBtr0273389 | 2.02E-05 | 0.662128 | -1.51028 |
| 18167774 | NM_001259694 // mRp | mRpS6 | NM_001259694 | FBtr0306093 | 2.04E-05 | 1.52475 | 1.52475 |
| 18180100 | FBtr0073142 // CG3226 | CG32267 | FBtr0073142 | FBtr0073142 | 2.04E-05 | 0.633948 | -1.57742 |
| 18133368 | FBtr0077642 // CG3165 | CG3165 | FBtr0077642 | FBtr0077642 | 2.08E-05 | 0.496736 | -2.01314 |
| 18176519 | FBtr0077129 // CG1059 | CG10592 | FBtr0077129 | FBtr0077129 | 2.11E-05 | 2.52646 | 2.52646 |
| 18165645 | FBtr0072630 // LysE // | LysE | FBtr0072630 | FBtr0072630 | 2.15E-05 | 0.40835 | -2.44888 |
| 18189936 | FBtr0084941 // CG3109 | CG31091 | FBtr0084941 | FBtr0084941 | 2.16E-05 | 2.06319 | 2.06319 |
| 18164729 | FBtr0306044 // CG4319 | CG43195 | FBtr0306044 | FBtr0306044 | 2.17E-05 | 2.46975 | 2.46975 |
| 18164865 | FBtr0088984 // Coop // | Coop | FBtr0088984 | FBtr0088984 | 2.19E-05 | 0.642228 | -1.55708 |
| 18160051 | FBtr0087764 // CG4646 | CG4646 | FBtr0087764 | FBtr0087764 | 2.23E-05 | 1.525 | 1.525 |
| 18201661 | FBtr0332431 // CG3144 | CG31445 | FBtr0332431 | FBtr0332431 | 2.28E-05 | 1.5208 | 1.5208 |
| 18141305 | FBtr0307079 // CG1864 | CG18641 | FBtr0307079 | FBtr0307079 | 2.34E-05 | 0.603957 | -1.65575 |
| 18208608 | FBtr0073569 // CG1573 | CG15739 | FBtr0073569 | FBtr0073569 | 2.34E-05 | 0.642395 | -1.55668 |
| 18207162 | FBtr0331714 // CG1109 | CG11092 | FBtr0331714 | FBtr0331714 | 2.36E-05 | 0.553949 | -1.80522 |
| 18157969 | FBtr0089050 // Ady43A | Ady43A | FBtr0089050 | FBtr0089050 | 2.36E-05 | 0.557537 | -1.7936 |
| 18213753 | FBtr0073468 // antdh / | antdh | FBtr0073468 | FBtr0073468 | 2.37E-05 | 0.438006 | -2.28307 |
| 18209145 | FBtr0074088 // CG8260 | CG8260 | FBtr0074088 | FBtr0074088 | 2.37E-05 | 0.631322 | -1.58398 |
| 18145237 | FBtr0302519 // CG3329 | CG33296 | FBtr0302519 | FBtr0302519 | 2.39E-05 | 0.57321 | -1.74456 |
| 18131197 | NR_001646 // snRNA:U | snRNA:U2:34ABb | NR_001646 | FBtr0080443 | 2.40E-05 | 1.65254 | 1.65254 |
| 18144444 | NR_001646 // snRNA:U | snRNA:U2:34ABb | NR_001646 | FBtr0080443 | 2.40E-05 | 1.65254 | 1.65254 |
| 18164603 | NR_048095 // mir-281- | mir-281-1 | NR_048095 | FBtr0304285 | 2.44E-05 | 0.645728 | -1.54864 |
| 18177756 | FBtr0089322 // CG1063 | CG10638 | FBtr0089322 | FBtr0089322 | 2.47E-05 | 1.83436 | 1.83436 |
| 18186987 | NM_142525 // Cyp12a | Cyp12a5 | NM_142525 | FBtr0083730 | 2.47E-05 | 0.471449 | -2.12112 |
| 18143697 | FBtr0080989 // CG6870 | CG6870 | FBtr0080989 | FBtr0080989 | 2.48E-05 | 1.50442 | 1.50442 |
| 18134080 | FBtr0079439 // CG5171 | CG5171 | FBtr0079439 | FBtr0079439 | 2.53E-05 | 0.617183 | -1.62026 |
| 18185094 | FBtr0081829 // CG1167 | CG11671 | FBtr0081829 | FBtr0081829 | 2.61E-05 | 2.22857 | 2.22857 |
| 18199618 | NM_206574 // CcapR / | CcapR | NM_206574 | FBtr0304043 | 2.63E-05 | 3.39697 | 3.39697 |
| 18152096 | FBtr0071726 // CG9304 | CG9304 | FBtr0071726 | FBtr0071726 | 2.63E-05 | 0.572066 | -1.74805 |
| 18188653 | NM_143307 // Gr98a // | Gr98a | NM_143307 | FBtr0085184 | 2.70E-05 | 3.04107 | 3.04107 |
| 18175713 | FBtr0072601 // CG1389 | CG13897 | FBtr0072601 | FBtr0072601 | 2.79E-05 | 0.475836 | -2.10157 |
| 18140949 | FBtr0079624 // Spn28F | Spn28F | FBtr0079624 | FBtr0079624 | 2.82E-05 | 0.515209 | -1.94096 |
| 18190811 | NR_003911 // snoRNA: | snoRNA:Psi28S-3405d | NR_003911 | FBtr0113592 | 2.85E-05 | 1.50296 | 1.50296 |
| 18186801 | FBtr0083463 // CG1432 | CG14329 | FBtr0083463 | FBtr0083463 | 2.92E-05 | 2.94645 | 2.94645 |
| 18150431 | FBtr0087789 // sug // s | sug | FBtr0087789 | FBtr0087789 | 2.97E-05 | 0.640741 | -1.56069 |
| 18187568 | FBtr0084225 // CG1385 | CG13855 | FBtr0084225 | FBtr0084225 | 3.00E-05 | 0.651166 | -1.53571 |
| 18133091 | FBtr0077876 // CG1435 | CG14352 | FBtr0077876 | FBtr0077876 | 3.12E-05 | 1.90912 | 1.90912 |
| 18168806 | FBtr0076193 // Cpr67F | Cpr67Fb | FBtr0076193 | FBtr0076193 | 3.16E-05 | 0.61785 | -1.61852 |
| 18217989 | NR_003732 // snoRNA: | snoRNA:Me28S-U1688 | NR_003732 | FBtr0114320 | 3.23E-05 | 1.82296 | 1.82296 |
| 18138552 | NR_047990 // CR43420 | CR43420 | NR_047990 | FBtr0308769 | 3.30E-05 | 2.73589 | 2.73589 |
| 18157486 | FBtr0087117 // Amyrel | Amyrel | FBtr0087117 | FBtr0087117 | 3.31E-05 | 0.36907 | -2.70951 |
| 18135013 | FBtr0310149 // CG1696 | CG16965 | FBtr0310149 | FBtr0310149 | 3.31E-05 | 2.11544 | 2.11544 |
| 18165758 | NR_003122 // Eip63F-2 | Eip63F-2 | NR_003122 | FBtr0073162 | 3.42E-05 | 1.92494 | 1.92494 |
| 18198987 | FBtr0084431 // CG4408 | CG4408 | FBtr0084431 | FBtr0084431 | 3.51E-05 | 2.02005 | 2.02005 |
| 18182071 | NR_073914 // CR43882 | CR43882 | NR_073914 | FBtr0332743 | 3.52E-05 | 0.254436 | -3.93026 |
| 18188346 | FBtr0273265 // CG1191 | CG11913 | FBtr0273265 | FBtr0273265 | 3.53E-05 | 1.70714 | 1.70714 |
| 18171337 | FBtr0075174 // edin // | edin | FBtr0075174 | FBtr0075174 | 3.54E-05 | 0.433724 | -2.30562 |
| 18190391 | FBtr0334026 // CG3144 | CG31446 | FBtr0334026 | FBtr0334026 | 3.57E-05 | 0.421534 | -2.37229 |
| 18189680 | FBtr0084535 // CG5986 | CG5986 | FBtr0084535 | FBtr0084535 | 3.61E-05 | 1.54201 | 1.54201 |
| 18168592 | FBtr0076444 // CG4447 | CG4447 | FBtr0076444 | FBtr0076444 | 3.62E-05 | 1.89151 | 1.89151 |
| 18208315 | FBtr0071389 // CG9686 | CG9686 | FBtr0071389 | FBtr0071389 | 3.65E-05 | 1.69228 | 1.69228 |
| 18196736 | FBtr0082438 // CG6834 | CG6834 | FBtr0082438 | FBtr0082438 | 3.66E-05 | 1.5215 | 1.5215 |
| 18186132 | FBtr0082756 // CG1437 | CG14377 | FBtr0082756 | FBtr0082756 | 3.73E-05 | 1.59666 | 1.59666 |
| 18138451 | NR_047923 // CR43262 | CR43262 | NR_047923 | FBtr0306547 | 3.82E-05 | 1.9621 | 1.9621 |
| 18151163 | FBtr0300277 // CG6967 | CG6967 | FBtr0300277 | FBtr0300277 | 3.93E-05 | 2.42096 | 2.42096 |
| 18217764 | FBtr0302419 // CG3366 | CG33669 | FBtr0302419 | FBtr0302419 | 4.11E-05 | 1.82528 | 1.82528 |
| 18186494 | FBtr0083164 // CG5399 | CG5399 | FBtr0083164 | FBtr0083164 | 4.14E-05 | 0.665565 | -1.50248 |
| 18199074 | FBtr0084481 // CG1018 | CG10184 | FBtr0084481 | FBtr0084481 | 4.22E-05 | 1.70653 | 1.70653 |
| 18132577 | FBtr0080801 // beat-Ic | beat-Ic | FBtr0080801 | FBtr0080801 | 4.30E-05 | 1.70433 | 1.70433 |
| 18136466 | FBtr0299873 // CG3160 | CG31609 | FBtr0299873 | FBtr0299873 | 4.37E-05 | 1.74626 | 1.74626 |
| 18138528 | FBtr0308744 // CG4340 | CG43401 | FBtr0308744 | FBtr0308744 | 4.40E-05 | 0.269202 | -3.71468 |
| 18164357 | NM_057464 // proPO-A | proPO-A1 | NM_057464 | FBtr0302291 | 4.47E-05 | 1.59056 | 1.59056 |
| 18201178 | FBtr0290052 // CG3109 | CG31097 | FBtr0290052 | FBtr0290052 | 4.48E-05 | 1.89393 | 1.89393 |
| 18206495 | FBtr0077211 // mst // | mst | FBtr0077211 | FBtr0077211 | 4.48E-05 | 0.534206 | -1.87194 |
| 18216199 | FBtr0113000 // CG6361 | CG6361 | FBtr0113000 | FBtr0113000 | 4.55E-05 | 0.506948 | -1.97259 |
| 18171905 | FBtr0091930 // CG3392 | CG33926 | FBtr0091930 | FBtr0091930 | 4.56E-05 | 1.66518 | 1.66518 |
| 18143982 | FBtr0081276 // CG1033 | CG10337 | FBtr0081276 | FBtr0081276 | 4.67E-05 | 1.67231 | 1.67231 |
| 18218891 | NR_047862 // CR43493 | CR43493 | NR_047862 | FBtr0309524 | 4.69E-05 | 0.526352 | -1.89987 |
| 18135262 | FBtr0080500 // CG7110 | CG7110 | FBtr0080500 | FBtr0080500 | 4.70E-05 | 0.551668 | -1.81268 |
| 18153185 | NM_166395 // Obp57a | Obp57a | NM_166395 | FBtr0086294 | 4.77E-05 | 0.591447 | -1.69077 |
| 18164936 | NR_048104 // snoRNA: | snoRNA:2R:9445410 | NR_048104 | FBtr0309758 | 4.88E-05 | 1.99583 | 1.99583 |
| 18153897 | FBtr0072448 // CG3043 | CG30430 | FBtr0072448 | FBtr0072448 | 4.89E-05 | 1.6688 | 1.6688 |
| 18140935 | FBtr0080783 // CG1525 | CG15254 | FBtr0080783 | FBtr0080783 | 4.97E-05 | 2.50736 | 2.50736 |
| 18201684 | FBtr0082844 // CG3146 | CG31469 | FBtr0082844 | FBtr0082844 | 5.02E-05 | 1.64552 | 1.64552 |
| 18169812 | FBtr0075181 // CG7542 | CG7542 | FBtr0075181 | FBtr0075181 | 5.20E-05 | 1.56467 | 1.56467 |
| 18179836 | FBtr0076241 // CG3206 | CG32069 | FBtr0076241 | FBtr0076241 | 5.26E-05 | 1.98983 | 1.98983 |
| 18214885 | FBtr0112968 // CG1791 | CG1791 | FBtr0112968 | FBtr0112968 | 5.31E-05 | 0.480707 | -2.08027 |
| 18178875 | FBtr0333845 // CG9451 | CG9451 | FBtr0333845 | FBtr0333845 | 5.33E-05 | 0.642709 | -1.55591 |
| 18152409 | FBtr0072000 // CG1354 | CG13540 | FBtr0072000 | FBtr0072000 | 5.38E-05 | 0.45958 | -2.1759 |
| 18165245 | FBtr0301790 // CG4137 | CG41378 | FBtr0301790 | FBtr0301790 | 5.39E-05 | 0.628361 | -1.59144 |
| 18173921 | FBtr0332065 // ImpL3 / | ImpL3 | FBtr0332065 | FBtr0332065 | 5.65E-05 | 0.650156 | -1.53809 |
| 18146614 | NR_047994 // CR43358 | CR43358 | NR_047994 | FBtr0307108 | 5.72E-05 | 3.3614 | 3.3614 |
| 18176417 | FBtr0073366 // CG1841 | CG18418 | FBtr0073366 | FBtr0073366 | 5.76E-05 | 1.61126 | 1.61126 |
| 18182534 | FBtr0081954 // osk // o | osk | FBtr0081954 | FBtr0081954 | 5.84E-05 | 2.45653 | 2.45653 |
| 18136421 | NM_134713 // GluRIIC | GluRIIC | NM_134713 | FBtr0336969 | 5.86E-05 | 1.91913 | 1.91913 |
| 18164218 | FBtr0087715 // CG3005 | CG30059 | FBtr0087715 | FBtr0087715 | 5.88E-05 | 0.257318 | -3.88624 |
| 18192132 | FBtr0304645 // CG4306 | CG43061 | FBtr0304645 | FBtr0304645 | 5.89E-05 | 3.9571 | 3.9571 |
| 18153919 | FBtr0086338 // CG3044 | CG30447 | FBtr0086338 | FBtr0086338 | 5.93E-05 | 1.6723 | 1.6723 |
| 18172282 | FBtr0077040 // Jon65Ai | Jon65Aiv | FBtr0077040 | FBtr0077040 | 6.18E-05 | 0.660281 | -1.51451 |
| 18208317 | FBtr0071390 // CG9689 | CG9689 | FBtr0071390 | FBtr0071390 | 6.19E-05 | 1.896 | 1.896 |
| 18186565 | FBtr0083203 // blp // bl | blp | FBtr0083203 | FBtr0083203 | 6.23E-05 | 1.69997 | 1.69997 |
| 18215464 | FBtr0299543 // CG1340 | CG13404 | FBtr0299543 | FBtr0299543 | 6.31E-05 | 0.588803 | -1.69836 |
| 18136730 | FBtr0081025 // CG3174 | CG31742 | FBtr0081025 | FBtr0081025 | 6.39E-05 | 0.553778 | -1.80578 |
| 18199183 | FBtr0304156 // CG1360 | CG13607 | FBtr0304156 | FBtr0304156 | 6.49E-05 | 0.665785 | -1.50199 |
| 18138505 | FBtr0080334 // Phae1 / | Phae1 | FBtr0080334 | FBtr0080334 | 6.54E-05 | 1.58681 | 1.58681 |
| 18173220 | FBtr0304959 // CG4312 | CG43120 | FBtr0304959 | FBtr0304959 | 6.58E-05 | 1.69149 | 1.69149 |
| 18146051 | FBtr0299969 // CG4237 | CG42370 | FBtr0299969 | FBtr0299969 | 6.63E-05 | 1.65242 | 1.65242 |
| 18214864 | FBtr0071403 // CG3106 | CG3106 | FBtr0071403 | FBtr0071403 | 6.63E-05 | 0.584827 | -1.70991 |
| 18147083 | FBtr0088704 // Jon44E | Jon44E | FBtr0088704 | FBtr0088704 | 6.66E-05 | 1.56471 | 1.56471 |
| 18181543 | NR_048223 // mir-2282 | mir-2282 | NR_048223 | FBtr0304359 | 6.69E-05 | 0.616574 | -1.62187 |
| 18143040 | FBtr0321259 // CG5322 | CG5322 | FBtr0321259 | FBtr0321259 | 6.72E-05 | 0.45704 | -2.18799 |
| 18213884 | FBtr0070414 // l(1)G01 | l(1)G0144 | FBtr0070414 | FBtr0070414 | 6.73E-05 | 0.663052 | -1.50818 |
| 18161630 | FBtr0071782 // CG3290 | CG3290 | FBtr0071782 | FBtr0071782 | 6.74E-05 | 1.65096 | 1.65096 |
| 18208961 | FBtr0073908 // CG1461 | CG1461 | FBtr0073908 | FBtr0073908 | 6.75E-05 | 0.577616 | -1.73126 |
| 18132521 | FBtr0080549 // CG7916 | CG7916 | FBtr0080549 | FBtr0080549 | 6.76E-05 | 0.632564 | -1.58087 |
| 18134595 | FBtr0079878 // CG4594 | CG4594 | FBtr0079878 | FBtr0079878 | 6.83E-05 | 0.519526 | -1.92483 |
| 18164749 | FBtr0307501 // Ptr // P | Ptr | FBtr0307501 | FBtr0307501 | 6.84E-05 | 1.50156 | 1.50156 |
| 18171553 | FBtr0076806 // CG3237 | CG32373 | FBtr0076806 | FBtr0076806 | 6.95E-05 | 2.29023 | 2.29023 |
| 18151402 | FBtr0086663 // CG1810 | CG18107 | FBtr0086663 | FBtr0086663 | 6.96E-05 | 2.40295 | 2.40295 |
| 18142015 | FBtr0079280 // CG9500 | CG9500 | FBtr0079280 | FBtr0079280 | 7.14E-05 | 2.73108 | 2.73108 |
| 18169509 | FBtr0075502 // CG6244 | CG6244 | FBtr0075502 | FBtr0075502 | 7.17E-05 | 1.54163 | 1.54163 |
| 18180761 | FBtr0112760 // CG3445 | CG34454 | FBtr0112760 | FBtr0112760 | 7.28E-05 | 0.650073 | -1.53829 |
| 18145433 | FBtr0302372 // CG3404 | CG34049 | FBtr0302372 | FBtr0302372 | 7.31E-05 | 0.49774 | -2.00908 |
| 18169554 | FBtr0075437 // CG5895 | CG5895 | FBtr0075437 | FBtr0075437 | 7.45E-05 | 0.650221 | -1.53794 |
| 18141236 | FBtr0077854 // CG4259 | CG4259 | FBtr0077854 | FBtr0077854 | 7.66E-05 | 2.24326 | 2.24326 |
| 18175510 | FBtr0075722 // Lk // Le | Lk | FBtr0075722 | FBtr0075722 | 7.72E-05 | 1.74543 | 1.74543 |
| 18215817 | FBtr0074240 // CG9914 | CG9914 | FBtr0074240 | FBtr0074240 | 7.73E-05 | 0.624969 | -1.60008 |
| 18158210 | FBtr0112940 // CG3530 | CG3530 | FBtr0112940 | FBtr0112940 | 7.79E-05 | 1.63527 | 1.63527 |
| 18190607 | FBtr0083441 // CG3333 | CG33333 | FBtr0083441 | FBtr0083441 | 7.86E-05 | 2.1265 | 2.1265 |
| 18147139 | NM_206179 // mei-W6 | mei-W68 | NM_206179 | FBtr0086461 | 7.97E-05 | 1.57457 | 1.57457 |
| 18215169 | FBtr0331748 // Sclp // | Sclp | FBtr0331748 | FBtr0331748 | 7.97E-05 | 1.75187 | 1.75187 |
| 18135555 | FBtr0081197 // CG1308 | CG13086 | FBtr0081197 | FBtr0081197 | 8.07E-05 | 1.57399 | 1.57399 |
| 18176606 | FBtr0077064 // CG1046 | CG10469 | FBtr0077064 | FBtr0077064 | 8.08E-05 | 0.616471 | -1.62214 |
| 18186765 | FBtr0083438 // CG5860 | CG5860 | FBtr0083438 | FBtr0083438 | 8.12E-05 | 0.332236 | -3.00991 |
| 18176228 | FBtr0073172 // CG1276 | CG12766 | FBtr0073172 | FBtr0073172 | 8.41E-05 | 0.547516 | -1.82643 |
| 18167064 | FBtr0072653 // CG9129 | CG9129 | FBtr0072653 | FBtr0072653 | 8.65E-05 | 1.60353 | 1.60353 |
| 18166757 | NM_001202157 // Vha | Vha16-3 | NM_001202157 | FBtr0302359 | 8.69E-05 | 1.81854 | 1.81854 |
| 18210889 | FBtr0112557 // CG3434 | CG34348 | FBtr0112557 | FBtr0112557 | 8.86E-05 | 0.616116 | -1.62307 |
| 18179471 | FBtr0078484 // CG7130 | CG7130 | FBtr0078484 | FBtr0078484 | 8.86E-05 | 1.69135 | 1.69135 |
| 18190671 | FBtr0100078 // CG3402 | CG34023 | FBtr0100078 | FBtr0100078 | 8.98E-05 | 1.51106 | 1.51106 |
| 18178058 | FBtr0075665 // CG1231 | CG12310 | FBtr0075665 | FBtr0075665 | 9.04E-05 | 1.61201 | 1.61201 |
| 18134351 | FBtr0079651 // CG1729 | CG17294 | FBtr0079651 | FBtr0079651 | 9.07E-05 | 1.56759 | 1.56759 |
| 18180440 | FBtr0076063 // Muc68E | Muc68E | FBtr0076063 | FBtr0076063 | 9.33E-05 | 1.725 | 1.725 |
| 18133981 | FBtr0079336 // CG1737 | CG17378 | FBtr0079336 | FBtr0079336 | 9.50E-05 | 0.613007 | -1.6313 |
| 18148532 | FBtr0087978 // Drep-1 | Drep-1 | FBtr0087978 | FBtr0087978 | 9.55E-05 | 0.610527 | -1.63793 |
| 18150531 | FBtr0087627 // CG1836 | CG18368 | FBtr0087627 | FBtr0087627 | 9.55E-05 | 0.664404 | -1.50511 |
| 18139440 | FBtr0081347 // vls // v | vls | FBtr0081347 | FBtr0081347 | 9.59E-05 | 1.79324 | 1.79324 |
| 18217032 | FBtr0071481 // CG1264 | CG12643 | FBtr0071481 | FBtr0071481 | 9.88E-05 | 1.65117 | 1.65117 |
| 18138457 | FBtr0306842 // CG4330 | CG43307 | FBtr0306842 | FBtr0306842 | 9.90E-05 | 0.618695 | -1.61631 |
| 18181860 | NR_048216 // scaRNA: | scaRNA:pUf68-a | NR_048216 | FBtr0309770 | 9.93E-05 | 1.50006 | 1.50006 |
| 18147398 | NM_079118 // betaTub | betaTub60D | NM_079118 | FBtr0072270 | 0.00010081 | 0.563102 | -1.77588 |
| 18147029 | FBtr0086754 // fj // fou | fj | FBtr0086754 | FBtr0086754 | 0.00010101 | 0.621752 | -1.60836 |
| 18167633 | FBtr0073149 // CG1200 | CG12006 | FBtr0073149 | FBtr0073149 | 0.00010132 | 0.637247 | -1.56925 |
| 18211133 | FBtr0299571 // CG4225 | CG42259 | FBtr0299571 | FBtr0299571 | 0.00010204 | 0.542484 | -1.84337 |
| 18153786 | FBtr0333002 // CG3035 | CG30354 | FBtr0333002 | FBtr0333002 | 0.0001049 | 0.438783 | -2.27903 |
| 18149513 | FBtr0088690 // Cyp6a1 | Cyp6a13 | FBtr0088690 | FBtr0088690 | 0.00010632 | 1.90476 | 1.90476 |
| 18141312 | FBtr0077781 // CG3528 | CG3528 | FBtr0077781 | FBtr0077781 | 0.0001074 | 0.516219 | -1.93716 |
| 18144692 | FBtr0077893 // CG3166 | CG31663 | FBtr0077893 | FBtr0077893 | 0.00010772 | 0.599902 | -1.66694 |
| 18155617 | FBtr0305124 // CG4312 | CG43123 | FBtr0305124 | FBtr0305124 | 0.00010839 | 0.424793 | -2.35409 |
| 18199478 | FBtr0084873 // CG1366 | CG13663 | FBtr0084873 | FBtr0084873 | 0.00010926 | 1.61419 | 1.61419 |
| 18157348 | NM_057861 // T3dh // | T3dh | NM_057861 | FBtr0071848 | 0.00011132 | 0.611791 | -1.63455 |
| 18188849 | FBtr0085328 // CG1452 | CG14529 | FBtr0085328 | FBtr0085328 | 0.00011184 | 2.00552 | 2.00552 |
| 18218022 | NR_002463 // snoRNA: | snoRNA:Psi28S-3436b | NR_002463 | FBtr0091803 | 0.00011204 | 1.59751 | 1.59751 |
| 18160127 | FBtr0087646 // CG6337 | CG6337 | FBtr0087646 | FBtr0087646 | 0.00011359 | 0.542377 | -1.84373 |
| 18179432 | FBtr0078454 // CG1456 | CG14567 | FBtr0078454 | FBtr0078454 | 0.00011648 | 1.61133 | 1.61133 |
| 18177141 | FBtr0076552 // CG5280 | CG5280 | FBtr0076552 | FBtr0076552 | 0.00011714 | 1.72277 | 1.72277 |
| 18196690 | FBtr0100320 // fabp // | fabp | FBtr0100320 | FBtr0100320 | 0.00011728 | 1.65365 | 1.65365 |
| 18208587 | FBtr0073557 // Karl // | Karl | FBtr0073557 | FBtr0073557 | 0.00011796 | 1.75772 | 1.75772 |
| 18139278 | NM_057296 // Acp26A | Acp26Aa | NM_057296 | FBtr0079155 | 0.00011803 | 0.646611 | -1.54653 |
| 18180334 | FBtr0078461 // CG3244 | CG32447 | FBtr0078461 | FBtr0078461 | 0.00011807 | 1.83647 | 1.83647 |
| 18167125 | FBtr0072693 // FucTD / | FucTD | FBtr0072693 | FBtr0072693 | 0.00011965 | 0.614984 | -1.62606 |
| 18150999 | FBtr0087137 // CG1570 | CG15705 | FBtr0087137 | FBtr0087137 | 0.0001211 | 0.649656 | -1.53928 |
| 18138595 | NR_047877 // mir-4914 | mir-4914 | NR_047877 | FBtr0309709 | 0.00012128 | 0.648755 | -1.54141 |
| 18151879 | FBtr0071514 // CG1522 | CG15227 | FBtr0071514 | FBtr0071514 | 0.00012441 | 0.55792 | -1.79237 |
| 18166760 | NM_168459 // Vha16-2 | Vha16-2 | NM_168459 | FBtr0076091 | 0.00012469 | 2.82877 | 2.82877 |
| 18217832 | NR_003743 // snoRNA: | snoRNA:Psi28S-1135e | NR_003743 | FBtr0113570 | 0.00012659 | 1.57842 | 1.57842 |
| 18218586 | FBtr0074377 // SmG // | SmG | FBtr0074377 | FBtr0074377 | 0.00013116 | 1.51666 | 1.51666 |
| 18153228 | NM_136665 // GstT1 // | GstT1 | NM_136665 | FBtr0088463 | 0.00013179 | 1.70374 | 1.70374 |
| 18211858 | FBtr0306816 // CG4328 | CG43288 | FBtr0306816 | FBtr0306816 | 0.00013246 | 0.394747 | -2.53327 |
| 18152170 | FBtr0071827 // Mes4 // | Mes4 | FBtr0071827 | FBtr0071827 | 0.00013463 | 1.53541 | 1.53541 |
| 18212309 | FBtr0073841 // l(1)dd4 | l(1)dd4 | FBtr0073841 | FBtr0073841 | 0.00013645 | 0.566319 | -1.76579 |
| 18152089 | FBtr0071720 // CG9294 | CG9294 | FBtr0071720 | FBtr0071720 | 0.00013842 | 0.468478 | -2.13457 |
| 18217755 | FBtr0302415 // CG3366 | CG33666 | FBtr0302415 | FBtr0302415 | 0.00013843 | 0.645147 | -1.55003 |
| 18154613 | FBtr0112725 // CG3442 | CG34424 | FBtr0112725 | FBtr0112725 | 0.00014821 | 0.5907 | -1.69291 |
| 18147957 | NM_057704 // Odc1 // | Odc1 | NM_057704 | FBtr0088863 | 0.00014848 | 0.654031 | -1.52898 |
| 18154123 | FBtr0087284 // CG3346 | CG33462 | FBtr0087284 | FBtr0087284 | 0.00015111 | 0.466859 | -2.14197 |
| 18142139 | FBtr0290242 // CG1132 | CG11322 | FBtr0290242 | FBtr0290242 | 0.00015124 | 1.69162 | 1.69162 |
| 18213037 | FBtr0074375 // CG1586 | CG15865 | FBtr0074375 | FBtr0074375 | 0.00015304 | 0.551236 | -1.8141 |
| 18148601 | NM_058059 // CkIIbeta | CkIIbeta2 | NM_058059 | FBtr0086278 | 0.00015499 | 1.53992 | 1.53992 |
| 18154945 | FBtr0299961 // CG4236 | CG42362 | FBtr0299961 | FBtr0299961 | 0.00015651 | 0.421854 | -2.37049 |
| 18154949 | FBtr0299961 // CG4236 | CG42362 | FBtr0299961 | FBtr0299961 | 0.00015651 | 0.421854 | -2.37049 |
| 18168741 | NM_079288 // Ilp2 // I | Ilp2 | NM_079288 | FBtr0076329 | 0.00015734 | 1.62086 | 1.62086 |
| 18173316 | FBtr0305909 // CG4317 | CG43174 | FBtr0305909 | FBtr0305909 | 0.00015904 | 1.86891 | 1.86891 |
| 18203048 | FBtr0302437 // CG4265 | CG42656 | FBtr0302437 | FBtr0302437 | 0.00015985 | 1.59304 | 1.59304 |
| 18197296 | NM_001260160 // Pk1r | Pk1r | NM_001260160 | FBtr0305681 | 0.00016027 | 0.622856 | -1.60551 |
| 18146841 | NR_073765 // CR43818 | CR43818 | NR_073765 | FBtr0332211 | 0.00016074 | 1.63269 | 1.63269 |
| 18182266 | FBtr0084660 // ash2 // | ash2 | FBtr0084660 | FBtr0084660 | 0.00016082 | 0.642559 | -1.55628 |
| 18160107 | FBtr0087686 // CG6145 | CG6145 | FBtr0087686 | FBtr0087686 | 0.0001621 | 0.632637 | -1.58069 |
| 18181193 | FBtr0301744 // CG4259 | CG42590 | FBtr0301744 | FBtr0301744 | 0.00016224 | 0.442396 | -2.26042 |
| 18161362 | FBtr0071561 // CG1799 | CG17999 | FBtr0071561 | FBtr0071561 | 0.00016296 | 0.639521 | -1.56367 |
| 18183067 | FBtr0082572 // GstD5 / | GstD5 | FBtr0082572 | FBtr0082572 | 0.0001633 | 2.50407 | 2.50407 |
| 18144137 | FBtr0290042 // Oseg5 / | Oseg5 | FBtr0290042 | FBtr0290042 | 0.00016903 | 2.03527 | 2.03527 |
| 18203101 | FBtr0082548 // Cpn // | Cpn | FBtr0082548 | FBtr0082548 | 0.00016964 | 0.605142 | -1.65251 |
| 18205120 | FBtr0302216 // CG4024 | CG40249 | FBtr0302216 | FBtr0302216 | 0.00017142 | 1.82791 | 1.82791 |
| 18200221 | FBtr0085514 // capa // | capa | FBtr0085514 | FBtr0085514 | 0.00017145 | 1.87685 | 1.87685 |
| 18172297 | FBtr0112435 // CG3424 | CG34241 | FBtr0112435 | FBtr0112435 | 0.00017292 | 1.57025 | 1.57025 |
| 18169617 | FBtr0075465 // CG1306 | CG13067 | FBtr0075465 | FBtr0075465 | 0.00017299 | 1.54709 | 1.54709 |
| 18182342 | NM_142415 // DNaseII | DNaseII | NM_142415 | FBtr0083538 | 0.00017442 | 0.665151 | -1.50342 |
| 18214931 | FBtr0333306 // Psf3 // | Psf3 | FBtr0333306 | FBtr0333306 | 0.00017558 | 0.550662 | -1.816 |
| 18150416 | FBtr0113076 // wuc // | wuc | FBtr0113076 | FBtr0113076 | 0.00017573 | 1.57229 | 1.57229 |
| 18176058 | FBtr0073011 // spz5 // | spz5 | FBtr0073011 | FBtr0073011 | 0.00017635 | 0.518253 | -1.92956 |
| 18138004 | FBtr0302734 // wb // w | wb | FBtr0302734 | FBtr0302734 | 0.00017859 | 0.660602 | -1.51377 |
| 18154247 | NR_001759 // snoRNA: | snoRNA:U29:54Ec | NR_001759 | FBtr0086847 | 0.00018344 | 0.54722 | -1.82742 |
| 18200422 | FBtr0085723 // Npc2g / | Npc2g | FBtr0085723 | FBtr0085723 | 0.00018458 | 1.67529 | 1.67529 |
| 18154241 | NR_001765 // snoRNA: | snoRNA:U31:54Eb | NR_001765 | FBtr0086853 | 0.00018605 | 0.535618 | -1.867 |
| 18153634 | FBtr0273311 // CG3026 | CG30268 | FBtr0273311 | FBtr0273311 | 0.00018679 | 0.551528 | -1.81314 |
| 18209201 | FBtr0301666 // CG8909 | CG8909 | FBtr0301666 | FBtr0301666 | 0.00018693 | 1.52964 | 1.52964 |
| 18131802 | NM_057866 // Rca1 // | Rca1 | NM_057866 | FBtr0079342 | 0.00018979 | 0.569581 | -1.75568 |
| 18165929 | FBtr0076613 // mtrm // | mtrm | FBtr0076613 | FBtr0076613 | 0.00019213 | 0.485497 | -2.05974 |
| 18154249 | NR_001758 // snoRNA: | snoRNA:U29:54Eb | NR_001758 | FBtr0086846 | 0.00019431 | 0.597893 | -1.67254 |
| 18168152 | FBtr0076913 // CG1582 | CG15829 | FBtr0076913 | FBtr0076913 | 0.00019476 | 2.2829 | 2.2829 |
| 18163675 | FBtr0112391 // CG3419 | CG34198 | FBtr0112391 | FBtr0112391 | 0.00019849 | 1.62585 | 1.62585 |
| 18176125 | FBtr0073111 // CG1496 | CG14963 | FBtr0073111 | FBtr0073111 | 0.00020001 | 0.61176 | -1.63463 |
| 18184755 | NM_079521 // NPFR1 / | NPFR1 | NM_079521 | FBtr0078590 | 0.0002004 | 0.516158 | -1.93739 |
| 18170858 | NM_206410 // asparag | asparagine-synthetase | NM_206410 | FBtr0089463 | 0.000202 | 1.5581 | 1.5581 |
| 18131338 | FBtr0080281 // Cry // C | Cry | FBtr0080281 | FBtr0080281 | 0.00020575 | 1.69138 | 1.69138 |
| 18149890 | FBtr0310142 // CG1291 | CG12910 | FBtr0310142 | FBtr0310142 | 0.00020861 | 0.554944 | -1.80198 |
| 18212788 | FBtr0071424 // Yp2 // Y | Yp2 | FBtr0071424 | FBtr0071424 | 0.00021088 | 0.520402 | -1.92159 |
| 18212581 | NR_002129 // snRNA:U | snRNA:U5:14B | NR_002129 | FBtr0074249 | 0.00021202 | 2.11284 | 2.11284 |
| 18136891 | FBtr0077757 // CG3194 | CG31949 | FBtr0077757 | FBtr0077757 | 0.00021362 | 1.65082 | 1.65082 |
| 18214998 | FBtr0073396 // CG1552 | CG1552 | FBtr0073396 | FBtr0073396 | 0.00021675 | 0.662993 | -1.50831 |
| 18162802 | FBtr0071927 // CG3019 | CG30196 | FBtr0071927 | FBtr0071927 | 0.00021783 | 2.34936 | 2.34936 |
| 18169436 | FBtr0075615 // CG3325 | CG33259 | FBtr0075615 | FBtr0075615 | 0.00021866 | 1.92945 | 1.92945 |
| 18138736 | NR_073750 // CR43766 | CR43766 | NR_073750 | FBtr0331283 | 0.00022161 | 1.87563 | 1.87563 |
| 18168757 | FBtr0301227 // CG8065 | CG8065 | FBtr0301227 | FBtr0301227 | 0.00022389 | 1.5037 | 1.5037 |
| 18133002 | FBtr0077956 // CG4552 | CG4552 | FBtr0077956 | FBtr0077956 | 0.00022731 | 0.662992 | -1.50831 |
| 18163066 | FBtr0072041 // CG3041 | CG30417 | FBtr0072041 | FBtr0072041 | 0.00022927 | 0.565257 | -1.76911 |
| 18217618 | FBtr0071239 // CG3322 | CG33223 | FBtr0071239 | FBtr0071239 | 0.00023083 | 0.447684 | -2.23372 |
| 18216541 | FBtr0070027 // Phf7 // | Phf7 | FBtr0070027 | FBtr0070027 | 0.00023241 | 1.55387 | 1.55387 |
| 18217486 | FBtr0070881 // CG3275 | CG32750 | FBtr0070881 | FBtr0070881 | 0.00023332 | 1.9204 | 1.9204 |
| 18164414 | FBtr0302904 // CG4269 | CG42697 | FBtr0302904 | FBtr0302904 | 0.00023436 | 0.663195 | -1.50785 |
| 18156074 | NM_080421 // Amy-p / | Amy-p | NM_080421 | FBtr0087004 | 0.0002344 | 1.50609 | 1.50609 |
| 18134874 | FBtr0080107 // CG1710 | CG17107 | FBtr0080107 | FBtr0080107 | 0.00024151 | 0.606833 | -1.6479 |
| 18211915 | NR_047779 // CR43615 | CR43615 | NR_047779 | FBtr0309826 | 0.00024475 | 1.68775 | 1.68775 |
| 18161727 | FBtr0071928 // Cyp6d2 | Cyp6d2 | FBtr0071928 | FBtr0071928 | 0.00024786 | 2.16212 | 2.16212 |
| 18212088 | FBtr0070504 // CG2650 | CG2650 | FBtr0070504 | FBtr0070504 | 0.00025162 | 1.86515 | 1.86515 |
| 18209853 | FBtr0074745 // Sec61g | Sec61gamma | FBtr0074745 | FBtr0074745 | 0.00025719 | 0.419071 | -2.38623 |
| 18200255 | FBtr0085603 // CG7920 | CG7920 | FBtr0085603 | FBtr0085603 | 0.00025878 | 0.660252 | -1.51457 |
| 18133759 | NM_135074 // Cyp4ac3 | Cyp4ac3 | NM_135074 | FBtr0079068 | 0.0002603 | 0.609297 | -1.64124 |
| 18185555 | FBtr0082225 // CG3999 | CG3999 | FBtr0082225 | FBtr0082225 | 0.00026485 | 1.90241 | 1.90241 |
| 18176566 | FBtr0077113 // mad2 // | mad2 | FBtr0077113 | FBtr0077113 | 0.00026825 | 1.74732 | 1.74732 |
| 18185993 | NM_169484 // Cyp304 | Cyp304a1 | NM_169484 | FBtr0082691 | 0.00027102 | 2.21937 | 2.21937 |
| 18146923 | NM_079044 // Amy-d / | Amy-d | NM_079044 | FBtr0086983 | 0.00027191 | 0.618378 | -1.61713 |
| 18193664 | FBtr0083502 // Edg91 / | Edg91 | FBtr0083502 | FBtr0083502 | 0.00027638 | 2.77947 | 2.77947 |
| 18144354 | FBtr0079713 // Peritrop | Peritrophin-15a | FBtr0079713 | FBtr0079713 | 0.00028314 | 0.400559 | -2.49651 |
| 18152265 | NM_176249 // Obp59a | Obp59a | NM_176249 | FBtr0071870 | 0.00028509 | 0.610817 | -1.63715 |
| 18132956 | FBtr0078005 // CG1394 | CG13947 | FBtr0078005 | FBtr0078005 | 0.00028773 | 2.28181 | 2.28181 |
| 18164938 | NR_048146 // snoRNA: | snoRNA:hts-a | NR_048146 | FBtr0309763 | 0.00028849 | 1.6351 | 1.6351 |
| 18165122 | NR_073898 // CR43793 | CR43793 | NR_073898 | FBtr0331988 | 0.00028871 | 0.662864 | -1.5086 |
| 18190932 | FBtr0112495 // CG3429 | CG34299 | FBtr0112495 | FBtr0112495 | 0.00029229 | 0.551064 | -1.81467 |
| 18213687 | FBtr0307095 // CG1763 | CG17636 | FBtr0307095 | FBtr0307095 | 0.00029587 | 0.498998 | -2.00401 |
| 18202255 | FBtr0112479 // CG3428 | CG34283 | FBtr0112479 | FBtr0112479 | 0.00029827 | 0.565631 | -1.76794 |
| 18210192 | FBtr0070163 // CG1166 | CG11664 | FBtr0070163 | FBtr0070163 | 0.0003001 | 1.66031 | 1.66031 |
| 18187506 | FBtr0321268 // CG7084 | CG7084 | FBtr0321268 | FBtr0321268 | 0.00030643 | 0.663102 | -1.50806 |
| 18152252 | FBtr0309801 // CG1351 | CG13511 | FBtr0309801 | FBtr0309801 | 0.00031046 | 1.58702 | 1.58702 |
| 18149642 | NM_001201960 // GstE | GstE13 | NM_001201960 | FBtr0302495 | 0.00031082 | 1.54587 | 1.54587 |
| 18161905 | FBtr0072107 // CG1766 | CG17664 | FBtr0072107 | FBtr0072107 | 0.00031512 | 0.517962 | -1.93064 |
| 18216319 | FBtr0074703 // CG1588 | CG15882 | FBtr0074703 | FBtr0074703 | 0.00031633 | 1.60629 | 1.60629 |
| 18166270 | NM_079232 // mei-P22 | mei-P22 | NM_079232 | FBtr0076836 | 0.00033356 | 1.80036 | 1.80036 |
| 18195018 | FBtr0085094 // BM-40- | BM-40-SPARC | FBtr0085094 | FBtr0085094 | 0.00033624 | 1.61061 | 1.61061 |
| 18166810 | NM_001144525 // CS-2 | CS-2 | NM_001144525 | FBtr0308081 | 0.00033839 | 0.568717 | -1.75835 |
| 18153348 | FBtr0087905 // CG3004 | CG30047 | FBtr0087905 | FBtr0087905 | 0.00033897 | 0.657988 | -1.51978 |
| 18143886 | FBtr0081233 // CG1756 | CG17568 | FBtr0081233 | FBtr0081233 | 0.0003393 | 1.71463 | 1.71463 |
| 18196561 | FBtr0082298 // CG1469 | CG14694 | FBtr0082298 | FBtr0082298 | 0.00034183 | 0.643558 | -1.55386 |
| 18160624 | FBtr0087121 // CG8311 | CG8311 | FBtr0087121 | FBtr0087121 | 0.00034545 | 0.615261 | -1.62533 |
| 18177020 | NM_139964 // Oseg1 / | Oseg1 | NM_139964 | FBtr0076663 | 0.00035682 | 1.89878 | 1.89878 |
| 18154235 | NR_001757 // snoRNA: | snoRNA:Me28S-A1666a | NR_001757 | FBtr0086845 | 0.00036285 | 0.428058 | -2.33613 |
| 18208684 | FBtr0073655 // CG2543 | CG2543 | FBtr0073655 | FBtr0073655 | 0.00036489 | 0.524091 | -1.90807 |
| 18154231 | NR_001763 // snoRNA: | snoRNA:Me28S-G3277a | NR_001763 | FBtr0086851 | 0.0003654 | 0.54284 | -1.84216 |
| 18163056 | FBtr0302316 // CG3041 | CG30414 | FBtr0302316 | FBtr0302316 | 0.00036633 | 1.60605 | 1.60605 |
| 18196392 | FBtr0306611 // CG5359 | CG5359 | FBtr0306611 | FBtr0306611 | 0.00037006 | 0.635374 | -1.57387 |
| 18149558 | FBtr0088708 // PGRP-S | PGRP-SC1b | FBtr0088708 | FBtr0088708 | 0.00037217 | 1.92716 | 1.92716 |
| 18208796 | FBtr0073768 // dmrt11 | dmrt11E | FBtr0073768 | FBtr0073768 | 0.00037543 | 0.389635 | -2.5665 |
| 18179592 | FBtr0075674 // CG1346 | CG13465 | FBtr0075674 | FBtr0075674 | 0.00038109 | 2.39682 | 2.39682 |
| 18143083 | FBtr0080125 // CG7300 | CG7300 | FBtr0080125 | FBtr0080125 | 0.00038188 | 1.68391 | 1.68391 |
| 18151208 | FBtr0086919 // CG1076 | CG10764 | FBtr0086919 | FBtr0086919 | 0.00038747 | 0.532165 | -1.87912 |
| 18157088 | FBtr0087455 // Cyp6a8 | Cyp6a8 | FBtr0087455 | FBtr0087455 | 0.00039774 | 0.634031 | -1.57721 |
| 18183073 | NM_080177 // GstD8 / | GstD8 | NM_080177 | FBtr0082575 | 0.00040093 | 1.96192 | 1.96192 |
| 18154360 | NR_003823 // snoRNA: | snoRNA:Psi18S-1389b | NR_003823 | FBtr0113526 | 0.00041151 | 1.81322 | 1.81322 |
| 18198913 | FBtr0303508 // CG1711 | CG17111 | FBtr0303508 | FBtr0303508 | 0.0004116 | 0.657437 | -1.52106 |
| 18178112 | FBtr0332815 // CG7804 | CG7804 | FBtr0332815 | FBtr0332815 | 0.00041836 | 1.64027 | 1.64027 |
| 18190455 | FBtr0081582 // CG3149 | CG31496 | FBtr0081582 | FBtr0081582 | 0.00042054 | 0.614012 | -1.62863 |
| 18207395 | FBtr0070526 // CG1080 | CG10801 | FBtr0070526 | FBtr0070526 | 0.00042096 | 1.69604 | 1.69604 |
| 18151443 | FBtr0113093 // CG1092 | CG10924 | FBtr0113093 | FBtr0113093 | 0.00042949 | 2.2136 | 2.2136 |
| 18153708 | FBtr0113368 // Ipk1 // | Ipk1 | FBtr0113368 | FBtr0113368 | 0.00043223 | 0.646755 | -1.54618 |
| 18203237 | NR_048426 // mir-997 | mir-997 | NR_048426 | FBtr0304363 | 0.0004331 | 0.616268 | -1.62267 |
| 18193265 | FBtr0082662 // mus308 | mus308 | FBtr0082662 | FBtr0082662 | 0.00043835 | 2.03935 | 2.03935 |
| 18217144 | NR_002468 // pncr004: | pncr004:X | NR_002468 | FBtr0091949 | 0.00044077 | 0.568365 | -1.75943 |
| 18195064 | NM_142091 // Aats-me | Aats-met | NM_142091 | FBtr0082895 | 0.00046924 | 0.664734 | -1.50436 |
| 18137797 | FBtr0300301 // CG4246 | CG42465 | FBtr0300301 | FBtr0300301 | 0.00047391 | 1.9498 | 1.9498 |
| 18168155 | FBtr0076833 // CG1482 | CG14826 | FBtr0076833 | FBtr0076833 | 0.00048307 | 0.521864 | -1.91621 |
| 18160568 | FBtr0087182 // CG7755 | CG7755 | FBtr0087182 | FBtr0087182 | 0.00048419 | 0.625474 | -1.59879 |
| 18177695 | FBtr0075989 // GRHRII | GRHRII | FBtr0075989 | FBtr0075989 | 0.00049552 | 0.608268 | -1.64401 |
| 18164863 | FBtr0112385 // robls54 | robls54B | FBtr0112385 | FBtr0112385 | 0.00050096 | 1.8262 | 1.8262 |
| 18172483 | FBtr0301683 // sinah // | sinah | FBtr0301683 | FBtr0301683 | 0.00050466 | 0.632354 | -1.58139 |
| 18170035 | FBtr0332754 // Cpr76B | Cpr76Bc | FBtr0332754 | FBtr0332754 | 0.00051351 | 1.50657 | 1.50657 |
| 18146689 | NR_048018 // CR43606 | CR43606 | NR_048018 | FBtr0309806 | 0.00053804 | 1.64191 | 1.64191 |
| 18182219 | FBtr0301800 // CG4259 | CG42598 | FBtr0301800 | FBtr0301800 | 0.00054322 | 3.54211 | 3.54211 |
| 18146854 | NR_073807 // CR43839 | CR43839 | NR_073807 | FBtr0332319 | 0.00054864 | 2.07631 | 2.07631 |
| 18145531 | NM_206014 // CheB38 | CheB38a | NM_206014 | FBtr0081453 | 0.00055593 | 0.563523 | -1.77455 |
| 18163907 | FBtr0333065 // jeb // je | jeb | FBtr0333065 | FBtr0333065 | 0.00055684 | 1.72453 | 1.72453 |
| 18155919 | NM_001273825 // CG4 | CG43646 | NM_001273825 | FBtr0310091 | 0.00055952 | 1.51941 | 1.51941 |
| 18151681 | FBtr0086463 // CG1512 | CG15120 | FBtr0086463 | FBtr0086463 | 0.0005701 | 2.1527 | 2.1527 |
| 18177560 | FBtr0301326 // CG6053 | CG6053 | FBtr0301326 | FBtr0301326 | 0.0005749 | 1.71668 | 1.71668 |
| 18137466 | FBtr0112356 // CG3416 | CG34165 | FBtr0112356 | FBtr0112356 | 0.00057684 | 1.67256 | 1.67256 |
| 18197383 | FBtr0082981 // CG8066 | CG8066 | FBtr0082981 | FBtr0082981 | 0.00057897 | 0.606524 | -1.64874 |
| 18168494 | FBtr0076512 // CG1331 | CG13313 | FBtr0076512 | FBtr0076512 | 0.00058011 | 1.86346 | 1.86346 |
| 18143308 | FBtr0080360 // rho-6 // | rho-6 | FBtr0080360 | FBtr0080360 | 0.00058461 | 1.77127 | 1.77127 |
| 18164956 | NR_048046 // mir-4975 | mir-4975 | NR_048046 | FBtr0309586 | 0.00059163 | 1.64723 | 1.64723 |
| 18139558 | FBtr0112854 // Ets21C | Ets21C | FBtr0112854 | FBtr0112854 | 0.00059875 | 1.61785 | 1.61785 |
| 18209719 | FBtr0074669 // Mec2 // | Mec2 | FBtr0074669 | FBtr0074669 | 0.00060769 | 1.5883 | 1.5883 |
| 18216640 | FBtr0077266 // CG1544 | CG15449 | FBtr0077266 | FBtr0077266 | 0.0006292 | 0.654338 | -1.52826 |
| 18160439 | FBtr0087291 // CG8299 | CG8299 | FBtr0087291 | FBtr0087291 | 0.00064103 | 0.47817 | -2.09131 |
| 18136286 | NM_078819 // Gr32a // | Gr32a | NM_078819 | FBtr0080198 | 0.00064738 | 0.579818 | -1.72468 |
| 18181547 | FBtr0304642 // CG4305 | CG43059 | FBtr0304642 | FBtr0304642 | 0.00065363 | 0.652756 | -1.53197 |
| 18177840 | FBtr0075936 // CG1094 | CG10943 | FBtr0075936 | FBtr0075936 | 0.0006699 | 0.521076 | -1.9191 |
| 18151643 | FBtr0310321 // CG1007 | CG10073 | FBtr0310321 | FBtr0310321 | 0.00067022 | 0.42761 | -2.33858 |
| 18144739 | FBtr0081365 // CG3168 | CG31683 | FBtr0081365 | FBtr0081365 | 0.00068267 | 0.511688 | -1.95431 |
| 18162671 | FBtr0086963 // CG3010 | CG30101 | FBtr0086963 | FBtr0086963 | 0.00068293 | 1.78792 | 1.78792 |
| 18170809 | FBtr0273402 // CG8620 | CG8620 | FBtr0273402 | FBtr0273402 | 0.00070084 | 0.567882 | -1.76093 |
| 18174631 | NM_206253 // Pxn // P | Pxn | NM_206253 | FBtr0072954 | 0.00070904 | 1.50289 | 1.50289 |
| 18192543 | NR_048355 // snoRNA: | snoRNA:dmt-a | NR_048355 | FBtr0309776 | 0.00071881 | 1.54078 | 1.54078 |
| 18201204 | FBtr0273357 // CG3110 | CG31105 | FBtr0273357 | FBtr0273357 | 0.00072107 | 0.566879 | -1.76405 |
| 18207651 | FBtr0070808 // CG1577 | CG15772 | FBtr0070808 | FBtr0070808 | 0.0007266 | 1.51901 | 1.51901 |
| 18152278 | FBtr0071880 // CG1352 | CG13527 | FBtr0071880 | FBtr0071880 | 0.00073948 | 0.635845 | -1.57271 |
| 18150112 | FBtr0304901 // CG1320 | CG13203 | FBtr0304901 | FBtr0304901 | 0.00075559 | 0.617754 | -1.61877 |
| 18148118 | FBtr0088586 // unpg // | unpg | FBtr0088586 | FBtr0088586 | 0.00075987 | 1.65861 | 1.65861 |
| 18137500 | FBtr0300048 // CG3418 | CG34180 | FBtr0300048 | FBtr0300048 | 0.00077559 | 0.515263 | -1.94076 |
| 18172649 | FBtr0301252 // CG4255 | CG42553 | FBtr0301252 | FBtr0301252 | 0.00079183 | 1.62143 | 1.62143 |
| 18133474 | FBtr0077516 // CG2772 | CG2772 | FBtr0077516 | FBtr0077516 | 0.00079718 | 0.613037 | -1.63122 |
| 18180580 | FBtr0111152 // CG4047 | CG40470 | FBtr0111152 | FBtr0111152 | 0.00083282 | 0.664603 | -1.50466 |
| 18187824 | FBtr0303150 // CG1671 | CG16710 | FBtr0303150 | FBtr0303150 | 0.00087304 | 1.86625 | 1.86625 |
| 18135060 | NM_080363 // Gr33a // | Gr33a | NM_080363 | FBtr0080337 | 0.00088093 | 2.53333 | 2.53333 |
| 18132226 | NM_080269 // Ugt37b1 | Ugt37b1 | NM_080269 | FBtr0079242 | 0.0008963 | 1.97998 | 1.97998 |
| 18186310 | FBtr0082957 // CG1485 | CG14852 | FBtr0082957 | FBtr0082957 | 0.00090244 | 2.0524 | 2.0524 |
| 18173102 | NR_048219 // mir-282 | mir-282 | NR_048219 | FBtr0304174 | 0.00090341 | 1.56873 | 1.56873 |
| 18169068 | FBtr0075954 // CG1041 | CG10418 | FBtr0075954 | FBtr0075954 | 0.00090643 | 1.54966 | 1.54966 |
| 18208539 | FBtr0073509 // PGRP-S | PGRP-SA | FBtr0073509 | FBtr0073509 | 0.00091119 | 1.73008 | 1.73008 |
| 18131728 | NM_078727 // ast // as | ast | NM_078727 | FBtr0077952 | 0.00092474 | 0.63838 | -1.56646 |
| 18175765 | FBtr0072734 // CG9192 | CG9192 | FBtr0072734 | FBtr0072734 | 0.00093954 | 1.80084 | 1.80084 |
| 18193278 | FBtr0084472 // nau // n | nau | FBtr0084472 | FBtr0084472 | 0.00096108 | 0.651987 | -1.53377 |
| 18203403 | FBtr0306166 // CG4322 | CG43222 | FBtr0306166 | FBtr0306166 | 0.00096245 | 1.51735 | 1.51735 |
| 18159518 | FBtr0088248 // Lsm10 / | Lsm10 | FBtr0088248 | FBtr0088248 | 0.00096547 | 1.52793 | 1.52793 |
| 18151439 | NM_137495 // GstE11 | GstE11 | NM_137495 | FBtr0086697 | 0.00098864 | 1.50987 | 1.50987 |
| 18181917 | NM_001274925 // CG4 | CG43678 | NM_001274925 | FBtr0310458 | 0.00099067 | 1.73542 | 1.73542 |
| 18145065 | NM_164466 // Tengl2 / | Tengl2 | NM_164466 | FBtr0077808 | 0.00100814 | 1.70811 | 1.70811 |
| 18200333 | FBtr0085638 // CG9733 | CG9733 | FBtr0085638 | FBtr0085638 | 0.0010296 | 1.8922 | 1.8922 |
| 18180372 | FBtr0073007 // CG3248 | CG32487 | FBtr0073007 | FBtr0073007 | 0.001031 | 1.73642 | 1.73642 |
| 18136830 | FBtr0080350 // CG3186 | CG31862 | FBtr0080350 | FBtr0080350 | 0.00106449 | 1.53179 | 1.53179 |
| 18217518 | FBtr0070703 // CG3277 | CG32773 | FBtr0070703 | FBtr0070703 | 0.00107142 | 1.66393 | 1.66393 |
| 18192162 | NR_048362 // mir-34 // | mir-34 | NR_048362 | FBtr0304499 | 0.00107175 | 2.19711 | 2.19711 |
| 18131155 | NR_001600 // snRNA:U | snRNA:U3:22A | NR_001600 | FBtr0077928 | 0.00107559 | 2.44765 | 2.44765 |
| 18216175 | FBtr0300182 // upd2 // | upd2 | FBtr0300182 | FBtr0300182 | 0.00108446 | 2.05441 | 2.05441 |
| 18163385 | FBtr0091496 // Acp53C | Acp53C14c | FBtr0091496 | FBtr0091496 | 0.00109062 | 0.554385 | -1.8038 |
| 18203234 | NM_001202325 // Mtn | MtnE | NM_001202325 | FBtr0304129 | 0.00112326 | 0.653349 | -1.53057 |
| 18168995 | FBtr0076042 // CG1782 | CG17826 | FBtr0076042 | FBtr0076042 | 0.00112361 | 1.67953 | 1.67953 |
| 18144705 | FBtr0303377 // CG3166 | CG31664 | FBtr0303377 | FBtr0303377 | 0.00114181 | 1.57277 | 1.57277 |
| 18195856 | FBtr0081773 // CG3014 | CG3014 | FBtr0081773 | FBtr0081773 | 0.00114549 | 0.618273 | -1.61741 |
| 18198503 | FBtr0083935 // CG4000 | CG4000 | FBtr0083935 | FBtr0083935 | 0.00120174 | 0.578242 | -1.72938 |
| 18191841 | NR_048474 // scaRNA: | scaRNA:mgU2-25 | NR_048474 | FBtr0309753 | 0.00122769 | 1.56528 | 1.56528 |
| 18165426 | FBtr0303474 // Hsp67B | Hsp67Bb | FBtr0303474 | FBtr0303474 | 0.00124631 | 2.08111 | 2.08111 |
| 18167624 | FBtr0300062 // CG1035 | CG10357 | FBtr0300062 | FBtr0300062 | 0.00127171 | 1.66615 | 1.66615 |
| 18196230 | FBtr0082112 // CG1679 | CG16790 | FBtr0082112 | FBtr0082112 | 0.0012866 | 0.630054 | -1.58717 |
| 18207332 | FBtr0070078 // CG3176 | CG3176 | FBtr0070078 | FBtr0070078 | 0.0012872 | 1.72811 | 1.72811 |
| 18157185 | NM_079124 // Ssl // Su | Ssl | NM_079124 | FBtr0072351 | 0.0013039 | 1.50402 | 1.50402 |
| 18163737 | FBtr0301011 // CG3423 | CG34236 | FBtr0301011 | FBtr0301011 | 0.00130718 | 0.622481 | -1.60647 |
| 18137380 | NR_003764 // snoRNA: | snoRNA:Psi18S-525a | NR_003764 | FBtr0113539 | 0.00132785 | 1.85022 | 1.85022 |
| 18197229 | FBtr0082861 // CG9759 | CG9759 | FBtr0082861 | FBtr0082861 | 0.00133065 | 0.594583 | -1.68185 |
| 18191425 | FBtr0299852 // CG4233 | CG42335 | FBtr0299852 | FBtr0299852 | 0.00140592 | 1.63462 | 1.63462 |
| 18161610 | FBtr0071787 // CG3045 | CG3045 | FBtr0071787 | FBtr0071787 | 0.00143132 | 1.57038 | 1.57038 |
| 18150672 | FBtr0087488 // CG1738 | CG17386 | FBtr0087488 | FBtr0087488 | 0.00148398 | 0.557028 | -1.79524 |
| 18158274 | FBtr0086955 // mthl3 / | mthl3 | FBtr0086955 | FBtr0086955 | 0.0015229 | 0.54338 | -1.84033 |
| 18133919 | FBtr0079253 // CG9505 | CG9505 | FBtr0079253 | FBtr0079253 | 0.00154921 | 0.647165 | -1.5452 |
| 18141140 | FBtr0077985 // Tfb4 // | Tfb4 | FBtr0077985 | FBtr0077985 | 0.00155611 | 0.625079 | -1.5998 |
| 18168840 | NM_079298 // mRpL2 / | mRpL2 | NM_079298 | FBtr0076211 | 0.00158823 | 1.50462 | 1.50462 |
| 18174590 | FBtr0076299 // can // c | can | FBtr0076299 | FBtr0076299 | 0.00160199 | 0.603485 | -1.65704 |
| 18169114 | FBtr0075868 // CG1412 | CG14120 | FBtr0075868 | FBtr0075868 | 0.00161014 | 1.60708 | 1.60708 |
| 18177529 | FBtr0076140 // Muc68 | Muc68Ca | FBtr0076140 | FBtr0076140 | 0.0016142 | 1.56367 | 1.56367 |
| 18136612 | FBtr0302224 // CG3168 | CG31687 | FBtr0302224 | FBtr0302224 | 0.00163253 | 0.501852 | -1.99262 |
| 18167307 | FBtr0072871 // CG8960 | CG8960 | FBtr0072871 | FBtr0072871 | 0.00163259 | 0.391159 | -2.55651 |
| 18192084 | FBtr0303835 // CG4282 | CG42828 | FBtr0303835 | FBtr0303835 | 0.00163634 | 0.579037 | -1.72701 |
| 18215873 | FBtr0074297 // CG9903 | CG9903 | FBtr0074297 | FBtr0074297 | 0.00169453 | 0.564859 | -1.77035 |
| 18154441 | FBtr0112401 // CG3420 | CG34208 | FBtr0112401 | FBtr0112401 | 0.00170622 | 0.580424 | -1.72288 |
| 18192598 | NR_074011 // CR43642 | CR43642 | NR_074011 | FBtr0310043 | 0.00173551 | 1.88369 | 1.88369 |
| 18153079 | FBtr0088506 // wun2 // | wun2 | FBtr0088506 | FBtr0088506 | 0.00173598 | 0.554361 | -1.80388 |
| 18213491 | FBtr0070501 // Csat // | Csat | FBtr0070501 | FBtr0070501 | 0.00174992 | 0.524759 | -1.90564 |
| 18172199 | FBtr0112768 // CG3446 | CG34462 | FBtr0112768 | FBtr0112768 | 0.00175934 | 0.519608 | -1.92453 |
| 18133338 | FBtr0077722 // CG2964 | CG2964 | FBtr0077722 | FBtr0077722 | 0.00176031 | 0.580122 | -1.72378 |
| 18184513 | FBtr0303866 // Mur82C | Mur82C | FBtr0303866 | FBtr0303866 | 0.00177225 | 1.68429 | 1.68429 |
| 18161367 | FBtr0071560 // CG9993 | CG9993 | FBtr0071560 | FBtr0071560 | 0.00177817 | 0.608664 | -1.64294 |
| 18141223 | FBtr0077892 // CG1535 | CG15358 | FBtr0077892 | FBtr0077892 | 0.00187889 | 0.618484 | -1.61686 |
| 18218134 | FBtr0299576 // CG4226 | CG42262 | FBtr0299576 | FBtr0299576 | 0.00189036 | 1.51928 | 1.51928 |
| 18144309 | FBtr0308305 // lectin-2 | lectin-21Cb | FBtr0308305 | FBtr0308305 | 0.00191064 | 1.76892 | 1.76892 |
| 18140456 | FBtr0330647 // CG1159 | CG11592 | FBtr0330647 | FBtr0330647 | 0.00195682 | 0.577187 | -1.73254 |
| 18183291 | FBtr0082638 // Hsp70B | Hsp70Bc | FBtr0082638 | FBtr0082638 | 0.00197257 | 3.4369 | 3.4369 |
| 18162944 | FBtr0088772 // hubl // | hubl | FBtr0088772 | FBtr0088772 | 0.00197462 | 0.608817 | -1.64253 |
| 18203730 | NR_074050 // CR43846 | CR43846 | NR_074050 | FBtr0332548 | 0.00198252 | 1.75572 | 1.75572 |
| 18166800 | FBtr0072592 // nerfin-1 | nerfin-1 | FBtr0072592 | FBtr0072592 | 0.00199921 | 1.71701 | 1.71701 |
| 18203810 | FBtr0302251 // CG4015 | CG40155 | FBtr0302251 | FBtr0302251 | 0.00202953 | 1.58492 | 1.58492 |
| 18211378 | FBtr0073767 // Syt12 // | Syt12 | FBtr0073767 | FBtr0073767 | 0.00207119 | 0.622673 | -1.60598 |
| 18154233 | NR_001760 // snoRNA: | snoRNA:U76:54Eb | NR_001760 | FBtr0086848 | 0.00208275 | 0.527142 | -1.89702 |
| 18144916 | FBtr0080809 // CG3182 | CG31821 | FBtr0080809 | FBtr0080809 | 0.00212586 | 0.62749 | -1.59365 |
| 18146111 | FBtr0302203 // CG4246 | CG42463 | FBtr0302203 | FBtr0302203 | 0.00213225 | 0.658796 | -1.51792 |
| 18134439 | FBtr0079767 // CG1243 | CG12439 | FBtr0079767 | FBtr0079767 | 0.00213261 | 2.4448 | 2.4448 |
| 18210315 | FBtr0307297 // Ste12D | Ste12DOR | FBtr0307297 | FBtr0307297 | 0.00213365 | 0.552664 | -1.80942 |
| 18199408 | FBtr0084729 // CG1364 | CG13641 | FBtr0084729 | FBtr0084729 | 0.00214293 | 1.59963 | 1.59963 |
| 18217637 | FBtr0300094 // Ste:CG3 | Ste:CG33245 | FBtr0300094 | FBtr0300094 | 0.00215434 | 0.60322 | -1.65777 |
| 18170030 | FBtr0074930 // CG1251 | CG12519 | FBtr0074930 | FBtr0074930 | 0.0021559 | 0.483694 | -2.06742 |
| 18176455 | FBtr0077180 // CG1067 | CG10673 | FBtr0077180 | FBtr0077180 | 0.00215653 | 0.634261 | -1.57664 |
| 18214777 | FBtr0071264 // CG1266 | CG12662 | FBtr0071264 | FBtr0071264 | 0.0022092 | 0.65461 | -1.52763 |
| 18201018 | NM_170497 // PH4alph | PH4alphaSG1 | NM_170497 | FBtr0085686 | 0.00221638 | 0.614938 | -1.62618 |
| 18162328 | FBtr0089002 // kappaB | kappaB-Ras | FBtr0089002 | FBtr0089002 | 0.00224007 | 0.433399 | -2.30735 |
| 18203193 | NR_048407 // CR42839 | CR42839 | NR_048407 | FBtr0309032 | 0.00227179 | 0.642521 | -1.55637 |
| 18217661 | FBtr0300095 // Ste:CG3 | Ste:CG33246 | FBtr0300095 | FBtr0300095 | 0.00227731 | 0.603572 | -1.6568 |
| 18191942 | NR_037766 // CR42745 | CR42745 | NR_037766 | FBtr0303214 | 0.00229425 | 1.59071 | 1.59071 |
| 18154229 | NR_001764 // snoRNA: | snoRNA:snR38:54Eb | NR_001764 | FBtr0086852 | 0.00231088 | 0.388857 | -2.57164 |
| 18140556 | NM_078883 // Ugt37a1 | Ugt37a1 | NM_078883 | FBtr0081372 | 0.00233833 | 1.94709 | 1.94709 |
| 18217621 | FBtr0300085 // Ste:CG3 | Ste:CG33245 | FBtr0300085 | FBtr0300085 | 0.00234443 | 0.595546 | -1.67913 |
| 18196834 | FBtr0082504 // CG3397 | CG3397 | FBtr0082504 | FBtr0082504 | 0.00238237 | 0.60796 | -1.64484 |
| 18215350 | FBtr0332298 // CG1181 | CG11816 | FBtr0332298 | FBtr0332298 | 0.00238363 | 1.80321 | 1.80321 |
| 18181035 | FBtr0332760 // CG4244 | CG42445 | FBtr0332760 | FBtr0332760 | 0.00239097 | 1.58668 | 1.58668 |
| 18188473 | FBtr0084987 // CG6142 | CG6142 | FBtr0084987 | FBtr0084987 | 0.00240643 | 1.50815 | 1.50815 |
| 18148567 | NM_166277 // IM2 // I | IM2 | NM_166277 | FBtr0086664 | 0.00243594 | 1.62045 | 1.62045 |
| 18177768 | FBtr0332644 // CG1065 | CG10654 | FBtr0332644 | FBtr0332644 | 0.00250608 | 1.51155 | 1.51155 |
| 18180661 | NR_003858 // snoRNA: | snoRNA:Psi18S-1397 | NR_003858 | FBtr0113606 | 0.00250729 | 1.5178 | 1.5178 |
| 18176177 | FBtr0300753 // CG1207 | CG12077 | FBtr0300753 | FBtr0300753 | 0.00252374 | 1.65486 | 1.65486 |
| 18215026 | FBtr0073473 // CG1519 | CG15199 | FBtr0073473 | FBtr0073473 | 0.00253272 | 1.66571 | 1.66571 |
| 18154142 | NM_001014533 // Vkor | Vkor | NM_001014533 | FBtr0091510 | 0.00253936 | 2.07037 | 2.07037 |
| 18161564 | FBtr0071733 // CG1349 | CG13494 | FBtr0071733 | FBtr0071733 | 0.00254441 | 1.6512 | 1.6512 |
| 18215546 | FBtr0073977 // CG1440 | CG14406 | FBtr0073977 | FBtr0073977 | 0.00257091 | 0.618802 | -1.61603 |
| 18184956 | FBtr0303219 // CG1460 | CG14608 | FBtr0303219 | FBtr0303219 | 0.00260907 | 1.76536 | 1.76536 |
| 18155410 | FBtr0303193 // CG4273 | CG42736 | FBtr0303193 | FBtr0303193 | 0.00261292 | 1.52758 | 1.52758 |
| 18207391 | FBtr0332493 // CG3603 | CG3603 | FBtr0332493 | FBtr0332493 | 0.00262969 | 0.622755 | -1.60577 |
| 18193068 | FBtr0084589 // Hsp68 / | Hsp68 | FBtr0084589 | FBtr0084589 | 0.00262993 | 1.55301 | 1.55301 |
| 18145527 | NM_206013 // CheB38 | CheB38b | NM_206013 | FBtr0081454 | 0.00263827 | 1.79815 | 1.79815 |
| 18201648 | FBtr0113407 // CG3142 | CG31427 | FBtr0113407 | FBtr0113407 | 0.00268079 | 1.88552 | 1.88552 |
| 18164723 | FBtr0306050 // CG4318 | CG43189 | FBtr0306050 | FBtr0306050 | 0.00273219 | 1.61077 | 1.61077 |
| 18155904 | NR_048199 // mir-4985 | mir-4985 | NR_048199 | FBtr0309660 | 0.0027356 | 0.591624 | -1.69026 |
| 18208910 | FBtr0073818 // CG1113 | CG11134 | FBtr0073818 | FBtr0073818 | 0.00278179 | 1.59282 | 1.59282 |
| 18216050 | FBtr0074444 // CG8661 | CG8661 | FBtr0074444 | FBtr0074444 | 0.00279376 | 0.639953 | -1.56261 |
| 18152414 | FBtr0072002 // CG9861 | CG9861 | FBtr0072002 | FBtr0072002 | 0.0028513 | 0.503852 | -1.98471 |
| 18185335 | FBtr0082016 // HP1e // | HP1e | FBtr0082016 | FBtr0082016 | 0.00286448 | 0.604155 | -1.6552 |
| 18136960 | FBtr0079737 // CG3298 | CG32983 | FBtr0079737 | FBtr0079737 | 0.00287362 | 1.77205 | 1.77205 |
| 18154128 | FBtr0087656 // CG3347 | CG33470 | FBtr0087656 | FBtr0087656 | 0.00288248 | 0.545562 | -1.83297 |
| 18144526 | FBtr0081310 // TotF // | TotF | FBtr0081310 | FBtr0081310 | 0.00290243 | 1.73308 | 1.73308 |
| 18178565 | NM_140749 // Adgf-B / | Adgf-B | NM_140749 | FBtr0075193 | 0.002908 | 0.60992 | -1.63956 |
| 18183061 | NM_080173 // GstD2 / | GstD2 | NM_080173 | FBtr0082569 | 0.00297386 | 1.65363 | 1.65363 |
| 18180564 | FBtr0290083 // CG3402 | CG34025 | FBtr0290083 | FBtr0290083 | 0.0030987 | 2.1015 | 2.1015 |
| 18203842 | FBtr0302249 // CG4262 | CG42621 | FBtr0302249 | FBtr0302249 | 0.00316138 | 1.72498 | 1.72498 |
| 18188478 | FBtr0084990 // CG1229 | CG12290 | FBtr0084990 | FBtr0084990 | 0.0032024 | 0.600573 | -1.66508 |
| 18138319 | NR_047995 // let-7-C / | let-7-C | NR_047995 | FBtr0306973 | 0.00322821 | 0.620306 | -1.61211 |
| 18159022 | FBtr0088724 // CG8584 | CG8584 | FBtr0088724 | FBtr0088724 | 0.00324413 | 0.661675 | -1.51131 |
| 18146541 | FBtr0305706 // CG4316 | CG43165 | FBtr0305706 | FBtr0305706 | 0.00335447 | 1.5481 | 1.5481 |
| 18215476 | FBtr0073916 // CG9411 | CG9411 | FBtr0073916 | FBtr0073916 | 0.00343939 | 0.604029 | -1.65555 |
| 18198869 | FBtr0084314 // CG4704 | CG4704 | FBtr0084314 | FBtr0084314 | 0.00344895 | 1.56547 | 1.56547 |
| 18132093 | NM_078839 // Acyp // | Acyp | NM_078839 | FBtr0080555 | 0.00347388 | 1.58455 | 1.58455 |
| 18150307 | FBtr0087924 // Cpr49A | Cpr49Ah | FBtr0087924 | FBtr0087924 | 0.00348321 | 2.08358 | 2.08358 |
| 18218616 | NR_047818 // mir-927 | mir-927 | NR_047818 | FBtr0304254 | 0.00351659 | 1.63937 | 1.63937 |
| 18185521 | NM_141699 // eloF // e | eloF | NM_141699 | FBtr0082144 | 0.00364603 | 0.50171 | -1.99318 |
| 18132184 | FBtr0077874 // Or22b / | Or22b | FBtr0077874 | FBtr0077874 | 0.00367914 | 1.80144 | 1.80144 |
| 18138309 | NR_047967 // mir-9c // | mir-9c | NR_047967 | FBtr0304398 | 0.0037638 | 1.5651 | 1.5651 |
| 18154096 | FBtr0086169 // ppk25 / | ppk25 | FBtr0086169 | FBtr0086169 | 0.00376707 | 1.59545 | 1.59545 |
| 18162413 | NM_137914 // Gr59d // | Gr59d | NM_137914 | FBtr0071976 | 0.00380296 | 1.67155 | 1.67155 |
| 18154253 | NR_001770 // snoRNA: | snoRNA:Me18S-A28a | NR_001770 | FBtr0086858 | 0.00387689 | 0.551185 | -1.81427 |
| 18154364 | NR_003807 // snoRNA: | snoRNA:Psi18S-1347b | NR_003807 | FBtr0113514 | 0.00388524 | 0.511477 | -1.95512 |
| 18219266 | FBtr0113889 // Ppr-Y // | Ppr-Y | FBtr0113889 | FBtr0113889 | 0.00406704 | 1.57264 | 1.57264 |
| 18152895 | FBtr0072393 // CG3770 | CG3770 | FBtr0072393 | FBtr0072393 | 0.00407826 | 2.03027 | 2.03027 |
| 18173351 | NR_048295 // CR43280 | CR43280 | NR_048295 | FBtr0306721 | 0.00420301 | 1.65778 | 1.65778 |
| 18174196 | FBtr0075112 // term // | term | FBtr0075112 | FBtr0075112 | 0.00420979 | 1.7493 | 1.7493 |
| 18195703 | FBtr0078623 // CG1145 | CG11459 | FBtr0078623 | FBtr0078623 | 0.00431516 | 1.50593 | 1.50593 |
| 18200430 | FBtr0085719 // CG1554 | CG15545 | FBtr0085719 | FBtr0085719 | 0.00441286 | 0.636615 | -1.57081 |
| 18199724 | FBtr0085078 // CG6296 | CG6296 | FBtr0085078 | FBtr0085078 | 0.00446399 | 0.649743 | -1.53907 |
| 18146698 | NR_047928 // CR43621 | CR43621 | NR_047928 | FBtr0309976 | 0.00450831 | 1.9741 | 1.9741 |
| 18200651 | FBtr0308027 // Ugt86D | Ugt86Dg | FBtr0308027 | FBtr0308027 | 0.004756 | 1.84501 | 1.84501 |
| 18203806 | FBtr0302250 // CG1583 | CG15831 | FBtr0302250 | FBtr0302250 | 0.00497314 | 1.77922 | 1.77922 |
| 18171908 | FBtr0100006 // CG3396 | CG33965 | FBtr0100006 | FBtr0100006 | 0.0050375 | 0.54435 | -1.83705 |
| 18217625 | FBtr0300094 // Ste:CG3 | Ste:CG33245 | FBtr0300094 | FBtr0300094 | 0.00504529 | 0.611192 | -1.63615 |
| 18217633 | FBtr0300094 // Ste:CG3 | Ste:CG33245 | FBtr0300094 | FBtr0300094 | 0.00504529 | 0.611192 | -1.63615 |
| 18217653 | FBtr0300094 // Ste:CG3 | Ste:CG33245 | FBtr0300094 | FBtr0300094 | 0.00504529 | 0.611192 | -1.63615 |
| 18217657 | FBtr0300094 // Ste:CG3 | Ste:CG33245 | FBtr0300094 | FBtr0300094 | 0.00504529 | 0.611192 | -1.63615 |
| 18160841 | FBtr0089560 // CG6385 | CG6385 | FBtr0089560 | FBtr0089560 | 0.00508756 | 0.63609 | -1.57211 |
| 18138532 | FBtr0308746 // CG4340 | CG43403 | FBtr0308746 | FBtr0308746 | 0.00522734 | 0.64264 | -1.55608 |
| 18161298 | NM_166397 // Obp57d | Obp57d | NM_166397 | FBtr0299804 | 0.00523813 | 0.610268 | -1.63862 |
| 18162481 | NM_166397 // Obp57d | Obp57d | NM_166397 | FBtr0299804 | 0.00523813 | 0.610268 | -1.63862 |
| 18203717 | NM_001275946 // CG4 | CG43694 | NM_001275946 | FBtr0310577 | 0.00543952 | 1.51583 | 1.51583 |
| 18182316 | FBtr0085616 // CecC // | CecC | FBtr0085616 | FBtr0085616 | 0.00566582 | 1.63027 | 1.63027 |
| 18199481 | FBtr0302299 // Cad96C | Cad96Cb | FBtr0302299 | FBtr0302299 | 0.00573667 | 1.58008 | 1.58008 |
| 18206397 | FBtr0071201 // Cp7Fb / | Cp7Fb | FBtr0071201 | FBtr0071201 | 0.00578355 | 0.57322 | -1.74453 |
| 18164771 | FBtr0306826 // CG4329 | CG43296 | FBtr0306826 | FBtr0306826 | 0.00581686 | 1.57129 | 1.57129 |
| 18141647 | NM_135019 // Ir25a // | Ir25a | NM_135019 | FBtr0289979 | 0.00591842 | 1.57402 | 1.57402 |
| 18138822 | NR_073730 // CR43866 | CR43866 | NR_073730 | FBtr0332601 | 0.00628336 | 1.50923 | 1.50923 |
| 18146323 | FBtr0302989 // CG4271 | CG42711 | FBtr0302989 | FBtr0302989 | 0.00628466 | 1.72684 | 1.72684 |
| 18137350 | NR_003775 // snoRNA: | snoRNA:Psi18S-640e | NR_003775 | FBtr0113550 | 0.00629757 | 2.15495 | 2.15495 |
| 18217649 | FBtr0300092 // Ste:CG3 | Ste:CG33243 | FBtr0300092 | FBtr0300092 | 0.00640974 | 0.614174 | -1.6282 |
| 18182313 | FBtr0085614 // CecA2 / | CecA2 | FBtr0085614 | FBtr0085614 | 0.00651409 | 0.537692 | -1.8598 |
| 18180062 | FBtr0303006 // CG3224 | CG32241 | FBtr0303006 | FBtr0303006 | 0.00654861 | 1.5092 | 1.5092 |
| 18146552 | NR_047983 // CR43239 | CR43239 | NR_047983 | FBtr0306294 | 0.00669191 | 0.658291 | -1.51908 |
| 18217641 | FBtr0300090 // Ste:CG3 | Ste:CG33239 | FBtr0300090 | FBtr0300090 | 0.00682368 | 0.519404 | -1.92529 |
| 18204225 | NR_003113 // sphinx // | sphinx | NR_003113 | FBtr0111045 | 0.00686438 | 0.649487 | -1.53968 |
| 18154237 | NR_001767 // snoRNA: | snoRNA:U31:54Ed | NR_001767 | FBtr0086855 | 0.00687172 | 0.599605 | -1.66776 |
| 18215054 | FBtr0073539 // CG1561 | CG1561 | FBtr0073539 | FBtr0073539 | 0.00688157 | 0.660523 | -1.51395 |
| 18144754 | FBtr0085946 // CG3170 | CG31703 | FBtr0085946 | FBtr0085946 | 0.0069073 | 1.77011 | 1.77011 |
| 18155894 | NR_048143 // snoRNA: | snoRNA:abba-a | NR_048143 | FBtr0309772 | 0.00694082 | 1.5456 | 1.5456 |
| 18176596 | FBtr0077067 // Jon65Ai | Jon65Ai | FBtr0077067 | FBtr0077067 | 0.00696561 | 1.71494 | 1.71494 |
| 18177129 | FBtr0076560 // CG5660 | CG5660 | FBtr0076560 | FBtr0076560 | 0.00702002 | 0.633551 | -1.5784 |
| 18179973 | NR_048293 // CR32194 | CR32194 | NR_048293 | FBtr0306733 | 0.00727895 | 0.620796 | -1.61084 |
| 18175099 | FBtr0075207 // Jon74E | Jon74E | FBtr0075207 | FBtr0075207 | 0.00731123 | 1.55735 | 1.55735 |
| 18137364 | NR_003778 // snoRNA: | snoRNA:Psi18S-525i | NR_003778 | FBtr0113553 | 0.00748738 | 1.74199 | 1.74199 |
| 18181104 | FBtr0300949 // CG4253 | CG42536 | FBtr0300949 | FBtr0300949 | 0.00750083 | 1.66466 | 1.66466 |
| 18154165 | NM_001032240 // CG3 | CG33792 | NM_001032240 | FBtr0332251 | 0.00752005 | 1.63427 | 1.63427 |
| 18147090 | FBtr0072449 // Kr // Kr | Kr | FBtr0072449 | FBtr0072449 | 0.00768562 | 0.582843 | -1.71573 |
| 18189337 | FBtr0085790 // mey // | mey | FBtr0085790 | FBtr0085790 | 0.00794053 | 1.65103 | 1.65103 |
| 18162676 | FBtr0086899 // CG3010 | CG30105 | FBtr0086899 | FBtr0086899 | 0.0079885 | 1.63483 | 1.63483 |
| 18133376 | FBtr0331628 // CG1726 | CG17264 | FBtr0331628 | FBtr0331628 | 0.00841523 | 1.51665 | 1.51665 |
| 18200285 | FBtr0302562 // CG1149 | CG11498 | FBtr0302562 | FBtr0302562 | 0.00858978 | 1.58438 | 1.58438 |
| 18132155 | FBtr0112925 // Traf4 // | Traf4 | FBtr0112925 | FBtr0112925 | 0.00870123 | 0.625641 | -1.59836 |

**Supplementary Table S2:** Transcripts altered by selective breeding in longevity-selected *La* flies compared to background *Ra* flies. 448 genes were identified by microarray analysis. Transcripts upregulated in *La* flies are indicated by (+) fold-change and downregulated transcripts with (-).
