# Supplementary material for: Endurance exercise and selective breeding for longevity extend Drosophila healthspan by overlapping mechanisms
Source: Aging (Albany NY). 2015 Aug 8;7(8):535–50. doi: 10.18632/aging.100789 (PMC4586100; doi:10.18632/aging.100789)
Supplement: Supplementary file 4 [file aging-07-535-s004.docx]

**Supplementary Table S3**

| Column ID | Gene  assignment | Gene Symbol | RefSeq | FlyBase  ID | p-value | Ratio | Fold-Change |
| --- | --- | --- | --- | --- | --- | --- | --- |
| 18161552 | FBtr0071740 // CG4 | CG4363 | FBtr0071740 | FBtr0071740 | 3.99E-13 | 2.37373 | 2.37373 |
| 18134388 | FBtr0079684 // CG1 | CG13091 | FBtr0079684 | FBtr0079684 | 5.72E-13 | 0.356347 | -2.80626 |
| 18178314 | FBtr0075424 // Cpr7 | Cpr72Ec | FBtr0075424 | FBtr0075424 | 1.44E-12 | 36.2044 | 36.2044 |
| 18158990 | NM_136539 // Mal- | Mal-A7 | NM_136539 | FBtr0088757 | 2.05E-12 | 0.0134551 | -74.3214 |
| 18173516 | NR_048214 // snoR | snoRNA:CG32479-b | NR_048214 | FBtr0309775 | 1.02E-11 | 21.0124 | 21.0124 |
| 18149502 | NM_136537 // Mal- | Mal-A4 | NM_136537 | FBtr0088748 | 1.07E-11 | 0.0312841 | -31.9651 |
| 18147926 | FBtr0087437 // AttA | AttA | FBtr0087437 | FBtr0087437 | 1.12E-11 | 0.0184553 | -54.185 |
| 18157159 | FBtr0089055 // Cyp9 | Cyp9b2 | FBtr0089055 | FBtr0089055 | 1.44E-11 | 1.70167 | 1.70167 |
| 18132600 | FBtr0080729 // CG1 | CG15263 | FBtr0080729 | FBtr0080729 | 3.06E-11 | 8.52367 | 8.52367 |
| 18147462 | NM_057460 // Dpt / | Dpt | NM_057460 | FBtr0086620 | 7.49E-11 | 0.441445 | -2.26529 |
| 18199522 | FBtr0084852 // CG1 | CG10560 | FBtr0084852 | FBtr0084852 | 7.82E-11 | 0.291278 | -3.43315 |
| 18149978 | FBtr0088213 // CG1 | CG12934 | FBtr0088213 | FBtr0088213 | 8.84E-11 | 14.6455 | 14.6455 |
| 18161522 | FBtr0071682 // pirk / | pirk | FBtr0071682 | FBtr0071682 | 1.58E-10 | 2.5772 | 2.5772 |
| 18151339 | FBtr0089630 // CG1 | CG10910 | FBtr0089630 | FBtr0089630 | 1.63E-10 | 7.76057 | 7.76057 |
| 18176684 | FBtr0076963 // eIF4 | eIF4E-4 | FBtr0076963 | FBtr0076963 | 1.71E-10 | 3.25256 | 3.25256 |
| 18180537 | FBtr0307394 // CG3 | CG33969 | FBtr0307394 | FBtr0307394 | 1.81E-10 | 0.306004 | -3.26793 |
| 18135984 | NM_078820 // hgo / | hgo | NM_078820 | FBtr0080201 | 2.60E-10 | 0.271494 | -3.68332 |
| 18170188 | FBtr0074843 // Spn7 | Spn77Bb | FBtr0074843 | FBtr0074843 | 2.63E-10 | 1.81326 | 1.81326 |
| 18146471 | FBtr0304142 // CG4 | CG42876 | FBtr0304142 | FBtr0304142 | 2.75E-10 | 7.09114 | 7.09114 |
| 18188283 | FBtr0084811 // CG1 | CG11854 | FBtr0084811 | FBtr0084811 | 3.07E-10 | 0.134115 | -7.45628 |
| 18191517 | FBtr0300319 // Sfp8 | Sfp87B | FBtr0300319 | FBtr0300319 | 3.35E-10 | 1.7147 | 1.7147 |
| 18218028 | NR_002464 // snoR | snoRNA:Psi28S-3436a | NR_002464 | FBtr0091752 | 4.02E-10 | 2.52134 | 2.52134 |
| 18216226 | FBtr0074626 // CG1 | CG15043 | FBtr0074626 | FBtr0074626 | 4.44E-10 | 2.02424 | 2.02424 |
| 18151405 | NM_137479 // GstE | GstE1 | NM_137479 | FBtr0086669 | 5.20E-10 | 3.58837 | 3.58837 |
| 18195001 | NM_079589 // Ugt3 | Ugt35b | NM_079589 | FBtr0082375 | 5.62E-10 | 0.341728 | -2.92631 |
| 18133827 | FBtr0306841 // CG1 | CG11034 | FBtr0306841 | FBtr0306841 | 6.79E-10 | 3.83132 | 3.83132 |
| 18156154 | FBtr0086133 // Cyp6 | Cyp6a2 | FBtr0086133 | FBtr0086133 | 7.14E-10 | 9.00459 | 9.00459 |
| 18156951 | FBtr0072455 // PebII | PebII | FBtr0072455 | FBtr0072455 | 7.32E-10 | 1.84161 | 1.84161 |
| 18202078 | FBtr0300407 // CG3 | CG34034 | FBtr0300407 | FBtr0300407 | 9.53E-10 | 1.98456 | 1.98456 |
| 18182175 | FBtr0305959 // CG1 | CG17374 | FBtr0305959 | FBtr0305959 | 1.01E-09 | 0.476341 | -2.09934 |
| 18188365 | FBtr0084879 // CG5 | CG5107 | FBtr0084879 | FBtr0084879 | 1.05E-09 | 2.35654 | 2.35654 |
| 18154395 | FBtr0110768 // Acp5 | Acp54A1 | FBtr0110768 | FBtr0110768 | 1.08E-09 | 4.66098 | 4.66098 |
| 18182240 | NM_079643 // Act88 | Act88F | NM_079643 | FBtr0083143 | 1.14E-09 | 3.52612 | 3.52612 |
| 18163186 | FBtr0089419 // Cyp1 | Cyp12d1-p | FBtr0089419 | FBtr0089419 | 1.20E-09 | 0.541125 | -1.848 |
| 18153106 | FBtr0305795 // AttC | AttC | FBtr0305795 | FBtr0305795 | 1.29E-09 | 0.265076 | -3.77251 |
| 18210607 | FBtr0307489 // CG3 | CG32698 | FBtr0307489 | FBtr0307489 | 1.29E-09 | 1.92502 | 1.92502 |
| 18156279 | FBtr0333557 // Lcp1 | Lcp1 | FBtr0333557 | FBtr0333557 | 1.40E-09 | 25.5499 | 25.5499 |
| 18192073 | FBtr0303828 // CG4 | CG42821 | FBtr0303828 | FBtr0303828 | 1.46E-09 | 2.75212 | 2.75212 |
| 18214629 | FBtr0333310 // CG2 | CG2233 | FBtr0333310 | FBtr0333310 | 1.63E-09 | 1.6242 | 1.6242 |
| 18153188 | FBtr0088709 // PGR | PGRP-SC2 | FBtr0088709 | FBtr0088709 | 1.83E-09 | 1.92399 | 1.92399 |
| 18141537 | FBtr0077550 // CG1 | CG16704 | FBtr0077550 | FBtr0077550 | 2.19E-09 | 11.0223 | 11.0223 |
| 18138585 | NR_073764 // CR43 | CR43051 | NR_073764 | FBtr0334410 | 2.20E-09 | 0.213837 | -4.67646 |
| 18190346 | FBtr0273369 // Npc2 | Npc2e | FBtr0273369 | FBtr0273369 | 2.33E-09 | 0.197547 | -5.06208 |
| 18171442 | FBtr0073059 // Drsl2 | Drsl2 | FBtr0073059 | FBtr0073059 | 2.38E-09 | 0.0687556 | -14.5443 |
| 18146555 | NR_047871 // CR43 | CR43263 | NR_047871 | FBtr0306546 | 2.39E-09 | 0.0443006 | -22.5731 |
| 18172304 | FBtr0076914 // CG8 | CG8628 | FBtr0076914 | FBtr0076914 | 2.47E-09 | 0.158443 | -6.31143 |
| 18160937 | NM_137474 // IM23 | IM23 | NM_137474 | FBtr0086729 | 2.53E-09 | 2.74623 | 2.74623 |
| 18202278 | FBtr0112487 // CG3 | CG34291 | FBtr0112487 | FBtr0112487 | 2.55E-09 | 3.07826 | 3.07826 |
| 18215100 | FBtr0073613 // CG9 | CG9360 | FBtr0073613 | FBtr0073613 | 2.97E-09 | 0.174526 | -5.72981 |
| 18138279 | NR_073764 // CR43 | CR43051 | NR_073764 | FBtr0334410 | 3.89E-09 | 0.174078 | -5.74456 |
| 18216520 | FBtr0308650 // CG1 | CG15618 | FBtr0308650 | FBtr0308650 | 4.01E-09 | 0.114883 | -8.70453 |
| 18160806 | FBtr0086894 // CG1 | CG10936 | FBtr0086894 | FBtr0086894 | 4.39E-09 | 4.20742 | 4.20742 |
| 18155565 | NR_048064 // mir-9 | mir-986 | NR_048064 | FBtr0304479 | 5.17E-09 | 0.491149 | -2.03604 |
| 18142324 | FBtr0079515 // CG7 | CG7025 | FBtr0079515 | FBtr0079515 | 5.39E-09 | 0.145314 | -6.88163 |
| 18188602 | FBtr0085105 // CG1 | CG17189 | FBtr0085105 | FBtr0085105 | 5.41E-09 | 0.374069 | -2.6733 |
| 18131045 | NM_165237 // nina | ninaD | NM_165237 | FBtr0081031 | 5.53E-09 | 0.248438 | -4.02515 |
| 18165360 | NM_001274820 // E | Est-6 | NM_001274820 | FBtr0333383 | 5.65E-09 | 1.87053 | 1.87053 |
| 18142869 | FBtr0079891 // CG5 | CG5846 | FBtr0079891 | FBtr0079891 | 5.87E-09 | 0.169918 | -5.88518 |
| 18216848 | FBtr0070185 // CG3 | CG3690 | FBtr0070185 | FBtr0070185 | 5.87E-09 | 2.1931 | 2.1931 |
| 18153861 | FBtr0072016 // CG3 | CG30411 | FBtr0072016 | FBtr0072016 | 5.96E-09 | 0.300727 | -3.32528 |
| 18140731 | FBtr0080618 // nimB | nimB2 | FBtr0080618 | FBtr0080618 | 6.17E-09 | 3.14071 | 3.14071 |
| 18210765 | FBtr0100081 // CG3 | CG34026 | FBtr0100081 | FBtr0100081 | 6.26E-09 | 2.3423 | 2.3423 |
| 18156749 | FBtr0088160 // epsil | epsilonTry | FBtr0088160 | FBtr0088160 | 6.31E-09 | 3.1724 | 3.1724 |
| 18161213 | FBtr0086325 // CG1 | CG16898 | FBtr0086325 | FBtr0086325 | 8.46E-09 | 4.36622 | 4.36622 |
| 18152233 | FBtr0071854 // CG4 | CG4269 | FBtr0071854 | FBtr0071854 | 8.56E-09 | 2.09016 | 2.09016 |
| 18147667 | NM_079016 // Mdr5 | Mdr50 | NM_079016 | FBtr0087537 | 8.86E-09 | 2.59307 | 2.59307 |
| 18141036 | FBtr0078098 // CG1 | CG11911 | FBtr0078098 | FBtr0078098 | 9.23E-09 | 0.220796 | -4.52907 |
| 18200850 | FBtr0083985 // TotX | TotX | FBtr0083985 | FBtr0083985 | 9.39E-09 | 0.597686 | -1.67312 |
| 18145006 | FBtr0077540 // CG3 | CG31955 | FBtr0077540 | FBtr0077540 | 1.05E-08 | 3.09001 | 3.09001 |
| 18166626 | FBtr0073157 // Fie / | Fie | FBtr0073157 | FBtr0073157 | 1.07E-08 | 1.85742 | 1.85742 |
| 18192300 | FBtr0305917 // CG4 | CG43175 | FBtr0305917 | FBtr0305917 | 1.14E-08 | 5.07403 | 5.07403 |
| 18186681 | FBtr0083330 // CG1 | CG17560 | FBtr0083330 | FBtr0083330 | 1.16E-08 | 0.452831 | -2.20833 |
| 18193421 | FBtr0085511 // Jon9 | Jon99Ci | FBtr0085511 | FBtr0085511 | 1.24E-08 | 2.11717 | 2.11717 |
| 18198686 | NM_142736 // fit // | fit | NM_142736 | FBtr0084153 | 1.27E-08 | 3.37899 | 3.37899 |
| 18174309 | FBtr0072637 // LysX | LysX | FBtr0072637 | FBtr0072637 | 1.31E-08 | 0.173643 | -5.75894 |
| 18178712 | FBtr0075091 // Cyp1 | Cyp12c1 | FBtr0075091 | FBtr0075091 | 1.35E-08 | 0.170211 | -5.87507 |
| 18136636 | FBtr0080273 // CG3 | CG31704 | FBtr0080273 | FBtr0080273 | 1.36E-08 | 2.50275 | 2.50275 |
| 18190622 | FBtr0302527 // CG3 | CG33346 | FBtr0302527 | FBtr0302527 | 1.38E-08 | 2.29969 | 2.29969 |
| 18162478 | NM_166079 // Obp5 | Obp51a | NM_166079 | FBtr0087420 | 1.41E-08 | 1.6756 | 1.6756 |
| 18217524 | FBtr0307050 // Muc | Muc4B | FBtr0307050 | FBtr0307050 | 1.45E-08 | 0.0800003 | -12.4999 |
| 18192125 | NR_048409 // mir-2 | mir-2494 | NR_048409 | FBtr0304304 | 1.49E-08 | 0.138786 | -7.20535 |
| 18196255 | FBtr0082101 // Fst / | Fst | FBtr0082101 | FBtr0082101 | 1.68E-08 | 0.52917 | -1.88975 |
| 18148066 | NM_080089 // Cyp4 | Cyp4p1 | NM_080089 | FBtr0088592 | 1.86E-08 | 0.603665 | -1.65655 |
| 18131649 | FBtr0301665 // Try2 | Try29F | FBtr0301665 | FBtr0301665 | 2.00E-08 | 0.306556 | -3.26205 |
| 18214734 | FBtr0071240 // CG1 | CG12116 | FBtr0071240 | FBtr0071240 | 2.19E-08 | 0.617279 | -1.62001 |
| 18188807 | FBtr0085307 // CG9 | CG9989 | FBtr0085307 | FBtr0085307 | 2.33E-08 | 0.268789 | -3.72039 |
| 18147689 | FBtr0088122 // beta | betaTry | FBtr0088122 | FBtr0088122 | 2.38E-08 | 0.392925 | -2.54501 |
| 18177459 | NM_140194 // Plod | Plod | NM_140194 | FBtr0076187 | 2.38E-08 | 1.77913 | 1.77913 |
| 18200456 | FBtr0290201 // CG1 | CG15553 | FBtr0290201 | FBtr0290201 | 2.61E-08 | 0.315148 | -3.17311 |
| 18199455 | FBtr0113285 // CG9 | CG9996 | FBtr0113285 | FBtr0113285 | 2.88E-08 | 0.443612 | -2.25422 |
| 18135978 | FBtr0308065 // lecti | lectin-28C | FBtr0308065 | FBtr0308065 | 2.94E-08 | 0.124605 | -8.02539 |
| 18144930 | FBtr0080726 // CG3 | CG31832 | FBtr0080726 | FBtr0080726 | 3.01E-08 | 0.326918 | -3.05887 |
| 18179653 | NM_079225 // Prat2 | Prat2 | NM_079225 | FBtr0076945 | 3.45E-08 | 1.73473 | 1.73473 |
| 18187075 | FBtr0083780 // CG1 | CG16727 | FBtr0083780 | FBtr0083780 | 3.50E-08 | 0.46746 | -2.13922 |
| 18212955 | FBtr0071472 // Gip / | Gip | FBtr0071472 | FBtr0071472 | 3.54E-08 | 0.630396 | -1.5863 |
| 18144661 | FBtr0079207 // CG3 | CG31639 | FBtr0079207 | FBtr0079207 | 3.60E-08 | 0.463039 | -2.15964 |
| 18180657 | NR_003860 // snoR | snoRNA:Psi28S-1837b | NR_003860 | FBtr0113608 | 3.64E-08 | 2.8892 | 2.8892 |
| 18162032 | FBtr0072196 // CG3 | CG3907 | FBtr0072196 | FBtr0072196 | 3.70E-08 | 0.657849 | -1.5201 |
| 18145131 | NM_175946 // NLaz | NLaz | NM_175946 | FBtr0077942 | 3.82E-08 | 0.564517 | -1.77143 |
| 18174614 | NM_079381 // Pdh / | Pdh | NM_079381 | FBtr0075426 | 3.84E-08 | 1.59935 | 1.59935 |
| 18157232 | FBtr0087080 // Acp5 | Acp53Ea | FBtr0087080 | FBtr0087080 | 3.98E-08 | 0.621471 | -1.60908 |
| 18157154 | FBtr0089056 // Cyp9 | Cyp9b1 | FBtr0089056 | FBtr0089056 | 3.98E-08 | 0.163384 | -6.12056 |
| 18170952 | NM_144453 // Nplp | Nplp3 | NM_144453 | FBtr0075369 | 4.13E-08 | 2.1428 | 2.1428 |
| 18159265 | FBtr0310033 // CG1 | CG1888 | FBtr0310033 | FBtr0310033 | 4.15E-08 | 0.0756779 | -13.2139 |
| 18154273 | NM_137480 // GstE | GstE2 | NM_137480 | FBtr0086670 | 4.46E-08 | 2.59048 | 2.59048 |
| 18217333 | FBtr0073474 // CG3 | CG32667 | FBtr0073474 | FBtr0073474 | 4.47E-08 | 1.52353 | 1.52353 |
| 18140474 | FBtr0079900 // CG5 | CG5885 | FBtr0079900 | FBtr0079900 | 4.50E-08 | 0.578769 | -1.72781 |
| 18197227 | FBtr0082789 // CG1 | CG14369 | FBtr0082789 | FBtr0082789 | 4.68E-08 | 0.137221 | -7.28749 |
| 18185261 | NM_141592 // CAHb | CAHbeta | NM_141592 | FBtr0081958 | 4.80E-08 | 1.86988 | 1.86988 |
| 18149383 | FBtr0088923 // CG2 | CG2064 | FBtr0088923 | FBtr0088923 | 4.94E-08 | 3.5792 | 3.5792 |
| 18205607 | FBtr0073651 // Lsp1 | Lsp1alpha | FBtr0073651 | FBtr0073651 | 5.12E-08 | 4.91636 | 4.91636 |
| 18212963 | NM_078702 // Ser6 | Ser6 | NM_078702 | FBtr0077272 | 5.30E-08 | 0.169338 | -5.90535 |
| 18175717 | FBtr0072599 // CG1 | CG13898 | FBtr0072599 | FBtr0072599 | 5.40E-08 | 1.73139 | 1.73139 |
| 18209643 | FBtr0074631 // CG6 | CG6891 | FBtr0074631 | FBtr0074631 | 5.61E-08 | 1.78883 | 1.78883 |
| 18161556 | FBtr0071739 // CG4 | CG4377 | FBtr0071739 | FBtr0071739 | 6.08E-08 | 0.636368 | -1.57142 |
| 18154281 | NM_079076 // Act57 | Act57B | NM_079076 | FBtr0071519 | 6.45E-08 | 2.02707 | 2.02707 |
| 18169001 | FBtr0310535 // CG1 | CG14125 | FBtr0310535 | FBtr0310535 | 6.55E-08 | 0.575336 | -1.73811 |
| 18171391 | FBtr0073344 // CG3 | CG32238 | FBtr0073344 | FBtr0073344 | 6.56E-08 | 1.98044 | 1.98044 |
| 18167427 | FBtr0072979 // CG2 | CG2113 | FBtr0072979 | FBtr0072979 | 6.78E-08 | 0.666521 | -1.50033 |
| 18208265 | NM_167184 // Obp8 | Obp8a | NM_167184 | FBtr0071307 | 7.33E-08 | 0.284285 | -3.5176 |
| 18168689 | FBtr0076389 // CG1 | CG18180 | FBtr0076389 | FBtr0076389 | 7.40E-08 | 3.13145 | 3.13145 |
| 18171545 | FBtr0076749 // CG3 | CG32368 | FBtr0076749 | FBtr0076749 | 7.46E-08 | 2.48567 | 2.48567 |
| 18164897 | NR_048168 // CR43 | CR43421 | NR_048168 | FBtr0308772 | 7.84E-08 | 3.22565 | 3.22565 |
| 18208904 | FBtr0073811 // CG1 | CG11162 | FBtr0073811 | FBtr0073811 | 7.85E-08 | 0.568601 | -1.7587 |
| 18186819 | FBtr0331346 // CG1 | CG14322 | FBtr0331346 | FBtr0331346 | 7.92E-08 | 2.18492 | 2.18492 |
| 18175633 | FBtr0072574 // mthl | mthl9 | FBtr0072574 | FBtr0072574 | 8.03E-08 | 0.25475 | -3.92542 |
| 18131750 | FBtr0081201 // Lecti | Lectin-galC1 | FBtr0081201 | FBtr0081201 | 8.21E-08 | 2.23212 | 2.23212 |
| 18165909 | FBtr0073073 // Drs / | Drs | FBtr0073073 | FBtr0073073 | 8.65E-08 | 0.613342 | -1.63041 |
| 18135428 | FBtr0081068 // CG1 | CG10178 | FBtr0081068 | FBtr0081068 | 8.99E-08 | 1.56529 | 1.56529 |
| 18141540 | FBtr0077549 // CG3 | CG3513 | FBtr0077549 | FBtr0077549 | 9.29E-08 | 2.08458 | 2.08458 |
| 18141290 | FBtr0330667 // CG3 | CG3597 | FBtr0330667 | FBtr0330667 | 9.38E-08 | 0.307441 | -3.25265 |
| 18140864 | FBtr0080606 // CG1 | CG18095 | FBtr0080606 | FBtr0080606 | 1.09E-07 | 0.542454 | -1.84347 |
| 18150198 | FBtr0333061 // CG1 | CG18343 | FBtr0333061 | FBtr0333061 | 1.14E-07 | 0.604258 | -1.65492 |
| 18205966 | FBtr0071419 // Yp1 / | Yp1 | FBtr0071419 | FBtr0071419 | 1.15E-07 | 0.227698 | -4.39178 |
| 18189615 | FBtr0303151 // CG1 | CG18754 | FBtr0303151 | FBtr0303151 | 1.16E-07 | 30.7531 | 30.7531 |
| 18216750 | FBtr0070879 // vani | vanin-like | FBtr0070879 | FBtr0070879 | 1.21E-07 | 0.31887 | -3.13607 |
| 18168685 | FBtr0076387 // CG8 | CG8329 | FBtr0076387 | FBtr0076387 | 1.22E-07 | 2.76783 | 2.76783 |
| 18161336 | FBtr0086249 // CG1 | CG16799 | FBtr0086249 | FBtr0086249 | 1.28E-07 | 1.59889 | 1.59889 |
| 18133047 | FBtr0308697 // CG4 | CG4896 | FBtr0308697 | FBtr0308697 | 1.29E-07 | 1.57807 | 1.57807 |
| 18202944 | FBtr0301941 // Sfp9 | Sfp96F | FBtr0301941 | FBtr0301941 | 1.36E-07 | 0.594144 | -1.68309 |
| 18151370 | FBtr0086752 // CG1 | CG14500 | FBtr0086752 | FBtr0086752 | 1.39E-07 | 3.07131 | 3.07131 |
| 18137487 | FBtr0112368 // CG3 | CG34177 | FBtr0112368 | FBtr0112368 | 1.39E-07 | 1.73038 | 1.73038 |
| 18161086 | NM_137543 // Jheh3 | Jheh3 | NM_137543 | FBtr0086622 | 1.41E-07 | 0.546825 | -1.82874 |
| 18210519 | FBtr0073716 // CG3 | CG32643 | FBtr0073716 | FBtr0073716 | 1.48E-07 | 1.5654 | 1.5654 |
| 18154653 | FBtr0112765 // CG3 | CG34459 | FBtr0112765 | FBtr0112765 | 1.49E-07 | 0.614262 | -1.62797 |
| 18173370 | NR_048294 // CR43 | CR43306 | NR_048294 | FBtr0306840 | 1.49E-07 | 2.81639 | 2.81639 |
| 18197727 | FBtr0300677 // CG1 | CG10317 | FBtr0300677 | FBtr0300677 | 1.52E-07 | 1.64487 | 1.64487 |
| 18176029 | FBtr0072967 // Cyp4 | Cyp4d20 | FBtr0072967 | FBtr0072967 | 1.52E-07 | 0.385074 | -2.5969 |
| 18135842 | FBtr0273404 // Tsp3 | Tsp39D | FBtr0273404 | FBtr0273404 | 1.58E-07 | 1.93741 | 1.93741 |
| 18151449 | FBtr0086706 // CG5 | CG5323 | FBtr0086706 | FBtr0086706 | 1.60E-07 | 1.6319 | 1.6319 |
| 18131170 | NM_057431 // Uro / | Uro | NM_057431 | FBtr0079486 | 1.61E-07 | 1.60698 | 1.60698 |
| 18141173 | FBtr0077934 // CG1 | CG18131 | FBtr0077934 | FBtr0077934 | 1.67E-07 | 1.61258 | 1.61258 |
| 18161878 | NM_079098 // Or59 | Or59b | NM_079098 | FBtr0072021 | 1.73E-07 | 3.56877 | 3.56877 |
| 18189622 | FBtr0091720 // CG3 | CG33722 | FBtr0091720 | FBtr0091720 | 1.75E-07 | 1.70353 | 1.70353 |
| 18174628 | FBtr0334100 // Gem | Gem3 | FBtr0334100 | FBtr0334100 | 1.77E-07 | 1.91829 | 1.91829 |
| 18156905 | FBtr0072101 // l(2)e | l(2)efl | FBtr0072101 | FBtr0072101 | 1.79E-07 | 1.93023 | 1.93023 |
| 18202299 | FBtr0112499 // CG3 | CG34303 | FBtr0112499 | FBtr0112499 | 1.82E-07 | 0.54326 | -1.84074 |
| 18177484 | FBtr0076182 // CG6 | CG6168 | FBtr0076182 | FBtr0076182 | 1.83E-07 | 1.78827 | 1.78827 |
| 18161638 | NM_137814 // Oatp | Oatp58Db | NM_137814 | FBtr0071778 | 1.91E-07 | 0.661553 | -1.51159 |
| 18158996 | NM_136545 // Cyp6 | Cyp6a14 | NM_136545 | --- | 1.92E-07 | 2.75342 | 2.75342 |
| 18149971 | NM_136756 // Elp2 | Elp2 | NM_136756 | FBtr0088212 | 1.97E-07 | 3.1945 | 3.1945 |
| 18146450 | FBtr0303929 // CG4 | CG42844 | FBtr0303929 | FBtr0303929 | 1.99E-07 | 4.40889 | 4.40889 |
| 18177554 | FBtr0076131 // CG1 | CG11652 | FBtr0076131 | FBtr0076131 | 2.09E-07 | 2.9003 | 2.9003 |
| 18152432 | FBtr0072014 // eIF2 | eIF2B-delta | FBtr0072014 | FBtr0072014 | 2.13E-07 | 0.362864 | -2.75585 |
| 18181550 | FBtr0304649 // CG4 | CG43064 | FBtr0304649 | FBtr0304649 | 2.18E-07 | 1.9226 | 1.9226 |
| 18142708 | FBtr0332188 // CG9 | CG9525 | FBtr0332188 | FBtr0332188 | 2.26E-07 | 2.92241 | 2.92241 |
| 18176593 | FBtr0077068 // CG1 | CG10477 | FBtr0077068 | FBtr0077068 | 2.39E-07 | 1.96011 | 1.96011 |
| 18181599 | FBtr0304976 // CG4 | CG43085 | FBtr0304976 | FBtr0304976 | 2.41E-07 | 0.194833 | -5.13261 |
| 18159575 | FBtr0088191 // Liste | Listericin | FBtr0088191 | FBtr0088191 | 2.45E-07 | 0.537411 | -1.86077 |
| 18197461 | FBtr0083038 // CG6 | CG6912 | FBtr0083038 | FBtr0083038 | 2.46E-07 | 2.89247 | 2.89247 |
| 18187712 | FBtr0084340 // CG1 | CG17244 | FBtr0084340 | FBtr0084340 | 2.46E-07 | 0.582805 | -1.71584 |
| 18173880 | FBtr0076496 // Hsp2 | Hsp26 | FBtr0076496 | FBtr0076496 | 2.52E-07 | 1.75887 | 1.75887 |
| 18155112 | FBtr0301270 // CG4 | CG42566 | FBtr0301270 | FBtr0301270 | 2.55E-07 | 2.45161 | 2.45161 |
| 18190815 | NR_003908 // snoR | snoRNA:Psi28S-3405b | NR_003908 | FBtr0113589 | 2.59E-07 | 0.204789 | -4.88308 |
| 18151889 | FBtr0071520 // CG9 | CG9344 | FBtr0071520 | FBtr0071520 | 2.60E-07 | 0.602804 | -1.65891 |
| 18132407 | FBtr0079195 // Kr-h1 | Kr-h1 | FBtr0079195 | FBtr0079195 | 2.77E-07 | 0.649671 | -1.53924 |
| 18172117 | FBtr0333611 // CG3 | CG34391 | FBtr0333611 | FBtr0333611 | 2.79E-07 | 2.42418 | 2.42418 |
| 18179865 | FBtr0076147 // CG3 | CG32086 | FBtr0076147 | FBtr0076147 | 2.87E-07 | 1.6224 | 1.6224 |
| 18146521 | NR_048013 // CR43 | CR43097 | NR_048013 | FBtr0304883 | 3.05E-07 | 0.376483 | -2.65616 |
| 18158457 | FBtr0086005 // CG1 | CG11211 | FBtr0086005 | FBtr0086005 | 3.08E-07 | 0.100609 | -9.93943 |
| 18184257 | FBtr0083971 // TotA | TotA | FBtr0083971 | FBtr0083971 | 3.27E-07 | 1.61844 | 1.61844 |
| 18155614 | FBtr0304945 // CG4 | CG43114 | FBtr0304945 | FBtr0304945 | 3.29E-07 | 0.378623 | -2.64115 |
| 18176610 | FBtr0077063 // CG1 | CG10467 | FBtr0077063 | FBtr0077063 | 3.32E-07 | 0.609499 | -1.64069 |
| 18185920 | FBtr0110781 // CG1 | CG11598 | FBtr0110781 | FBtr0110781 | 3.38E-07 | 1.99128 | 1.99128 |
| 18143473 | FBtr0080474 // CG9 | CG9377 | FBtr0080474 | FBtr0080474 | 3.42E-07 | 0.176949 | -5.65136 |
| 18203550 | FBtr0309077 // CG4 | CG43441 | FBtr0309077 | FBtr0309077 | 3.53E-07 | 3.22617 | 3.22617 |
| 18206366 | NM_078507 // Spat | Spat | NM_078507 | FBtr0070913 | 3.56E-07 | 2.07087 | 2.07087 |
| 18169560 | FBtr0301031 // CG1 | CG13075 | FBtr0301031 | FBtr0301031 | 3.69E-07 | 0.507456 | -1.97062 |
| 18199604 | FBtr0084922 // CG1 | CG17196 | FBtr0084922 | FBtr0084922 | 3.78E-07 | 1.7469 | 1.7469 |
| 18144513 | NM_001014457 // O | Obp22a | NM_001014457 | FBtr0100503 | 3.81E-07 | 2.75106 | 2.75106 |
| 18154453 | FBtr0112420 // CG3 | CG34227 | FBtr0112420 | FBtr0112420 | 3.93E-07 | 2.14231 | 2.14231 |
| 18186079 | FBtr0082734 // CG8 | CG8141 | FBtr0082734 | FBtr0082734 | 3.98E-07 | 2.45469 | 2.45469 |
| 18187155 | FBtr0083823 // CG4 | CG4783 | FBtr0083823 | FBtr0083823 | 4.06E-07 | 0.434231 | -2.30292 |
| 18184925 | FBtr0081637 // CG1 | CG1943 | FBtr0081637 | FBtr0081637 | 4.07E-07 | 1.50594 | 1.50594 |
| 18132911 | NM_080359 // nAcR | nAcRbeta-21C | NM_080359 | FBtr0078064 | 4.13E-07 | 1.98201 | 1.98201 |
| 18208389 | FBtr0073378 // CG1 | CG15209 | FBtr0073378 | FBtr0073378 | 4.14E-07 | 0.395275 | -2.52989 |
| 18185797 | NM_169429 // HisCl | HisCl1 | NM_169429 | FBtr0082470 | 4.22E-07 | 0.320631 | -3.11885 |
| 18200943 | NM_001202266 // Pi | Pif1A | NM_001202266 | FBtr0332501 | 4.57E-07 | 0.624924 | -1.60019 |
| 18138595 | NR_047877 // mir-4 | mir-4914 | NR_047877 | FBtr0309709 | 4.57E-07 | 0.423733 | -2.35998 |
| 18194887 | FBtr0305991 // CRE | CREG | FBtr0305991 | FBtr0305991 | 4.61E-07 | 0.549824 | -1.81876 |
| 18161038 | FBtr0086633 // CG1 | CG15096 | FBtr0086633 | FBtr0086633 | 4.83E-07 | 0.63139 | -1.58381 |
| 18134568 | FBtr0079864 // CG1 | CG13117 | FBtr0079864 | FBtr0079864 | 4.93E-07 | 1.84304 | 1.84304 |
| 18163019 | FBtr0071621 // CG3 | CG30391 | FBtr0071621 | FBtr0071621 | 4.96E-07 | 1.67224 | 1.67224 |
| 18187580 | FBtr0084254 // CG7 | CG7054 | FBtr0084254 | FBtr0084254 | 5.41E-07 | 0.252771 | -3.95615 |
| 18184996 | FBtr0081739 // CG1 | CG10919 | FBtr0081739 | FBtr0081739 | 5.45E-07 | 1.93686 | 1.93686 |
| 18190740 | FBtr0091720 // CG3 | CG33722 | FBtr0091720 | FBtr0091720 | 5.63E-07 | 1.68693 | 1.68693 |
| 18153181 | NM_166394 // Obp5 | Obp57b | NM_166394 | FBtr0086293 | 5.80E-07 | 2.66967 | 2.66967 |
| 18142632 | FBtr0079701 // Bace | Bace | FBtr0079701 | FBtr0079701 | 5.87E-07 | 1.81475 | 1.81475 |
| 18183287 | FBtr0082482 // Hsp7 | Hsp70Aa | FBtr0082482 | FBtr0082482 | 5.89E-07 | 2.30349 | 2.30349 |
| 18136588 | FBtr0081422 // CG3 | CG31673 | FBtr0081422 | FBtr0081422 | 5.92E-07 | 0.610343 | -1.63842 |
| 18142588 | NM_135360 // Ostg | Ostgamma | NM_135360 | FBtr0079613 | 6.10E-07 | 1.52086 | 1.52086 |
| 18159997 | FBtr0087796 // CG1 | CG13324 | FBtr0087796 | FBtr0087796 | 6.10E-07 | 6.53883 | 6.53883 |
| 18144307 | FBtr0077539 // lecti | lectin-24A | FBtr0077539 | FBtr0077539 | 6.54E-07 | 3.10711 | 3.10711 |
| 18136172 | FBtr0081351 // CG1 | CG12617 | FBtr0081351 | FBtr0081351 | 6.65E-07 | 2.43249 | 2.43249 |
| 18135799 | FBtr0332034 // CG9 | CG9259 | FBtr0332034 | FBtr0332034 | 6.66E-07 | 0.541482 | -1.84678 |
| 18183289 | FBtr0082637 // Hsp7 | Hsp70Bc | FBtr0082637 | FBtr0082637 | 6.76E-07 | 2.76159 | 2.76159 |
| 18201624 | FBtr0084918 // CG3 | CG31380 | FBtr0084918 | FBtr0084918 | 6.79E-07 | 0.408092 | -2.45043 |
| 18192535 | NR_048466 // CR43 | CR43475 | NR_048466 | FBtr0309305 | 6.93E-07 | 0.225968 | -4.42541 |
| 18213397 | FBtr0070423 // CG1 | CG18031 | FBtr0070423 | FBtr0070423 | 7.06E-07 | 0.407878 | -2.45171 |
| 18151514 | NM_079063 // DptB | DptB | NM_079063 | FBtr0086621 | 7.26E-07 | 0.40833 | -2.449 |
| 18193862 | NM_057582 // Nmd | Nmdmc | NM_057582 | FBtr0081997 | 7.28E-07 | 1.61228 | 1.61228 |
| 18181486 | FBtr0303833 // CG4 | CG42825 | FBtr0303833 | FBtr0303833 | 7.31E-07 | 0.555797 | -1.79922 |
| 18193043 | NM_058155 // Gld / | Gld | NM_058155 | FBtr0081596 | 7.37E-07 | 1.74217 | 1.74217 |
| 18200425 | FBtr0085722 // Npc2 | Npc2h | FBtr0085722 | FBtr0085722 | 7.65E-07 | 1.56955 | 1.56955 |
| 18215898 | FBtr0074287 // CG9 | CG9777 | FBtr0074287 | FBtr0074287 | 7.77E-07 | 1.57854 | 1.57854 |
| 18187011 | FBtr0083755 // CG3 | CG3734 | FBtr0083755 | FBtr0083755 | 7.86E-07 | 0.563479 | -1.77469 |
| 18173072 | FBtr0304017 // CG4 | CG42852 | FBtr0304017 | FBtr0304017 | 7.86E-07 | 0.509885 | -1.96123 |
| 18202554 | NM_001260430 // D | Dup99B | NM_001260430 | FBtr0305053 | 7.88E-07 | 2.58703 | 2.58703 |
| 18160078 | FBtr0087721 // CG1 | CG17048 | FBtr0087721 | FBtr0087721 | 8.03E-07 | 2.05804 | 2.05804 |
| 18199587 | NM_170237 // CLS / | CLS | NM_170237 | FBtr0084929 | 8.08E-07 | 1.73227 | 1.73227 |
| 18172044 | FBtr0112436 // CG3 | CG34242 | FBtr0112436 | FBtr0112436 | 8.14E-07 | 1.81621 | 1.81621 |
| 18159541 | FBtr0088211 // Spn4 | Spn47C | FBtr0088211 | FBtr0088211 | 8.19E-07 | 0.611742 | -1.63468 |
| 18140949 | FBtr0079624 // Spn2 | Spn28F | FBtr0079624 | FBtr0079624 | 8.38E-07 | 0.362246 | -2.76055 |
| 18155662 | FBtr0306049 // CG4 | CG43188 | FBtr0306049 | FBtr0306049 | 8.50E-07 | 0.244757 | -4.08569 |
| 18190299 | FBtr0082636 // Hsp7 | Hsp70Bbb | FBtr0082636 | FBtr0082636 | 8.56E-07 | 3.59462 | 3.59462 |
| 18157486 | FBtr0087117 // Amyr | Amyrel | FBtr0087117 | FBtr0087117 | 8.60E-07 | 0.211762 | -4.72228 |
| 18144052 | FBtr0081350 // CG1 | CG10659 | FBtr0081350 | FBtr0081350 | 8.67E-07 | 1.91888 | 1.91888 |
| 18216382 | NM_001103561 // C | CG8028 | NM_001103561 | FBtr0332393 | 8.70E-07 | 0.294934 | -3.39058 |
| 18167122 | FBtr0072692 // CG9 | CG9168 | FBtr0072692 | FBtr0072692 | 8.73E-07 | 2.78849 | 2.78849 |
| 18217940 | FBtr0310439 // spri / | spri | FBtr0310439 | FBtr0310439 | 8.74E-07 | 0.567929 | -1.76078 |
| 18209071 | FBtr0074035 // CG1 | CG15641 | FBtr0074035 | FBtr0074035 | 8.78E-07 | 0.485237 | -2.06085 |
| 18189219 | FBtr0085655 // CG1 | CG15539 | FBtr0085655 | FBtr0085655 | 8.79E-07 | 0.333686 | -2.99683 |
| 18144431 | FBtr0077748 // Cyp3 | Cyp309a2 | FBtr0077748 | FBtr0077748 | 8.98E-07 | 0.296817 | -3.36908 |
| 18178875 | FBtr0333845 // CG9 | CG9451 | FBtr0333845 | FBtr0333845 | 9.00E-07 | 0.482967 | -2.07053 |
| 18141968 | FBtr0302213 // CG9 | CG9150 | FBtr0302213 | FBtr0302213 | 1.00E-06 | 2.09883 | 2.09883 |
| 18164663 | FBtr0304891 // CG4 | CG43103 | FBtr0304891 | FBtr0304891 | 1.02E-06 | 2.07989 | 2.07989 |
| 18197578 | FBtr0083106 // CG6 | CG6125 | FBtr0083106 | FBtr0083106 | 1.04E-06 | 0.413555 | -2.41806 |
| 18202995 | FBtr0084894 // ymp | ymp | FBtr0084894 | FBtr0084894 | 1.05E-06 | 1.55256 | 1.55256 |
| 18154271 | NM_137481 // GstE | GstE3 | NM_137481 | FBtr0086671 | 1.06E-06 | 1.61363 | 1.61363 |
| 18185346 | FBtr0082025 // CG1 | CG18473 | FBtr0082025 | FBtr0082025 | 1.08E-06 | 1.72964 | 1.72964 |
| 18190597 | NM_176518 // MtnD | MtnD | NM_176518 | FBtr0308064 | 1.10E-06 | 0.618579 | -1.61661 |
| 18190017 | FBtr0084441 // CG3 | CG31148 | FBtr0084441 | FBtr0084441 | 1.13E-06 | 0.488896 | -2.04542 |
| 18136867 | FBtr0300460 // CG3 | CG31909 | FBtr0300460 | FBtr0300460 | 1.15E-06 | 0.443866 | -2.25293 |
| 18214944 | FBtr0071512 // CG1 | CG15296 | FBtr0071512 | FBtr0071512 | 1.18E-06 | 0.468484 | -2.13454 |
| 18185768 | FBtr0082423 // CG1 | CG14715 | FBtr0082423 | FBtr0082423 | 1.24E-06 | 0.571803 | -1.74885 |
| 18190365 | FBtr0083432 // CG3 | CG31418 | FBtr0083432 | FBtr0083432 | 1.26E-06 | 0.618536 | -1.61672 |
| 18154251 | NR_001756 // snoR | snoRNA:Me28S-A3407a | NR_001756 | FBtr0086844 | 1.27E-06 | 0.546551 | -1.82966 |
| 18193419 | FBtr0085512 // Jon9 | Jon99Cii | FBtr0085512 | FBtr0085512 | 1.27E-06 | 3.34289 | 3.34289 |
| 18187099 | FBtr0083791 // CG1 | CG11453 | FBtr0083791 | FBtr0083791 | 1.32E-06 | 0.581273 | -1.72036 |
| 18132521 | FBtr0080549 // CG7 | CG7916 | FBtr0080549 | FBtr0080549 | 1.34E-06 | 0.476138 | -2.10023 |
| 18180949 | NM_168477 // CG42 | CG42255 | NM_168477 | FBtr0332131 | 1.39E-06 | 0.364325 | -2.74481 |
| 18138545 | NR_047960 // CR43 | CR43412 | NR_047960 | FBtr0308754 | 1.39E-06 | 2.25519 | 2.25519 |
| 18182621 | FBtr0085502 // Jon9 | Jon99Cii | FBtr0085502 | FBtr0085502 | 1.39E-06 | 2.80188 | 2.80188 |
| 18149232 | FBtr0089030 // Tsp4 | Tsp42Ej | FBtr0089030 | FBtr0089030 | 1.46E-06 | 1.78248 | 1.78248 |
| 18171337 | FBtr0075174 // edin | edin | FBtr0075174 | FBtr0075174 | 1.48E-06 | 0.292106 | -3.42342 |
| 18217842 | NR_003729 // snoR | snoRNA:Psi28S-1060 | NR_003729 | FBtr0113560 | 1.50E-06 | 1.52543 | 1.52543 |
| 18177135 | FBtr0076559 // CG5 | CG5653 | FBtr0076559 | FBtr0076559 | 1.55E-06 | 0.320779 | -3.11741 |
| 18134080 | FBtr0079439 // CG5 | CG5171 | FBtr0079439 | FBtr0079439 | 1.57E-06 | 0.508265 | -1.96748 |
| 18137009 | NM_001103597 // C | CG33282 | NM_001103597 | FBtr0113458 | 1.77E-06 | 0.470211 | -2.1267 |
| 18156473 | FBtr0088161 // alph | alphaTry | FBtr0088161 | FBtr0088161 | 1.80E-06 | 1.5284 | 1.5284 |
| 18180631 | NR_002476 // snoR | snoRNA:684 | NR_002476 | FBtr0091798 | 1.83E-06 | 0.625947 | -1.59758 |
| 18144477 | FBtr0321267 // CG1 | CG18787 | FBtr0321267 | FBtr0321267 | 1.85E-06 | 0.545439 | -1.83338 |
| 18211550 | FBtr0077239 // Npc1 | Npc1b | FBtr0077239 | FBtr0077239 | 1.87E-06 | 0.455575 | -2.19503 |
| 18167389 | FBtr0072937 // CG1 | CG1143 | FBtr0072937 | FBtr0072937 | 1.89E-06 | 0.556129 | -1.79814 |
| 18208270 | FBtr0071309 // CG1 | CG15369 | FBtr0071309 | FBtr0071309 | 1.92E-06 | 3.95393 | 3.95393 |
| 18207870 | NM_132101 // Apc7 | Apc7 | NM_132101 | FBtr0070946 | 1.94E-06 | 0.318158 | -3.14309 |
| 18186591 | FBtr0083225 // sxe2 | sxe2 | FBtr0083225 | FBtr0083225 | 1.98E-06 | 0.630211 | -1.58677 |
| 18144601 | FBtr0307507 // CG3 | CG31601 | FBtr0307507 | FBtr0307507 | 2.01E-06 | 1.8294 | 1.8294 |
| 18172197 | FBtr0112762 // CG3 | CG34456 | FBtr0112762 | FBtr0112762 | 2.05E-06 | 0.614005 | -1.62865 |
| 18131665 | FBtr0080252 // kek2 | kek2 | FBtr0080252 | FBtr0080252 | 2.13E-06 | 0.493873 | -2.02481 |
| 18216879 | FBtr0070252 // eIF4 | eIF4E-7 | FBtr0070252 | FBtr0070252 | 2.15E-06 | 0.381288 | -2.62269 |
| 18171345 | FBtr0075125 // CG3 | CG32195 | FBtr0075125 | FBtr0075125 | 2.18E-06 | 0.526733 | -1.8985 |
| 18176067 | NM_139504 // scra | scramb2 | NM_139504 | FBtr0073005 | 2.23E-06 | 1.64713 | 1.64713 |
| 18182134 | FBtr0113773 // CG4 | CG40472 | FBtr0113773 | FBtr0113773 | 2.27E-06 | 0.49498 | -2.02028 |
| 18184424 | FBtr0111222 // CG1 | CG1092 | FBtr0111222 | FBtr0111222 | 2.29E-06 | 2.48078 | 2.48078 |
| 18172039 | FBtr0112432 // CG3 | CG34238 | FBtr0112432 | FBtr0112432 | 2.31E-06 | 2.74084 | 2.74084 |
| 18190376 | FBtr0084830 // CG3 | CG31436 | FBtr0084830 | FBtr0084830 | 2.31E-06 | 0.568183 | -1.76 |
| 18188796 | FBtr0085290 // CG1 | CG1894 | FBtr0085290 | FBtr0085290 | 2.44E-06 | 0.295695 | -3.38187 |
| 18141318 | FBtr0077749 // Cyp3 | Cyp309a1 | FBtr0077749 | FBtr0077749 | 2.46E-06 | 2.20483 | 2.20483 |
| 18216626 | FBtr0077271 // CG1 | CG1304 | FBtr0077271 | FBtr0077271 | 2.49E-06 | 0.210703 | -4.74602 |
| 18162258 | FBtr0072411 // Tina- | Tina-1 | FBtr0072411 | FBtr0072411 | 2.53E-06 | 0.633345 | -1.57892 |
| 18144305 | FBtr0079715 // lecti | lectin-29Ca | FBtr0079715 | FBtr0079715 | 2.56E-06 | 0.576669 | -1.7341 |
| 18137060 | FBtr0091498 // lecti | lectin-37Da | FBtr0091498 | FBtr0091498 | 2.65E-06 | 2.39951 | 2.39951 |
| 18187611 | NM_001170219 // o | oa2 | NM_001170219 | FBtr0301484 | 2.70E-06 | 1.84107 | 1.84107 |
| 18164668 | FBtr0304889 // CG4 | CG43106 | FBtr0304889 | FBtr0304889 | 2.72E-06 | 2.27695 | 2.27695 |
| 18176026 | FBtr0072968 // CG1 | CG16762 | FBtr0072968 | FBtr0072968 | 2.75E-06 | 0.543905 | -1.83856 |
| 18162951 | FBtr0088798 // CG3 | CG30371 | FBtr0088798 | FBtr0088798 | 2.77E-06 | 0.630414 | -1.58626 |
| 18169554 | FBtr0075437 // CG5 | CG5895 | FBtr0075437 | FBtr0075437 | 2.92E-06 | 0.525298 | -1.90368 |
| 18200433 | FBtr0085718 // CG1 | CG15546 | FBtr0085718 | FBtr0085718 | 2.95E-06 | 0.368697 | -2.71225 |
| 18186676 | FBtr0083329 // CG1 | CG17562 | FBtr0083329 | FBtr0083329 | 2.98E-06 | 0.607597 | -1.64583 |
| 18179789 | FBtr0114525 // CG3 | CG32036 | FBtr0114525 | FBtr0114525 | 3.04E-06 | 2.50897 | 2.50897 |
| 18133640 | FBtr0332476 // CG1 | CG12194 | FBtr0332476 | FBtr0332476 | 3.06E-06 | 1.50639 | 1.50639 |
| 18165226 | FBtr0308893 // CG4 | CG40498 | FBtr0308893 | FBtr0308893 | 3.11E-06 | 1.8237 | 1.8237 |
| 18171092 | FBtr0076567 // Cpr6 | Cpr66D | FBtr0076567 | FBtr0076567 | 3.22E-06 | 2.05593 | 2.05593 |
| 18201661 | FBtr0332431 // CG3 | CG31445 | FBtr0332431 | FBtr0332431 | 3.32E-06 | 1.70022 | 1.70022 |
| 18189936 | FBtr0084941 // CG3 | CG31091 | FBtr0084941 | FBtr0084941 | 3.37E-06 | 2.48178 | 2.48178 |
| 18180485 | FBtr0089526 // CG3 | CG33490 | FBtr0089526 | FBtr0089526 | 3.43E-06 | 2.13992 | 2.13992 |
| 18190328 | FBtr0290055 // CG3 | CG31370 | FBtr0290055 | FBtr0290055 | 3.48E-06 | 0.522502 | -1.91387 |
| 18208608 | FBtr0073569 // CG1 | CG15739 | FBtr0073569 | FBtr0073569 | 3.51E-06 | 0.572161 | -1.74776 |
| 18203391 | FBtr0305707 // CG4 | CG43166 | FBtr0305707 | FBtr0305707 | 3.59E-06 | 0.15851 | -6.30876 |
| 18168879 | FBtr0076173 // CG7 | CG7560 | FBtr0076173 | FBtr0076173 | 3.61E-06 | 1.80944 | 1.80944 |
| 18198450 | FBtr0083894 // CG4 | CG4335 | FBtr0083894 | FBtr0083894 | 3.63E-06 | 2.3734 | 2.3734 |
| 18151197 | FBtr0086916 // CG4 | CG4802 | FBtr0086916 | FBtr0086916 | 3.64E-06 | 1.57375 | 1.57375 |
| 18190088 | FBtr0084505 // LSm3 | LSm3 | FBtr0084505 | FBtr0084505 | 3.72E-06 | 2.03003 | 2.03003 |
| 18163701 | FBtr0112409 // CG3 | CG34216 | FBtr0112409 | FBtr0112409 | 3.75E-06 | 3.11319 | 3.11319 |
| 18151186 | NM_137350 // Gbp / | Gbp | NM_137350 | FBtr0086986 | 3.77E-06 | 2.07699 | 2.07699 |
| 18178958 | FBtr0333878 // CG1 | CG14103 | FBtr0333878 | FBtr0333878 | 3.81E-06 | 0.647123 | -1.5453 |
| 18202102 | FBtr0083346 // CG3 | CG31275 | FBtr0083346 | FBtr0083346 | 3.81E-06 | 0.514989 | -1.94179 |
| 18143697 | FBtr0080989 // CG6 | CG6870 | FBtr0080989 | FBtr0080989 | 3.88E-06 | 1.67018 | 1.67018 |
| 18188471 | FBtr0273389 // CG1 | CG14556 | FBtr0273389 | FBtr0273389 | 3.94E-06 | 0.604301 | -1.65481 |
| 18198056 | FBtr0083600 // CG7 | CG7142 | FBtr0083600 | FBtr0083600 | 3.97E-06 | 2.09303 | 2.09303 |
| 18168283 | FBtr0076807 // PGR | PGRP-SD | FBtr0076807 | FBtr0076807 | 4.01E-06 | 0.65958 | -1.51612 |
| 18140870 | FBtr0080685 // CG1 | CG15279 | FBtr0080685 | FBtr0080685 | 4.03E-06 | 0.589899 | -1.6952 |
| 18199503 | FBtr0084864 // CG1 | CG10514 | FBtr0084864 | FBtr0084864 | 4.04E-06 | 1.54809 | 1.54809 |
| 18165424 | FBtr0076454 // Hsp2 | Hsp27 | FBtr0076454 | FBtr0076454 | 4.09E-06 | 1.56682 | 1.56682 |
| 18150304 | FBtr0087922 // Cpr4 | Cpr49Ag | FBtr0087922 | FBtr0087922 | 4.11E-06 | 3.75256 | 3.75256 |
| 18207985 | FBtr0071013 // CG4 | CG4607 | FBtr0071013 | FBtr0071013 | 4.17E-06 | 0.618472 | -1.61689 |
| 18139278 | NM_057296 // Acp2 | Acp26Aa | NM_057296 | FBtr0079155 | 4.27E-06 | 0.515986 | -1.93804 |
| 18180712 | FBtr0302318 // CG3 | CG34256 | FBtr0302318 | FBtr0302318 | 4.42E-06 | 1.50204 | 1.50204 |
| 18207981 | FBtr0071009 // CG4 | CG4593 | FBtr0071009 | FBtr0071009 | 4.43E-06 | 4.35977 | 4.35977 |
| 18186402 | FBtr0083026 // CG3 | CG3987 | FBtr0083026 | FBtr0083026 | 4.48E-06 | 1.63971 | 1.63971 |
| 18134997 | NM_135678 // Mal- | Mal-B1 | NM_135678 | FBtr0080275 | 4.52E-06 | 0.562827 | -1.77675 |
| 18194195 | FBtr0082679 // Hsp7 | Hsp70Ba | FBtr0082679 | FBtr0082679 | 4.91E-06 | 2.97245 | 2.97245 |
| 18216699 | FBtr0077219 // CG1 | CG1678 | FBtr0077219 | FBtr0077219 | 5.01E-06 | 0.575815 | -1.73667 |
| 18144828 | FBtr0077509 // CG3 | CG31773 | FBtr0077509 | FBtr0077509 | 5.07E-06 | 1.55827 | 1.55827 |
| 18154314 | FBtr0300861 // Nop6 | Nop60B | FBtr0300861 | FBtr0300861 | 5.17E-06 | 0.356273 | -2.80684 |
| 18154348 | NR_003829 // snoR | snoRNA:Psi28S-1175c | NR_003829 | FBtr0113529 | 5.24E-06 | 1.76006 | 1.76006 |
| 18185067 | FBtr0113204 // CG7 | CG7900 | FBtr0113204 | FBtr0113204 | 5.31E-06 | 0.620416 | -1.61182 |
| 18145744 | FBtr0079266 // Sec6 | Sec61alpha | FBtr0079266 | FBtr0079266 | 5.56E-06 | 0.625744 | -1.5981 |
| 18195572 | FBtr0078796 // CG2 | CG2663 | FBtr0078796 | FBtr0078796 | 5.69E-06 | 2.37455 | 2.37455 |
| 18175762 | FBtr0072635 // CG9 | CG9119 | FBtr0072635 | FBtr0072635 | 5.70E-06 | 0.570226 | -1.75369 |
| 18206881 | FBtr0077242 // Mgst | Mgstl | FBtr0077242 | FBtr0077242 | 5.83E-06 | 0.537492 | -1.86049 |
| 18208761 | FBtr0073721 // CG2 | CG2200 | FBtr0073721 | FBtr0073721 | 5.88E-06 | 1.73644 | 1.73644 |
| 18196234 | FBtr0082111 // CG9 | CG9396 | FBtr0082111 | FBtr0082111 | 5.89E-06 | 0.637023 | -1.5698 |
| 18187205 | FBtr0083884 // Mtn | MtnC | FBtr0083884 | FBtr0083884 | 5.94E-06 | 1.64437 | 1.64437 |
| 18143158 | FBtr0114455 // Ast- | Ast-CC | FBtr0114455 | FBtr0114455 | 6.09E-06 | 2.55982 | 2.55982 |
| 18175136 | FBtr0073279 // wit / | wit | FBtr0073279 | FBtr0073279 | 6.10E-06 | 0.665118 | -1.50349 |
| 18165422 | FBtr0076453 // Hsp2 | Hsp23 | FBtr0076453 | FBtr0076453 | 6.13E-06 | 1.63326 | 1.63326 |
| 18217428 | FBtr0273437 // CG3 | CG32702 | FBtr0273437 | FBtr0273437 | 6.16E-06 | 0.568242 | -1.75981 |
| 18166757 | NM_001202157 // V | Vha16-3 | NM_001202157 | FBtr0302359 | 6.37E-06 | 2.29432 | 2.29432 |
| 18187061 | FBtr0273217 // CG1 | CG17751 | FBtr0273217 | FBtr0273217 | 6.41E-06 | 0.572057 | -1.74808 |
| 18173244 | FBtr0305484 // CG4 | CG43146 | FBtr0305484 | FBtr0305484 | 6.47E-06 | 0.545112 | -1.83449 |
| 18198987 | FBtr0084431 // CG4 | CG4408 | FBtr0084431 | FBtr0084431 | 6.52E-06 | 2.37838 | 2.37838 |
| 18157091 | NM_133166 // imd / | imd | NM_133166 | FBtr0086725 | 6.68E-06 | 0.649285 | -1.54016 |
| 18134177 | FBtr0079487 // CG7 | CG7164 | FBtr0079487 | FBtr0079487 | 6.69E-06 | 1.54455 | 1.54455 |
| 18149265 | FBtr0089036 // Tsp4 | Tsp42Er | FBtr0089036 | FBtr0089036 | 6.95E-06 | 1.54746 | 1.54746 |
| 18176426 | FBtr0073362 // CG7 | CG7509 | FBtr0073362 | FBtr0073362 | 6.99E-06 | 1.60782 | 1.60782 |
| 18154607 | FBtr0301854 // CG3 | CG34423 | FBtr0301854 | FBtr0301854 | 7.05E-06 | 0.404653 | -2.47125 |
| 18153919 | FBtr0086338 // CG3 | CG30447 | FBtr0086338 | FBtr0086338 | 7.07E-06 | 1.95591 | 1.95591 |
| 18187769 | NM_001275932 // Ir | Ir | NM_001275932 | FBtr0336477 | 7.10E-06 | 0.546494 | -1.82985 |
| 18162754 | FBtr0086266 // CG3 | CG30148 | FBtr0086266 | FBtr0086266 | 7.12E-06 | 1.56478 | 1.56478 |
| 18152248 | FBtr0309799 // CG1 | CG13510 | FBtr0309799 | FBtr0309799 | 7.14E-06 | 0.607066 | -1.64727 |
| 18154245 | NR_001761 // snoR | snoRNA:U29:54Ed | NR_001761 | FBtr0086849 | 7.19E-06 | 0.60636 | -1.64919 |
| 18211915 | NR_047779 // CR43 | CR43615 | NR_047779 | FBtr0309826 | 7.19E-06 | 2.27658 | 2.27658 |
| 18207615 | FBtr0070780 // CG1 | CG12730 | FBtr0070780 | FBtr0070780 | 7.46E-06 | 1.5638 | 1.5638 |
| 18215817 | FBtr0074240 // CG9 | CG9914 | FBtr0074240 | FBtr0074240 | 7.55E-06 | 0.532777 | -1.87696 |
| 18216180 | FBtr0074585 // CG1 | CG15059 | FBtr0074585 | FBtr0074585 | 7.62E-06 | 1.60565 | 1.60565 |
| 18164218 | FBtr0087715 // CG3 | CG30059 | FBtr0087715 | FBtr0087715 | 7.64E-06 | 0.173355 | -5.7685 |
| 18153190 | FBtr0088707 // PGR | PGRP-SC1b | FBtr0088707 | FBtr0088707 | 7.65E-06 | 4.15367 | 4.15367 |
| 18210813 | FBtr0070644 // GlcA | GlcAT-I | FBtr0070644 | FBtr0070644 | 7.90E-06 | 0.51089 | -1.95737 |
| 18176849 | FBtr0076866 // CG1 | CG14835 | FBtr0076866 | FBtr0076866 | 8.09E-06 | 1.58876 | 1.58876 |
| 18188334 | FBtr0273361 // CG1 | CG13658 | FBtr0273361 | FBtr0273361 | 8.15E-06 | 1.51763 | 1.51763 |
| 18164052 | FBtr0299764 // CG4 | CG42321 | FBtr0299764 | FBtr0299764 | 8.22E-06 | 0.664098 | -1.5058 |
| 18203218 | FBtr0100008 // kibra | kibra | FBtr0100008 | FBtr0100008 | 8.30E-06 | 1.58129 | 1.58129 |
| 18135414 | FBtr0081049 // CG5 | CG5783 | FBtr0081049 | FBtr0081049 | 8.31E-06 | 1.83105 | 1.83105 |
| 18191582 | FBtr0299938 // moi / | moi | FBtr0299938 | FBtr0299938 | 8.46E-06 | 0.632375 | -1.58134 |
| 18191750 | FBtr0299938 // moi / | moi | FBtr0299938 | FBtr0299938 | 8.46E-06 | 0.632375 | -1.58134 |
| 18141034 | FBtr0078099 // CG1 | CG11912 | FBtr0078099 | FBtr0078099 | 8.74E-06 | 0.531552 | -1.88128 |
| 18203300 | FBtr0304880 // CG4 | CG43092 | FBtr0304880 | FBtr0304880 | 8.78E-06 | 0.612885 | -1.63163 |
| 18157969 | FBtr0089050 // Ady4 | Ady43A | FBtr0089050 | FBtr0089050 | 8.82E-06 | 0.516829 | -1.93488 |
| 18154267 | NM_137483 // GstE | GstE5 | NM_137483 | FBtr0086673 | 8.85E-06 | 2.06414 | 2.06414 |
| 18151714 | NM_137598 // Obp5 | Obp56a | NM_137598 | FBtr0086476 | 9.12E-06 | 0.545568 | -1.83295 |
| 18137020 | NM_205955 // CG33 | CG33301 | NM_205955 | FBtr0079935 | 9.20E-06 | 1.8061 | 1.8061 |
| 18164944 | NR_048083 // snoR | snoRNA:lola-b | NR_048083 | FBtr0309780 | 9.20E-06 | 1.78052 | 1.78052 |
| 18170858 | NM_206410 // aspar | asparagine-synthetase | NM_206410 | FBtr0089463 | 9.24E-06 | 1.93202 | 1.93202 |
| 18155109 | FBtr0301269 // CG4 | CG42565 | FBtr0301269 | FBtr0301269 | 9.29E-06 | 1.84464 | 1.84464 |
| 18135847 | FBtr0081517 // CG8 | CG8665 | FBtr0081517 | FBtr0081517 | 9.30E-06 | 1.55005 | 1.55005 |
| 18185928 | FBtr0303841 // CG1 | CG11608 | FBtr0303841 | FBtr0303841 | 9.60E-06 | 0.600783 | -1.66449 |
| 18150710 | NM_137156 // Cyp6 | Cyp6a23 | NM_137156 | FBtr0087452 | 9.69E-06 | 1.59302 | 1.59302 |
| 18153008 | FBtr0100554 // CG1 | CG13516 | FBtr0100554 | FBtr0100554 | 9.93E-06 | 1.67024 | 1.67024 |
| 18197615 | FBtr0083157 // CG4 | CG44014 | FBtr0083157 | FBtr0083157 | 9.98E-06 | 0.537317 | -1.8611 |
| 18191138 | FBtr0082181 // Inva | Invadolysin | FBtr0082181 | FBtr0082181 | 1.00E-05 | 0.560484 | -1.78417 |
| 18170639 | FBtr0332052 // CG1 | CG11131 | FBtr0332052 | FBtr0332052 | 1.01E-05 | 0.338705 | -2.95242 |
| 18136466 | FBtr0299873 // CG3 | CG31609 | FBtr0299873 | FBtr0299873 | 1.01E-05 | 1.95271 | 1.95271 |
| 18169436 | FBtr0075615 // CG3 | CG33259 | FBtr0075615 | FBtr0075615 | 1.02E-05 | 2.64926 | 2.64926 |
| 18133508 | FBtr0300299 // CG2 | CG2816 | FBtr0300299 | FBtr0300299 | 1.04E-05 | 1.88416 | 1.88416 |
| 18138451 | NR_047923 // CR43 | CR43262 | NR_047923 | FBtr0306547 | 1.06E-05 | 2.20602 | 2.20602 |
| 18217032 | FBtr0071481 // CG1 | CG12643 | FBtr0071481 | FBtr0071481 | 1.07E-05 | 1.94399 | 1.94399 |
| 18140214 | FBtr0100432 // Jon2 | Jon25Bi | FBtr0100432 | FBtr0100432 | 1.08E-05 | 1.98955 | 1.98955 |
| 18141419 | FBtr0077670 // alph | alpha4GT1 | FBtr0077670 | FBtr0077670 | 1.11E-05 | 1.84275 | 1.84275 |
| 18197593 | FBtr0083160 // CG1 | CG14869 | FBtr0083160 | FBtr0083160 | 1.13E-05 | 0.638934 | -1.56511 |
| 18142949 | FBtr0080073 // Hand | Hand | FBtr0080073 | FBtr0080073 | 1.13E-05 | 0.558241 | -1.79134 |
| 18163021 | FBtr0071618 // CG3 | CG30392 | FBtr0071618 | FBtr0071618 | 1.13E-05 | 0.427026 | -2.34178 |
| 18149657 | NM_136628 // Cyp4 | Cyp4p2 | NM_136628 | FBtr0088591 | 1.18E-05 | 0.660647 | -1.51367 |
| 18146744 | NM_001273008 // C | CG43750 | NM_001273008 | FBtr0330686 | 1.21E-05 | 2.1675 | 2.1675 |
| 18207604 | FBtr0070777 // CG1 | CG15784 | FBtr0070777 | FBtr0070777 | 1.24E-05 | 0.504883 | -1.98066 |
| 18174160 | NM_057353 // Rh4 / | Rh4 | NM_057353 | FBtr0075338 | 1.24E-05 | 1.62857 | 1.62857 |
| 18154092 | NM_206043 // CheB | CheB42a | NM_206043 | FBtr0086168 | 1.25E-05 | 1.68066 | 1.68066 |
| 18176417 | FBtr0073366 // CG1 | CG18418 | FBtr0073366 | FBtr0073366 | 1.26E-05 | 1.78211 | 1.78211 |
| 18139434 | NR_002130 // snRN | snRNA:U2:14B | NR_002130 | FBtr0074208 | 1.27E-05 | 1.64954 | 1.64954 |
| 18205954 | NR_002130 // snRN | snRNA:U2:14B | NR_002130 | FBtr0074208 | 1.27E-05 | 1.64954 | 1.64954 |
| 18199618 | NM_206574 // Ccap | CcapR | NM_206574 | FBtr0304043 | 1.28E-05 | 3.81142 | 3.81142 |
| 18148051 | FBtr0087386 // Mtk | Mtk | FBtr0087386 | FBtr0087386 | 1.29E-05 | 0.661214 | -1.51237 |
| 18168687 | FBtr0076388 // CG1 | CG18179 | FBtr0076388 | FBtr0076388 | 1.29E-05 | 0.305766 | -3.27047 |
| 18146095 | FBtr0310075 // nimC | nimC1 | FBtr0310075 | FBtr0310075 | 1.29E-05 | 1.79913 | 1.79913 |
| 18196830 | FBtr0306697 // CG1 | CG12224 | FBtr0306697 | FBtr0306697 | 1.29E-05 | 0.65458 | -1.5277 |
| 18207682 | FBtr0070819 // CG1 | CG12729 | FBtr0070819 | FBtr0070819 | 1.30E-05 | 0.590397 | -1.69378 |
| 18146244 | FBtr0333233 // Sfp2 | Sfp26Ad | FBtr0333233 | FBtr0333233 | 1.33E-05 | 1.87591 | 1.87591 |
| 18188346 | FBtr0273265 // CG1 | CG11913 | FBtr0273265 | FBtr0273265 | 1.36E-05 | 1.82645 | 1.82645 |
| 18182342 | NM_142415 // DNas | DNaseII | NM_142415 | FBtr0083538 | 1.37E-05 | 0.568082 | -1.76031 |
| 18155829 | NR_048144 // CR43 | CR43399 | NR_048144 | FBtr0308635 | 1.38E-05 | 2.116 | 2.116 |
| 18169211 | FBtr0075810 // CG1 | CG10741 | FBtr0075810 | FBtr0075810 | 1.40E-05 | 0.395049 | -2.53133 |
| 18146105 | FBtr0300296 // Sfp2 | Sfp24Ba | FBtr0300296 | FBtr0300296 | 1.41E-05 | 2.1974 | 2.1974 |
| 18155451 | FBtr0303283 // CG4 | CG42753 | FBtr0303283 | FBtr0303283 | 1.46E-05 | 1.53845 | 1.53845 |
| 18150444 | FBtr0087792 // CG1 | CG13321 | FBtr0087792 | FBtr0087792 | 1.49E-05 | 0.519802 | -1.92381 |
| 18150999 | FBtr0087137 // CG1 | CG15705 | FBtr0087137 | FBtr0087137 | 1.51E-05 | 0.569455 | -1.75606 |
| 18173514 | NR_048215 // snoR | snoRNA:CG32479-a | NR_048215 | FBtr0309760 | 1.53E-05 | 1.53182 | 1.53182 |
| 18203499 | NR_048324 // snRN | snRNA:U4atac:82E | NR_048324 | FBtr0306791 | 1.54E-05 | 0.646216 | -1.54747 |
| 18216224 | FBtr0074627 // CG1 | CG15044 | FBtr0074627 | FBtr0074627 | 1.55E-05 | 1.51853 | 1.51853 |
| 18167954 | FBtr0077086 // CG5 | CG5150 | FBtr0077086 | FBtr0077086 | 1.58E-05 | 2.37185 | 2.37185 |
| 18153548 | FBtr0071967 // CG3 | CG30192 | FBtr0071967 | FBtr0071967 | 1.58E-05 | 1.5458 | 1.5458 |
| 18188653 | NM_143307 // Gr98 | Gr98a | NM_143307 | FBtr0085184 | 1.59E-05 | 3.28236 | 3.28236 |
| 18153511 | FBtr0086215 // CG3 | CG30154 | FBtr0086215 | FBtr0086215 | 1.60E-05 | 0.411793 | -2.4284 |
| 18177323 | FBtr0113156 // CG6 | CG6709 | FBtr0113156 | FBtr0113156 | 1.62E-05 | 1.51342 | 1.51342 |
| 18177330 | FBtr0113156 // CG6 | CG6709 | FBtr0113156 | FBtr0113156 | 1.62E-05 | 1.51342 | 1.51342 |
| 18138773 | NR_073787 // CR43 | CR43825 | NR_073787 | FBtr0332227 | 1.62E-05 | 2.58823 | 2.58823 |
| 18183063 | NM_176479 // GstD | GstD3 | NM_176479 | FBtr0082570 | 1.63E-05 | 1.73324 | 1.73324 |
| 18133081 | FBtr0077920 // CG5 | CG5397 | FBtr0077920 | FBtr0077920 | 1.63E-05 | 1.71612 | 1.71612 |
| 18207917 | FBtr0302356 // CG1 | CG14439 | FBtr0302356 | FBtr0302356 | 1.66E-05 | 0.561001 | -1.78253 |
| 18208442 | FBtr0100644 // Vago | Vago | FBtr0100644 | FBtr0100644 | 1.69E-05 | 1.61882 | 1.61882 |
| 18150004 | FBtr0088226 // RpS1 | RpS15Ab | FBtr0088226 | FBtr0088226 | 1.71E-05 | 1.86948 | 1.86948 |
| 18186197 | FBtr0082826 // Cht5 | Cht5 | FBtr0082826 | FBtr0082826 | 1.72E-05 | 1.54372 | 1.54372 |
| 18181946 | NR_073961 // CR43 | CR43705 | NR_073961 | FBtr0321301 | 1.72E-05 | 1.74856 | 1.74856 |
| 18205969 | FBtr0073821 // Yp3 / | Yp3 | FBtr0073821 | FBtr0073821 | 1.73E-05 | 0.413287 | -2.41963 |
| 18165208 | NM_001015389 // S | Scp1 | NM_001015389 | FBtr0113713 | 1.74E-05 | 0.632235 | -1.58169 |
| 18186750 | FBtr0083405 // CG5 | CG5246 | FBtr0083405 | FBtr0083405 | 1.75E-05 | 1.55387 | 1.55387 |
| 18202247 | FBtr0112474 // CG3 | CG34279 | FBtr0112474 | FBtr0112474 | 1.80E-05 | 2.09238 | 2.09238 |
| 18188388 | FBtr0084899 // Lgr3 | Lgr3 | FBtr0084899 | FBtr0084899 | 1.81E-05 | 1.66065 | 1.66065 |
| 18199512 | FBtr0289942 // CG1 | CG10559 | FBtr0289942 | FBtr0289942 | 1.81E-05 | 1.93888 | 1.93888 |
| 18186494 | FBtr0083164 // CG5 | CG5399 | FBtr0083164 | FBtr0083164 | 1.84E-05 | 0.637081 | -1.56966 |
| 18161630 | FBtr0071782 // CG3 | CG3290 | FBtr0071782 | FBtr0071782 | 1.91E-05 | 1.80114 | 1.80114 |
| 18134371 | FBtr0079660 // CG1 | CG13385 | FBtr0079660 | FBtr0079660 | 1.91E-05 | 1.61294 | 1.61294 |
| 18175558 | FBtr0078235 // trbl / | trbl | FBtr0078235 | FBtr0078235 | 1.91E-05 | 1.54398 | 1.54398 |
| 18201360 | FBtr0084154 // CG3 | CG31174 | FBtr0084154 | FBtr0084154 | 1.92E-05 | 1.65307 | 1.65307 |
| 18153198 | NR_001754 // Uhg1 | Uhg1 | NR_001754 | FBtr0086842 | 1.93E-05 | 0.45738 | -2.18636 |
| 18138288 | FBtr0304637 // CG4 | CG43055 | FBtr0304637 | FBtr0304637 | 1.98E-05 | 0.320734 | -3.11785 |
| 18180378 | FBtr0072518 // CG3 | CG32845 | FBtr0072518 | FBtr0072518 | 2.05E-05 | 0.570088 | -1.75412 |
| 18193673 | FBtr0083585 // gl // | gl | FBtr0083585 | FBtr0083585 | 2.06E-05 | 0.600531 | -1.66519 |
| 18181922 | NM_001274928 // C | CG43680 | NM_001274928 | FBtr0310455 | 2.09E-05 | 0.51566 | -1.93926 |
| 18136302 | FBtr0080699 // yello | yellow-c | FBtr0080699 | FBtr0080699 | 2.09E-05 | 0.652341 | -1.53294 |
| 18173921 | FBtr0332065 // ImpL | ImpL3 | FBtr0332065 | FBtr0332065 | 2.11E-05 | 0.613921 | -1.62887 |
| 18151379 | FBtr0086758 // CG1 | CG18536 | FBtr0086758 | FBtr0086758 | 2.11E-05 | 1.9584 | 1.9584 |
| 18134595 | FBtr0079878 // CG4 | CG4594 | FBtr0079878 | FBtr0079878 | 2.14E-05 | 0.468111 | -2.13624 |
| 18151402 | FBtr0086663 // CG1 | CG18107 | FBtr0086663 | FBtr0086663 | 2.24E-05 | 2.75421 | 2.75421 |
| 18187568 | FBtr0084225 // CG1 | CG13855 | FBtr0084225 | FBtr0084225 | 2.24E-05 | 0.64077 | -1.56062 |
| 18188341 | FBtr0084828 // CG1 | CG13659 | FBtr0084828 | FBtr0084828 | 2.28E-05 | 1.87085 | 1.87085 |
| 18154123 | FBtr0087284 // CG3 | CG33462 | FBtr0087284 | FBtr0087284 | 2.28E-05 | 0.378119 | -2.64467 |
| 18213753 | FBtr0073468 // antd | antdh | FBtr0073468 | FBtr0073468 | 2.33E-05 | 0.437284 | -2.28684 |
| 18190607 | FBtr0083441 // CG3 | CG33333 | FBtr0083441 | FBtr0083441 | 2.33E-05 | 2.41379 | 2.41379 |
| 18164357 | NM_057464 // proP | proPO-A1 | NM_057464 | FBtr0302291 | 2.35E-05 | 1.65436 | 1.65436 |
| 18185933 | FBtr0082648 // CG6 | CG6234 | FBtr0082648 | FBtr0082648 | 2.42E-05 | 1.52087 | 1.52087 |
| 18135013 | FBtr0310149 // CG1 | CG16965 | FBtr0310149 | FBtr0310149 | 2.43E-05 | 2.17938 | 2.17938 |
| 18190561 | FBtr0100204 // CG3 | CG33095 | FBtr0100204 | FBtr0100204 | 2.45E-05 | 0.515737 | -1.93897 |
| 18156712 | FBtr0088432 // Def / | Def | FBtr0088432 | FBtr0088432 | 2.46E-05 | 3.03225 | 3.03225 |
| 18167774 | NM_001259694 // m | mRpS6 | NM_001259694 | FBtr0306093 | 2.46E-05 | 1.5099 | 1.5099 |
| 18175713 | FBtr0072601 // CG1 | CG13897 | FBtr0072601 | FBtr0072601 | 2.47E-05 | 0.470453 | -2.12561 |
| 18207332 | FBtr0070078 // CG3 | CG3176 | FBtr0070078 | FBtr0070078 | 2.48E-05 | 2.55396 | 2.55396 |
| 18157573 | NM_166118 // Gpo- | Gpo-1 | NM_166118 | FBtr0087309 | 2.51E-05 | 0.604607 | -1.65397 |
| 18167064 | FBtr0072653 // CG9 | CG9129 | FBtr0072653 | FBtr0072653 | 2.54E-05 | 1.73773 | 1.73773 |
| 18212788 | FBtr0071424 // Yp2 / | Yp2 | FBtr0071424 | FBtr0071424 | 2.57E-05 | 0.423364 | -2.36203 |
| 18189186 | FBtr0085644 // CG1 | CG15534 | FBtr0085644 | FBtr0085644 | 2.62E-05 | 1.53156 | 1.53156 |
| 18213884 | FBtr0070414 // l(1)G | l(1)G0144 | FBtr0070414 | FBtr0070414 | 2.63E-05 | 0.629141 | -1.58947 |
| 18176519 | FBtr0077129 // CG1 | CG10592 | FBtr0077129 | FBtr0077129 | 2.63E-05 | 2.46277 | 2.46277 |
| 18153130 | FBtr0072011 // yello | yellow-d | FBtr0072011 | FBtr0072011 | 2.66E-05 | 0.637579 | -1.56843 |
| 18188338 | FBtr0084827 // CG1 | CG11893 | FBtr0084827 | FBtr0084827 | 2.74E-05 | 1.50367 | 1.50367 |
| 18180560 | FBtr0100067 // CG3 | CG34012 | FBtr0100067 | FBtr0100067 | 2.75E-05 | 0.626693 | -1.59568 |
| 18165245 | FBtr0301790 // CG4 | CG41378 | FBtr0301790 | FBtr0301790 | 2.76E-05 | 0.602856 | -1.65877 |
| 18165122 | NR_073898 // CR43 | CR43793 | NR_073898 | FBtr0331988 | 2.80E-05 | 0.57181 | -1.74883 |
| 18214258 | FBtr0070658 // CG1 | CG12692 | FBtr0070658 | FBtr0070658 | 2.87E-05 | 0.588561 | -1.69906 |
| 18153699 | FBtr0071760 // CG3 | CG30281 | FBtr0071760 | FBtr0071760 | 2.96E-05 | 0.589264 | -1.69703 |
| 18133091 | FBtr0077876 // CG1 | CG14352 | FBtr0077876 | FBtr0077876 | 2.98E-05 | 1.91652 | 1.91652 |
| 18177560 | FBtr0301326 // CG6 | CG6053 | FBtr0301326 | FBtr0301326 | 3.00E-05 | 2.23081 | 2.23081 |
| 18177756 | FBtr0089322 // CG1 | CG10638 | FBtr0089322 | FBtr0089322 | 3.01E-05 | 1.80721 | 1.80721 |
| 18146552 | NR_047983 // CR43 | CR43239 | NR_047983 | FBtr0306294 | 3.09E-05 | 0.400242 | -2.49849 |
| 18217764 | FBtr0302419 // CG3 | CG33669 | FBtr0302419 | FBtr0302419 | 3.10E-05 | 1.86566 | 1.86566 |
| 18218891 | NR_047862 // CR43 | CR43493 | NR_047862 | FBtr0309524 | 3.15E-05 | 0.509128 | -1.96414 |
| 18208315 | FBtr0071389 // CG9 | CG9686 | FBtr0071389 | FBtr0071389 | 3.17E-05 | 1.7081 | 1.7081 |
| 18208317 | FBtr0071390 // CG9 | CG9689 | FBtr0071390 | FBtr0071390 | 3.21E-05 | 2.00546 | 2.00546 |
| 18215169 | FBtr0331748 // Sclp | Sclp | FBtr0331748 | FBtr0331748 | 3.24E-05 | 1.87642 | 1.87642 |
| 18176544 | FBtr0077121 // CG1 | CG10576 | FBtr0077121 | FBtr0077121 | 3.27E-05 | 0.591344 | -1.69106 |
| 18209145 | FBtr0074088 // CG8 | CG8260 | FBtr0074088 | FBtr0074088 | 3.30E-05 | 0.64323 | -1.55465 |
| 18145237 | FBtr0302519 // CG3 | CG33296 | FBtr0302519 | FBtr0302519 | 3.31E-05 | 0.586095 | -1.70621 |
| 18164936 | NR_048104 // snoR | snoRNA:2R:9445410 | NR_048104 | FBtr0309758 | 3.41E-05 | 2.06125 | 2.06125 |
| 18216707 | FBtr0077369 // CG1 | CG14615 | FBtr0077369 | FBtr0077369 | 3.43E-05 | 0.489838 | -2.04149 |
| 18141452 | FBtr0077606 // CG9 | CG9664 | FBtr0077606 | FBtr0077606 | 3.49E-05 | 0.623186 | -1.60466 |
| 18192309 | FBtr0306052 // CG4 | CG43179 | FBtr0306052 | FBtr0306052 | 3.56E-05 | 1.82834 | 1.82834 |
| 18146841 | NR_073765 // CR43 | CR43818 | NR_073765 | FBtr0332211 | 3.61E-05 | 1.8144 | 1.8144 |
| 18149513 | FBtr0088690 // Cyp6 | Cyp6a13 | FBtr0088690 | FBtr0088690 | 3.63E-05 | 2.09672 | 2.09672 |
| 18176606 | FBtr0077064 // CG1 | CG10469 | FBtr0077064 | FBtr0077064 | 3.74E-05 | 0.586138 | -1.70608 |
| 18170809 | FBtr0273402 // CG8 | CG8620 | FBtr0273402 | FBtr0273402 | 3.77E-05 | 0.431666 | -2.3166 |
| 18138552 | NR_047990 // CR43 | CR43420 | NR_047990 | FBtr0308769 | 3.86E-05 | 2.68248 | 2.68248 |
| 18154829 | NM_001144278 // M | Mlp60A | NM_001144278 | FBtr0299697 | 3.87E-05 | 1.62837 | 1.62837 |
| 18142141 | FBtr0331216 // CG1 | CG11321 | FBtr0331216 | FBtr0331216 | 3.88E-05 | 0.621533 | -1.60892 |
| 18199074 | FBtr0084481 // CG1 | CG10184 | FBtr0084481 | FBtr0084481 | 3.96E-05 | 1.71407 | 1.71407 |
| 18135262 | FBtr0080500 // CG7 | CG7110 | FBtr0080500 | FBtr0080500 | 3.96E-05 | 0.544466 | -1.83666 |
| 18139956 | NM_078788 // Acp2 | Acp29AB | NM_078788 | FBtr0079716 | 4.05E-05 | 0.650661 | -1.5369 |
| 18141305 | FBtr0307079 // CG1 | CG18641 | FBtr0307079 | FBtr0307079 | 4.08E-05 | 0.625085 | -1.59978 |
| 18144493 | FBtr0332971 // CG1 | CG18858 | FBtr0332971 | FBtr0332971 | 4.11E-05 | 0.587241 | -1.70288 |
| 18190391 | FBtr0334026 // CG3 | CG31446 | FBtr0334026 | FBtr0334026 | 4.13E-05 | 0.428336 | -2.33461 |
| 18179508 | FBtr0113188 // CG1 | CG11241 | FBtr0113188 | FBtr0113188 | 4.18E-05 | 0.618788 | -1.61606 |
| 18205167 | NR_004029 // CR40 | CR40613 | NR_004029 | FBtr0114206 | 4.19E-05 | 0.607938 | -1.6449 |
| 18136730 | FBtr0081025 // CG3 | CG31742 | FBtr0081025 | FBtr0081025 | 4.20E-05 | 0.535921 | -1.86595 |
| 18163385 | FBtr0091496 // Acp5 | Acp53C14c | FBtr0091496 | FBtr0091496 | 4.21E-05 | 0.39772 | -2.51433 |
| 18139452 | NR_001645 // snRN | snRNA:U2:34ABa | NR_001645 | FBtr0080486 | 4.24E-05 | 1.5369 | 1.5369 |
| 18132956 | FBtr0078005 // CG1 | CG13947 | FBtr0078005 | FBtr0078005 | 4.27E-05 | 2.89304 | 2.89304 |
| 18153897 | FBtr0072448 // CG3 | CG30430 | FBtr0072448 | FBtr0072448 | 4.34E-05 | 1.68186 | 1.68186 |
| 18160127 | FBtr0087646 // CG6 | CG6337 | FBtr0087646 | FBtr0087646 | 4.37E-05 | 0.500221 | -1.99912 |
| 18179723 | FBtr0075348 // PGR | PGRP-SB1 | FBtr0075348 | FBtr0075348 | 4.38E-05 | 0.625804 | -1.59795 |
| 18213037 | FBtr0074375 // CG1 | CG15865 | FBtr0074375 | FBtr0074375 | 4.40E-05 | 0.496024 | -2.01603 |
| 18182534 | FBtr0081954 // osk / | osk | FBtr0081954 | FBtr0081954 | 4.44E-05 | 2.53701 | 2.53701 |
| 18165645 | FBtr0072630 // LysE | LysE | FBtr0072630 | FBtr0072630 | 4.56E-05 | 0.442885 | -2.25792 |
| 18158210 | FBtr0112940 // CG3 | CG3530 | FBtr0112940 | FBtr0112940 | 4.56E-05 | 1.69395 | 1.69395 |
| 18143040 | FBtr0321259 // CG5 | CG5322 | FBtr0321259 | FBtr0321259 | 4.63E-05 | 0.439763 | -2.27395 |
| 18185094 | FBtr0081829 // CG1 | CG11671 | FBtr0081829 | FBtr0081829 | 4.66E-05 | 2.10577 | 2.10577 |
| 18133368 | FBtr0077642 // CG3 | CG3165 | FBtr0077642 | FBtr0077642 | 4.66E-05 | 0.531665 | -1.88088 |
| 18134874 | FBtr0080107 // CG1 | CG17107 | FBtr0080107 | FBtr0080107 | 4.68E-05 | 0.537643 | -1.85997 |
| 18187053 | FBtr0304745 // CG7 | CG7342 | FBtr0304745 | FBtr0304745 | 4.72E-05 | 0.565731 | -1.76762 |
| 18175744 | FBtr0072594 // dpr2 | dpr20 | FBtr0072594 | FBtr0072594 | 4.79E-05 | 1.57628 | 1.57628 |
| 18199740 | FBtr0085150 // CG6 | CG6277 | FBtr0085150 | FBtr0085150 | 5.02E-05 | 0.608876 | -1.64237 |
| 18216199 | FBtr0113000 // CG6 | CG6361 | FBtr0113000 | FBtr0113000 | 5.03E-05 | 0.511289 | -1.95584 |
| 18200422 | FBtr0085723 // Npc2 | Npc2g | FBtr0085723 | FBtr0085723 | 5.12E-05 | 1.84194 | 1.84194 |
| 18192132 | FBtr0304645 // CG4 | CG43061 | FBtr0304645 | FBtr0304645 | 5.52E-05 | 4.00383 | 4.00383 |
| 18152409 | FBtr0072000 // CG1 | CG13540 | FBtr0072000 | FBtr0072000 | 5.53E-05 | 0.460808 | -2.1701 |
| 18203298 | FBtr0304881 // CG4 | CG43091 | FBtr0304881 | FBtr0304881 | 5.76E-05 | 0.493617 | -2.02586 |
| 18192934 | FBtr0085640 // CecB | CecB | FBtr0085640 | FBtr0085640 | 5.84E-05 | 0.433164 | -2.30859 |
| 18153465 | FBtr0087233 // CG3 | CG30094 | FBtr0087233 | FBtr0087233 | 5.98E-05 | 1.66452 | 1.66452 |
| 18166760 | NM_168459 // Vha1 | Vha16-2 | NM_168459 | FBtr0076091 | 6.10E-05 | 3.13297 | 3.13297 |
| 18198913 | FBtr0303508 // CG1 | CG17111 | FBtr0303508 | FBtr0303508 | 6.11E-05 | 0.58145 | -1.71984 |
| 18179154 | FBtr0078223 // CG5 | CG5195 | FBtr0078223 | FBtr0078223 | 6.31E-05 | 1.97918 | 1.97918 |
| 18197421 | FBtr0331700 // CG7 | CG7362 | FBtr0331700 | FBtr0331700 | 6.41E-05 | 0.613789 | -1.62923 |
| 18135670 | FBtr0081302 // CG1 | CG16772 | FBtr0081302 | FBtr0081302 | 6.55E-05 | 0.612502 | -1.63265 |
| 18153786 | FBtr0333002 // CG3 | CG30354 | FBtr0333002 | FBtr0333002 | 6.67E-05 | 0.417276 | -2.39649 |
| 18182071 | NR_073914 // CR43 | CR43882 | NR_073914 | FBtr0332743 | 6.70E-05 | 0.283596 | -3.52614 |
| 18214221 | FBtr0333793 // CG2 | CG2875 | FBtr0333793 | FBtr0333793 | 6.87E-05 | 1.51804 | 1.51804 |
| 18214931 | FBtr0333306 // Psf3 | Psf3 | FBtr0333306 | FBtr0333306 | 6.87E-05 | 0.508932 | -1.9649 |
| 18195064 | NM_142091 // Aats- | Aats-met | NM_142091 | FBtr0082895 | 7.13E-05 | 0.590275 | -1.69412 |
| 18150416 | FBtr0113076 // wuc | wuc | FBtr0113076 | FBtr0113076 | 7.22E-05 | 1.66377 | 1.66377 |
| 18154613 | FBtr0112725 // CG3 | CG34424 | FBtr0112725 | FBtr0112725 | 7.31E-05 | 0.561065 | -1.78233 |
| 18154249 | NR_001758 // snoR | snoRNA:U29:54Eb | NR_001758 | FBtr0086846 | 7.35E-05 | 0.556997 | -1.79534 |
| 18175084 | FBtr0100134 // Clk / | Clk | FBtr0100134 | FBtr0100134 | 7.40E-05 | 0.657853 | -1.5201 |
| 18134977 | FBtr0080254 // CG4 | CG4988 | FBtr0080254 | FBtr0080254 | 7.47E-05 | 1.54403 | 1.54403 |
| 18173316 | FBtr0305909 // CG4 | CG43174 | FBtr0305909 | FBtr0305909 | 7.49E-05 | 1.99538 | 1.99538 |
| 18197383 | FBtr0082981 // CG8 | CG8066 | FBtr0082981 | FBtr0082981 | 7.61E-05 | 0.51706 | -1.93401 |
| 18218134 | FBtr0299576 // CG4 | CG42262 | FBtr0299576 | FBtr0299576 | 7.67E-05 | 1.93213 | 1.93213 |
| 18190671 | FBtr0100078 // CG3 | CG34023 | FBtr0100078 | FBtr0100078 | 7.71E-05 | 1.52368 | 1.52368 |
| 18144354 | FBtr0079713 // Perit | Peritrophin-15a | FBtr0079713 | FBtr0079713 | 7.75E-05 | 0.33676 | -2.96948 |
| 18152170 | FBtr0071827 // Mes | Mes4 | FBtr0071827 | FBtr0071827 | 7.86E-05 | 1.58461 | 1.58461 |
| 18145255 | FBtr0080564 // CG3 | CG33306 | FBtr0080564 | FBtr0080564 | 8.10E-05 | 1.70658 | 1.70658 |
| 18164956 | NR_048046 // mir-4 | mir-4975 | NR_048046 | FBtr0309586 | 8.21E-05 | 1.9231 | 1.9231 |
| 18214620 | FBtr0071131 // CG1 | CG10778 | FBtr0071131 | FBtr0071131 | 8.35E-05 | 1.63815 | 1.63815 |
| 18148601 | NM_058059 // CkIIb | CkIIbeta2 | NM_058059 | FBtr0086278 | 8.54E-05 | 1.59547 | 1.59547 |
| 18207162 | FBtr0331714 // CG1 | CG11092 | FBtr0331714 | FBtr0331714 | 8.61E-05 | 0.606244 | -1.6495 |
| 18168863 | FBtr0076163 // CG7 | CG7607 | FBtr0076163 | FBtr0076163 | 8.92E-05 | 0.645563 | -1.54904 |
| 18154257 | NR_001762 // snoR | snoRNA:U27:54Ea | NR_001762 | FBtr0086850 | 9.00E-05 | 0.581553 | -1.71953 |
| 18153348 | FBtr0087905 // CG3 | CG30047 | FBtr0087905 | FBtr0087905 | 9.01E-05 | 0.606162 | -1.64972 |
| 18140935 | FBtr0080783 // CG1 | CG15254 | FBtr0080783 | FBtr0080783 | 9.03E-05 | 2.34161 | 2.34161 |
| 18214998 | FBtr0073396 // CG1 | CG1552 | FBtr0073396 | FBtr0073396 | 9.15E-05 | 0.630531 | -1.58597 |
| 18150644 | FBtr0087547 // Arc2 | Arc2 | FBtr0087547 | FBtr0087547 | 9.18E-05 | 1.50407 | 1.50407 |
| 18209724 | NM_133130 // HP1D | HP1D3csd | NM_133130 | FBtr0074670 | 9.23E-05 | 1.63227 | 1.63227 |
| 18164729 | FBtr0306044 // CG4 | CG43195 | FBtr0306044 | FBtr0306044 | 9.32E-05 | 2.11734 | 2.11734 |
| 18198233 | FBtr0083751 // CG5 | CG5835 | FBtr0083751 | FBtr0083751 | 9.34E-05 | 0.638545 | -1.56606 |
| 18155680 | FBtr0306289 // CG4 | CG43236 | FBtr0306289 | FBtr0306289 | 9.35E-05 | 0.598496 | -1.67085 |
| 18200311 | FBtr0085571 // CG9 | CG9743 | FBtr0085571 | FBtr0085571 | 9.40E-05 | 0.664968 | -1.50383 |
| 18169994 | FBtr0075024 // CG1 | CG14075 | FBtr0075024 | FBtr0075024 | 9.55E-05 | 1.51788 | 1.51788 |
| 18138033 | FBtr0302892 // CG4 | CG42692 | FBtr0302892 | FBtr0302892 | 9.55E-05 | 1.6607 | 1.6607 |
| 18180440 | FBtr0076063 // Muc | Muc68E | FBtr0076063 | FBtr0076063 | 9.59E-05 | 1.72157 | 1.72157 |
| 18142015 | FBtr0079280 // CG9 | CG9500 | FBtr0079280 | FBtr0079280 | 9.86E-05 | 2.62008 | 2.62008 |
| 18209954 | FBtr0070007 // CG9 | CG9577 | FBtr0070007 | FBtr0070007 | 9.93E-05 | 0.611664 | -1.63489 |
| 18186987 | NM_142525 // Cyp1 | Cyp12a5 | NM_142525 | FBtr0083730 | 1.00E-04 | 0.53346 | -1.87455 |
| 18217539 | FBtr0070355 // CG3 | CG32801 | FBtr0070355 | FBtr0070355 | 0.00010003 | 1.62698 | 1.62698 |
| 18137600 | NR_003763 // snoR | snoRNA:Me28S-A3365 | NR_003763 | FBtr0114316 | 0.00010043 | 1.9498 | 1.9498 |
| 18151111 | FBtr0308764 // CG5 | CG5550 | FBtr0308764 | FBtr0308764 | 0.00010075 | 1.7123 | 1.7123 |
| 18149558 | FBtr0088708 // PGR | PGRP-SC1b | FBtr0088708 | FBtr0088708 | 0.00010287 | 2.18415 | 2.18415 |
| 18211133 | FBtr0299571 // CG4 | CG42259 | FBtr0299571 | FBtr0299571 | 0.00010545 | 0.543921 | -1.8385 |
| 18146923 | NM_079044 // Amy- | Amy-d | NM_079044 | FBtr0086983 | 0.00010613 | 0.579454 | -1.72576 |
| 18138479 | FBtr0307098 // CG4 | CG43350 | FBtr0307098 | FBtr0307098 | 0.00010732 | 0.573516 | -1.74363 |
| 18208744 | FBtr0073690 // CG3 | CG3775 | FBtr0073690 | FBtr0073690 | 0.00010813 | 1.52421 | 1.52421 |
| 18207395 | FBtr0070526 // CG1 | CG10801 | FBtr0070526 | FBtr0070526 | 0.00010902 | 1.88744 | 1.88744 |
| 18160107 | FBtr0087686 // CG6 | CG6145 | FBtr0087686 | FBtr0087686 | 0.00011118 | 0.617872 | -1.61846 |
| 18170961 | FBtr0078424 // CG3 | CG32444 | FBtr0078424 | FBtr0078424 | 0.00011273 | 0.666317 | -1.50079 |
| 18133992 | FBtr0079339 // CG1 | CG11236 | FBtr0079339 | FBtr0079339 | 0.00011378 | 0.631676 | -1.58309 |
| 18179471 | FBtr0078484 // CG7 | CG7130 | FBtr0078484 | FBtr0078484 | 0.000115 | 1.66158 | 1.66158 |
| 18138004 | FBtr0302734 // wb / | wb | FBtr0302734 | FBtr0302734 | 0.00011501 | 0.644205 | -1.5523 |
| 18217618 | FBtr0071239 // CG3 | CG33223 | FBtr0071239 | FBtr0071239 | 0.00011534 | 0.413825 | -2.41648 |
| 18197296 | NM_001260160 // P | Pk1r | NM_001260160 | FBtr0305681 | 0.00011563 | 0.609865 | -1.63971 |
| 18178058 | FBtr0075665 // CG1 | CG12310 | FBtr0075665 | FBtr0075665 | 0.00011605 | 1.58724 | 1.58724 |
| 18142019 | FBtr0329952 // CG9 | CG9527 | FBtr0329952 | FBtr0329952 | 0.00011733 | 0.632801 | -1.58027 |
| 18210889 | FBtr0112557 // CG3 | CG34348 | FBtr0112557 | FBtr0112557 | 0.00011841 | 0.627418 | -1.59383 |
| 18200657 | FBtr0113321 // Ugt8 | Ugt86Dd | FBtr0113321 | FBtr0113321 | 0.00011912 | 0.619052 | -1.61537 |
| 18217144 | NR_002468 // pncr0 | pncr004:X | NR_002468 | FBtr0091949 | 0.00012206 | 0.509853 | -1.96135 |
| 18160624 | FBtr0087121 // CG8 | CG8311 | FBtr0087121 | FBtr0087121 | 0.00012454 | 0.572303 | -1.74733 |
| 18196690 | FBtr0100320 // fabp | fabp | FBtr0100320 | FBtr0100320 | 0.00012527 | 1.64642 | 1.64642 |
| 18178676 | FBtr0075117 // CG4 | CG4306 | FBtr0075117 | FBtr0075117 | 0.0001269 | 0.646138 | -1.54766 |
| 18157348 | NM_057861 // T3dh | T3dh | NM_057861 | FBtr0071848 | 0.00012789 | 0.617302 | -1.61995 |
| 18206495 | FBtr0077211 // mst / | mst | FBtr0077211 | FBtr0077211 | 0.00013075 | 0.579681 | -1.72509 |
| 18143895 | FBtr0081222 // fon / | fon | FBtr0081222 | FBtr0081222 | 0.00013176 | 0.476646 | -2.09799 |
| 18214885 | FBtr0112968 // CG1 | CG1791 | FBtr0112968 | FBtr0112968 | 0.00013214 | 0.521959 | -1.91586 |
| 18153185 | NM_166395 // Obp5 | Obp57a | NM_166395 | FBtr0086294 | 0.00013268 | 0.631615 | -1.58324 |
| 18200221 | FBtr0085514 // capa | capa | FBtr0085514 | FBtr0085514 | 0.00013344 | 1.91766 | 1.91766 |
| 18144692 | FBtr0077893 // CG3 | CG31663 | FBtr0077893 | FBtr0077893 | 0.00013362 | 0.608605 | -1.6431 |
| 18165929 | FBtr0076613 // mtrm | mtrm | FBtr0076613 | FBtr0076613 | 0.0001341 | 0.468432 | -2.13478 |
| 18215546 | FBtr0073977 // CG1 | CG14406 | FBtr0073977 | FBtr0073977 | 0.00013617 | 0.478981 | -2.08776 |
| 18180334 | FBtr0078461 // CG3 | CG32447 | FBtr0078461 | FBtr0078461 | 0.00013693 | 1.81481 | 1.81481 |
| 18137500 | FBtr0300048 // CG3 | CG34180 | FBtr0300048 | FBtr0300048 | 0.00013852 | 0.430238 | -2.32429 |
| 18183067 | FBtr0082572 // GstD | GstD5 | FBtr0082572 | FBtr0082572 | 0.00014072 | 2.55082 | 2.55082 |
| 18138528 | FBtr0308744 // CG4 | CG43401 | FBtr0308744 | FBtr0308744 | 0.00014449 | 0.325095 | -3.07603 |
| 18151163 | FBtr0300277 // CG6 | CG6967 | FBtr0300277 | FBtr0300277 | 0.00014547 | 2.10732 | 2.10732 |
| 18154945 | FBtr0299961 // CG4 | CG42362 | FBtr0299961 | FBtr0299961 | 0.0001464 | 0.418591 | -2.38897 |
| 18154949 | FBtr0299961 // CG4 | CG42362 | FBtr0299961 | FBtr0299961 | 0.0001464 | 0.418591 | -2.38897 |
| 18147398 | NM_079118 // betaT | betaTub60D | NM_079118 | FBtr0072270 | 0.00014829 | 0.579495 | -1.72564 |
| 18168741 | NM_079288 // Ilp2 / | Ilp2 | NM_079288 | FBtr0076329 | 0.0001506 | 1.62548 | 1.62548 |
| 18151443 | FBtr0113093 // CG1 | CG10924 | FBtr0113093 | FBtr0113093 | 0.00015125 | 2.50256 | 2.50256 |
| 18177141 | FBtr0076552 // CG5 | CG5280 | FBtr0076552 | FBtr0076552 | 0.00015361 | 1.68983 | 1.68983 |
| 18151208 | FBtr0086919 // CG1 | CG10764 | FBtr0086919 | FBtr0086919 | 0.00015433 | 0.48882 | -2.04574 |
| 18147139 | NM_206179 // mei- | mei-W68 | NM_206179 | FBtr0086461 | 0.00015949 | 1.51306 | 1.51306 |
| 18152096 | FBtr0071726 // CG9 | CG9304 | FBtr0071726 | FBtr0071726 | 0.00016205 | 0.643362 | -1.55434 |
| 18168155 | FBtr0076833 // CG1 | CG14826 | FBtr0076833 | FBtr0076833 | 0.00016405 | 0.469954 | -2.12787 |
| 18168152 | FBtr0076913 // CG1 | CG15829 | FBtr0076913 | FBtr0076913 | 0.00016454 | 2.32674 | 2.32674 |
| 18181200 | FBtr0301939 // Sfp7 | Sfp78E | FBtr0301939 | FBtr0301939 | 0.00016557 | 0.651365 | -1.53524 |
| 18146614 | NR_047994 // CR43 | CR43358 | NR_047994 | FBtr0307108 | 0.00016618 | 2.86784 | 2.86784 |
| 18215539 | FBtr0073979 // CG9 | CG9512 | FBtr0073979 | FBtr0073979 | 0.0001709 | 0.617466 | -1.61952 |
| 18197103 | FBtr0082726 // CG7 | CG7381 | FBtr0082726 | FBtr0082726 | 0.00017113 | 0.488687 | -2.0463 |
| 18205120 | FBtr0302216 // CG4 | CG40249 | FBtr0302216 | FBtr0302216 | 0.00017596 | 1.82403 | 1.82403 |
| 18172297 | FBtr0112435 // CG3 | CG34241 | FBtr0112435 | FBtr0112435 | 0.00017751 | 1.56775 | 1.56775 |
| 18139440 | FBtr0081347 // vls // | vls | FBtr0081347 | FBtr0081347 | 0.00017793 | 1.7124 | 1.7124 |
| 18143605 | FBtr0331624 // CG1 | CG13282 | FBtr0331624 | FBtr0331624 | 0.00017977 | 0.659118 | -1.51718 |
| 18186765 | FBtr0083438 // CG5 | CG5860 | FBtr0083438 | FBtr0083438 | 0.00018459 | 0.372301 | -2.686 |
| 18154241 | NR_001765 // snoR | snoRNA:U31:54Eb | NR_001765 | FBtr0086853 | 0.000188 | 0.536091 | -1.86535 |
| 18168995 | FBtr0076042 // CG1 | CG17826 | FBtr0076042 | FBtr0076042 | 0.0001938 | 1.94682 | 1.94682 |
| 18169114 | FBtr0075868 // CG1 | CG14120 | FBtr0075868 | FBtr0075868 | 0.00019639 | 1.90209 | 1.90209 |
| 18176125 | FBtr0073111 // CG1 | CG14963 | FBtr0073111 | FBtr0073111 | 0.00019692 | 0.611125 | -1.63633 |
| 18167624 | FBtr0300062 // CG1 | CG10357 | FBtr0300062 | FBtr0300062 | 0.00020051 | 1.94505 | 1.94505 |
| 18154134 | FBtr0088260 // CG3 | CG33474 | FBtr0088260 | FBtr0088260 | 0.0002008 | 1.59618 | 1.59618 |
| 18136891 | FBtr0077757 // CG3 | CG31949 | FBtr0077757 | FBtr0077757 | 0.00020453 | 1.65574 | 1.65574 |
| 18143308 | FBtr0080360 // rho-6 | rho-6 | FBtr0080360 | FBtr0080360 | 0.00020828 | 1.93581 | 1.93581 |
| 18186801 | FBtr0083463 // CG1 | CG14329 | FBtr0083463 | FBtr0083463 | 0.0002103 | 2.30479 | 2.30479 |
| 18146241 | FBtr0301934 // Sfp2 | Sfp24Bc | FBtr0301934 | FBtr0301934 | 0.00021833 | 2.10024 | 2.10024 |
| 18132577 | FBtr0080801 // beat- | beat-Ic | FBtr0080801 | FBtr0080801 | 0.00022124 | 1.53671 | 1.53671 |
| 18144739 | FBtr0081365 // CG3 | CG31683 | FBtr0081365 | FBtr0081365 | 0.00022396 | 0.456596 | -2.19012 |
| 18177020 | NM_139964 // Oseg | Oseg1 | NM_139964 | FBtr0076663 | 0.00022927 | 1.97718 | 1.97718 |
| 18142139 | FBtr0290242 // CG1 | CG11322 | FBtr0290242 | FBtr0290242 | 0.00022961 | 1.64345 | 1.64345 |
| 18186565 | FBtr0083203 // blp / | blp | FBtr0083203 | FBtr0083203 | 0.00023013 | 1.56211 | 1.56211 |
| 18175510 | FBtr0075722 // Lk // | Lk | FBtr0075722 | FBtr0075722 | 0.00023116 | 1.61798 | 1.61798 |
| 18174575 | FBtr0075290 // a10 / | a10 | FBtr0075290 | FBtr0075290 | 0.0002377 | 1.59649 | 1.59649 |
| 18201178 | FBtr0290052 // CG3 | CG31097 | FBtr0290052 | FBtr0290052 | 0.00023832 | 1.66879 | 1.66879 |
| 18157088 | FBtr0087455 // Cyp6 | Cyp6a8 | FBtr0087455 | FBtr0087455 | 0.00024069 | 0.613489 | -1.63002 |
| 18153427 | FBtr0087555 // CG3 | CG30075 | FBtr0087555 | FBtr0087555 | 0.00024315 | 1.9162 | 1.9162 |
| 18153766 | FBtr0305317 // CG3 | CG30345 | FBtr0305317 | FBtr0305317 | 0.00024467 | 1.50153 | 1.50153 |
| 18154247 | NR_001759 // snoR | snoRNA:U29:54Ec | NR_001759 | FBtr0086847 | 0.00024991 | 0.561005 | -1.78252 |
| 18150672 | FBtr0087488 // CG1 | CG17386 | FBtr0087488 | FBtr0087488 | 0.00025025 | 0.468814 | -2.13304 |
| 18146854 | NR_073807 // CR43 | CR43839 | NR_073807 | FBtr0332319 | 0.0002529 | 2.25809 | 2.25809 |
| 18177175 | FBtr0301699 // UGP | UGP | FBtr0301699 | FBtr0301699 | 0.00025832 | 0.65494 | -1.52686 |
| 18154231 | NR_001763 // snoR | snoRNA:Me28S-G3277a | NR_001763 | FBtr0086851 | 0.00026521 | 0.527947 | -1.89413 |
| 18153228 | NM_136665 // GstT | GstT1 | NM_136665 | FBtr0088463 | 0.00026959 | 1.62188 | 1.62188 |
| 18196230 | FBtr0082112 // CG1 | CG16790 | FBtr0082112 | FBtr0082112 | 0.0002702 | 0.560487 | -1.78416 |
| 18208796 | FBtr0073768 // dmrt | dmrt11E | FBtr0073768 | FBtr0073768 | 0.00027187 | 0.373126 | -2.68006 |
| 18187824 | FBtr0303150 // CG1 | CG16710 | FBtr0303150 | FBtr0303150 | 0.00027326 | 2.0886 | 2.0886 |
| 18206147 | NM_057512 // Pgd / | Pgd | NM_057512 | FBtr0070384 | 0.0002779 | 0.659219 | -1.51695 |
| 18173220 | FBtr0304959 // CG4 | CG43120 | FBtr0304959 | FBtr0304959 | 0.00028055 | 1.54192 | 1.54192 |
| 18173062 | FBtr0303852 // CG4 | CG42833 | FBtr0303852 | FBtr0303852 | 0.00028362 | 0.640452 | -1.5614 |
| 18210340 | FBtr0077183 // CG3 | CG32523 | FBtr0077183 | FBtr0077183 | 0.00028826 | 0.65057 | -1.53711 |
| 18216050 | FBtr0074444 // CG8 | CG8661 | FBtr0074444 | FBtr0074444 | 0.00028857 | 0.534398 | -1.87127 |
| 18216319 | FBtr0074703 // CG1 | CG15882 | FBtr0074703 | FBtr0074703 | 0.0002892 | 1.61585 | 1.61585 |
| 18209853 | FBtr0074745 // Sec6 | Sec61gamma | FBtr0074745 | FBtr0074745 | 0.0002894 | 0.424997 | -2.35296 |
| 18141312 | FBtr0077781 // CG3 | CG3528 | FBtr0077781 | FBtr0077781 | 0.00029147 | 0.561139 | -1.78209 |
| 18195834 | FBtr0081591 // CG1 | CG10029 | FBtr0081591 | FBtr0081591 | 0.00029345 | 0.662533 | -1.50936 |
| 18176436 | FBtr0308822 // CG1 | CG13707 | FBtr0308822 | FBtr0308822 | 0.00029586 | 1.51589 | 1.51589 |
| 18201512 | FBtr0083198 // CG3 | CG31294 | FBtr0083198 | FBtr0083198 | 0.00030212 | 1.50802 | 1.50802 |
| 18202255 | FBtr0112479 // CG3 | CG34283 | FBtr0112479 | FBtr0112479 | 0.00030363 | 0.566427 | -1.76545 |
| 18176058 | FBtr0073011 // spz5 | spz5 | FBtr0073011 | FBtr0073011 | 0.00030712 | 0.54377 | -1.83901 |
| 18218022 | NR_002463 // snoR | snoRNA:Psi28S-3436b | NR_002463 | FBtr0091803 | 0.00031609 | 1.50234 | 1.50234 |
| 18216541 | FBtr0070027 // Phf7 | Phf7 | FBtr0070027 | FBtr0070027 | 0.00031924 | 1.52479 | 1.52479 |
| 18218456 | FBtr0070106 // CG1 | CG13377 | FBtr0070106 | FBtr0070106 | 0.00032189 | 0.646445 | -1.54692 |
| 18155617 | FBtr0305124 // CG4 | CG43123 | FBtr0305124 | FBtr0305124 | 0.0003239 | 0.478006 | -2.09203 |
| 18161362 | FBtr0071561 // CG1 | CG17999 | FBtr0071561 | FBtr0071561 | 0.00032828 | 0.666189 | -1.50108 |
| 18191847 | FBtr0302394 // CG4 | CG42650 | FBtr0302394 | FBtr0302394 | 0.00033933 | 1.5277 | 1.5277 |
| 18212088 | FBtr0070504 // CG2 | CG2650 | FBtr0070504 | FBtr0070504 | 0.00034377 | 1.8166 | 1.8166 |
| 18138736 | NR_073750 // CR43 | CR43766 | NR_073750 | FBtr0331283 | 0.00034381 | 1.80749 | 1.80749 |
| 18145433 | FBtr0302372 // CG3 | CG34049 | FBtr0302372 | FBtr0302372 | 0.00034503 | 0.56766 | -1.76162 |
| 18162671 | FBtr0086963 // CG3 | CG30101 | FBtr0086963 | FBtr0086963 | 0.00035105 | 1.89447 | 1.89447 |
| 18208684 | FBtr0073655 // CG2 | CG2543 | FBtr0073655 | FBtr0073655 | 0.00035985 | 0.523431 | -1.91047 |
| 18176566 | FBtr0077113 // mad | mad2 | FBtr0077113 | FBtr0077113 | 0.00036129 | 1.70824 | 1.70824 |
| 18212309 | FBtr0073841 // l(1)d | l(1)dd4 | FBtr0073841 | FBtr0073841 | 0.00036246 | 0.608015 | -1.6447 |
| 18161610 | FBtr0071787 // CG3 | CG3045 | FBtr0071787 | FBtr0071787 | 0.00036449 | 1.73588 | 1.73588 |
| 18163056 | FBtr0302316 // CG3 | CG30414 | FBtr0302316 | FBtr0302316 | 0.00036752 | 1.6057 | 1.6057 |
| 18206433 | NM_080305 // Cyp4 | Cyp4ae1 | NM_080305 | FBtr0070388 | 0.00037597 | 0.64662 | -1.5465 |
| 18192788 | NR_074016 // CR43 | CR43849 | NR_074016 | FBtr0332551 | 0.00038004 | 1.51117 | 1.51117 |
| 18131338 | FBtr0080281 // Cry / | Cry | FBtr0080281 | FBtr0080281 | 0.00038123 | 1.62029 | 1.62029 |
| 18179836 | FBtr0076241 // CG3 | CG32069 | FBtr0076241 | FBtr0076241 | 0.00038281 | 1.69443 | 1.69443 |
| 18203193 | NR_048407 // CR42 | CR42839 | NR_048407 | FBtr0309032 | 0.00038478 | 0.561647 | -1.78048 |
| 18157185 | NM_079124 // Ssl // | Ssl | NM_079124 | FBtr0072351 | 0.00038764 | 1.62813 | 1.62813 |
| 18200519 | FBtr0085809 // CG1 | CG11334 | FBtr0085809 | FBtr0085809 | 0.00038861 | 0.655871 | -1.52469 |
| 18168381 | FBtr0076608 // Jon6 | Jon66Ci | FBtr0076608 | FBtr0076608 | 0.00040446 | 1.9664 | 1.9664 |
| 18168592 | FBtr0076444 // CG4 | CG4447 | FBtr0076444 | FBtr0076444 | 0.00041266 | 1.58549 | 1.58549 |
| 18158274 | FBtr0086955 // mthl | mthl3 | FBtr0086955 | FBtr0086955 | 0.00041398 | 0.477508 | -2.09421 |
| 18178112 | FBtr0332815 // CG7 | CG7804 | FBtr0332815 | FBtr0332815 | 0.00043575 | 1.63561 | 1.63561 |
| 18162802 | FBtr0071927 // CG3 | CG30196 | FBtr0071927 | FBtr0071927 | 0.00044001 | 2.16981 | 2.16981 |
| 18183061 | NM_080173 // GstD | GstD2 | NM_080173 | FBtr0082569 | 0.00044431 | 1.95725 | 1.95725 |
| 18165758 | NR_003122 // Eip63 | Eip63F-2 | NR_003122 | FBtr0073162 | 0.00046001 | 1.58876 | 1.58876 |
| 18144137 | FBtr0290042 // Oseg | Oseg5 | FBtr0290042 | FBtr0290042 | 0.00046109 | 1.8563 | 1.8563 |
| 18151879 | FBtr0071514 // CG1 | CG15227 | FBtr0071514 | FBtr0071514 | 0.00047715 | 0.615591 | -1.62446 |
| 18184963 | FBtr0303218 // CG1 | CG14607 | FBtr0303218 | FBtr0303218 | 0.00048296 | 1.58563 | 1.58563 |
| 18136798 | FBtr0290062 // CG3 | CG31812 | FBtr0290062 | FBtr0290062 | 0.00050265 | 0.602139 | -1.66075 |
| 18193278 | FBtr0084472 // nau / | nau | FBtr0084472 | FBtr0084472 | 0.00051414 | 0.625839 | -1.59786 |
| 18166270 | NM_079232 // mei-P | mei-P22 | NM_079232 | FBtr0076836 | 0.00051586 | 1.73823 | 1.73823 |
| 18183073 | NM_080177 // GstD | GstD8 | NM_080177 | FBtr0082575 | 0.00051724 | 1.9155 | 1.9155 |
| 18199724 | FBtr0085078 // CG6 | CG6296 | FBtr0085078 | FBtr0085078 | 0.00051909 | 0.547182 | -1.82755 |
| 18162878 | FBtr0071549 // CG3 | CG30291 | FBtr0071549 | FBtr0071549 | 0.00053629 | 0.628347 | -1.59148 |
| 18152278 | FBtr0071880 // CG1 | CG13527 | FBtr0071880 | FBtr0071880 | 0.00053659 | 0.622289 | -1.60697 |
| 18208961 | FBtr0073908 // CG1 | CG1461 | FBtr0073908 | FBtr0073908 | 0.00055516 | 0.662247 | -1.51001 |
| 18193002 | FBtr0084106 // e // | e | FBtr0084106 | FBtr0084106 | 0.00055995 | 0.585116 | -1.70906 |
| 18161298 | NM_166397 // Obp5 | Obp57d | NM_166397 | FBtr0299804 | 0.00056741 | 0.49603 | -2.01601 |
| 18162481 | NM_166397 // Obp5 | Obp57d | NM_166397 | FBtr0299804 | 0.00056741 | 0.49603 | -2.01601 |
| 18161905 | FBtr0072107 // CG1 | CG17664 | FBtr0072107 | FBtr0072107 | 0.00057183 | 0.546254 | -1.83065 |
| 18176228 | FBtr0073172 // CG1 | CG12766 | FBtr0073172 | FBtr0073172 | 0.00057826 | 0.629597 | -1.58832 |
| 18177529 | FBtr0076140 // Muc | Muc68Ca | FBtr0076140 | FBtr0076140 | 0.0005793 | 1.68385 | 1.68385 |
| 18180021 | FBtr0074914 // CG3 | CG32219 | FBtr0074914 | FBtr0074914 | 0.00059244 | 1.52338 | 1.52338 |
| 18175765 | FBtr0072734 // CG9 | CG9192 | FBtr0072734 | FBtr0072734 | 0.00059889 | 1.87466 | 1.87466 |
| 18211858 | FBtr0306816 // CG4 | CG43288 | FBtr0306816 | FBtr0306816 | 0.00062866 | 0.472823 | -2.11495 |
| 18138661 | NR_073723 // CR43 | CR43713 | NR_073723 | FBtr0329835 | 0.00063336 | 1.61701 | 1.61701 |
| 18160818 | FBtr0086892 // P32 / | P32 | FBtr0086892 | FBtr0086892 | 0.00063534 | 0.637185 | -1.5694 |
| 18181193 | FBtr0301744 // CG4 | CG42590 | FBtr0301744 | FBtr0301744 | 0.00063814 | 0.509756 | -1.96172 |
| 18168494 | FBtr0076512 // CG1 | CG13313 | FBtr0076512 | FBtr0076512 | 0.00064285 | 1.84644 | 1.84644 |
| 18177066 | FBtr0273348 // CG1 | CG13305 | FBtr0273348 | FBtr0273348 | 0.00064698 | 1.5926 | 1.5926 |
| 18148567 | NM_166277 // IM2 / | IM2 | NM_166277 | FBtr0086664 | 0.00066347 | 1.80204 | 1.80204 |
| 18179592 | FBtr0075674 // CG1 | CG13465 | FBtr0075674 | FBtr0075674 | 0.0006685 | 2.24041 | 2.24041 |
| 18216640 | FBtr0077266 // CG1 | CG15449 | FBtr0077266 | FBtr0077266 | 0.00067806 | 0.657345 | -1.52127 |
| 18190932 | FBtr0112495 // CG3 | CG34299 | FBtr0112495 | FBtr0112495 | 0.00067947 | 0.589389 | -1.69667 |
| 18216402 | FBtr0074783 // CG1 | CG12204 | FBtr0074783 | FBtr0074783 | 0.00068345 | 0.610913 | -1.63689 |
| 18212581 | NR_002129 // snRN | snRNA:U5:14B | NR_002129 | FBtr0074249 | 0.0006999 | 1.88188 | 1.88188 |
| 18133759 | NM_135074 // Cyp4 | Cyp4ac3 | NM_135074 | FBtr0079068 | 0.00071619 | 0.650991 | -1.53612 |
| 18169765 | FBtr0331845 // CG7 | CG7692 | FBtr0331845 | FBtr0331845 | 0.0007181 | 1.59679 | 1.59679 |
| 18197229 | FBtr0082861 // CG9 | CG9759 | FBtr0082861 | FBtr0082861 | 0.00072959 | 0.566193 | -1.76618 |
| 18172199 | FBtr0112768 // CG3 | CG34462 | FBtr0112768 | FBtr0112768 | 0.00073478 | 0.473695 | -2.11107 |
| 18200701 | FBtr0112769 // CG1 | CG11741 | FBtr0112769 | FBtr0112769 | 0.00075009 | 0.628934 | -1.58999 |
| 18161564 | FBtr0071733 // CG1 | CG13494 | FBtr0071733 | FBtr0071733 | 0.00075074 | 1.83154 | 1.83154 |
| 18136286 | NM_078819 // Gr32 | Gr32a | NM_078819 | FBtr0080198 | 0.00075426 | 0.586832 | -1.70407 |
| 18145065 | NM_164466 // Tengl | Tengl2 | NM_164466 | FBtr0077808 | 0.00076348 | 1.74704 | 1.74704 |
| 18177695 | FBtr0075989 // GRH | GRHRII | FBtr0075989 | FBtr0075989 | 0.00079562 | 0.628662 | -1.59068 |
| 18140091 | NM_165385 // nomp | nompB | NM_165385 | --- | 0.00079852 | 0.654676 | -1.52747 |
| 18203403 | FBtr0306166 // CG4 | CG43222 | FBtr0306166 | FBtr0306166 | 0.00081287 | 1.5335 | 1.5335 |
| 18134439 | FBtr0079767 // CG1 | CG12439 | FBtr0079767 | FBtr0079767 | 0.00081809 | 2.81607 | 2.81607 |
| 18137378 | NR_003766 // snoR | snoRNA:Psi18S-525b | NR_003766 | FBtr0113541 | 0.00082147 | 0.510896 | -1.95735 |
| 18215987 | FBtr0332534 // CG9 | CG9059 | FBtr0332534 | FBtr0332534 | 0.00082299 | 1.52955 | 1.52955 |
| 18200707 | NR_048380 // CR17 | CR17025 | NR_048380 | FBtr0309273 | 0.00083299 | 0.50236 | -1.99061 |
| 18184755 | NM_079521 // NPFR | NPFR1 | NM_079521 | FBtr0078590 | 0.00087932 | 0.584844 | -1.70986 |
| 18192162 | NR_048362 // mir-3 | mir-34 | NR_048362 | FBtr0304499 | 0.00088725 | 2.24721 | 2.24721 |
| 18156744 | FBtr0088184 // TpnC | TpnC47D | FBtr0088184 | FBtr0088184 | 0.00090909 | 0.540687 | -1.8495 |
| 18154096 | FBtr0086169 // ppk2 | ppk25 | FBtr0086169 | FBtr0086169 | 0.00091044 | 1.79442 | 1.79442 |
| 18146541 | FBtr0305706 // CG4 | CG43165 | FBtr0305706 | FBtr0305706 | 0.00091685 | 1.7089 | 1.7089 |
| 18189725 | FBtr0083972 // TotC | TotC | FBtr0083972 | FBtr0083972 | 0.00092708 | 1.76993 | 1.76993 |
| 18150112 | FBtr0304901 // CG1 | CG13203 | FBtr0304901 | FBtr0304901 | 0.00095452 | 0.627952 | -1.59248 |
| 18133160 | FBtr0077827 // CG1 | CG12674 | FBtr0077827 | FBtr0077827 | 0.00095985 | 1.52531 | 1.52531 |
| 18215026 | FBtr0073473 // CG1 | CG15199 | FBtr0073473 | FBtr0073473 | 0.00104156 | 1.79691 | 1.79691 |
| 18172510 | FBtr0300318 // Sfp7 | Sfp79B | FBtr0300318 | FBtr0300318 | 0.00104199 | 0.518036 | -1.93037 |
| 18130846 | FBtr0079808 // Fbp2 | Fbp2 | FBtr0079808 | FBtr0079808 | 0.00106853 | 0.5219 | -1.91608 |
| 18154255 | NR_001768 // snoR | snoRNA:U27:54Eb | NR_001768 | FBtr0086856 | 0.00107977 | 0.587336 | -1.7026 |
| 18213491 | FBtr0070501 // Csat | Csat | FBtr0070501 | FBtr0070501 | 0.00108291 | 0.499497 | -2.00201 |
| 18197619 | FBtr0083154 // CG1 | CG18522 | FBtr0083154 | FBtr0083154 | 0.0011338 | 0.637831 | -1.56781 |
| 18137701 | NM_136017 // rdo // | rdo | NM_136017 | FBtr0081029 | 0.00116037 | 1.53593 | 1.53593 |
| 18163737 | FBtr0301011 // CG3 | CG34236 | FBtr0301011 | FBtr0301011 | 0.00116147 | 0.617148 | -1.62036 |
| 18138319 | NR_047995 // let-7- | let-7-C | NR_047995 | FBtr0306973 | 0.00116871 | 0.570654 | -1.75238 |
| 18161727 | FBtr0071928 // Cyp6 | Cyp6d2 | FBtr0071928 | FBtr0071928 | 0.00119611 | 1.84927 | 1.84927 |
| 18131802 | NM_057866 // Rca1 | Rca1 | NM_057866 | FBtr0079342 | 0.00124903 | 0.650585 | -1.53708 |
| 18163907 | FBtr0333065 // jeb / | jeb | FBtr0333065 | FBtr0333065 | 0.0012805 | 1.61892 | 1.61892 |
| 18151643 | FBtr0310321 // CG1 | CG10073 | FBtr0310321 | FBtr0310321 | 0.00129913 | 0.463138 | -2.15919 |
| 18138786 | NM_001273019 // C | CG43851 | NM_001273019 | FBtr0332512 | 0.00132794 | 1.53412 | 1.53412 |
| 18141236 | FBtr0077854 // CG4 | CG4259 | FBtr0077854 | FBtr0077854 | 0.00132794 | 1.71925 | 1.71925 |
| 18169796 | FBtr0075223 // qjt // | qjt | FBtr0075223 | FBtr0075223 | 0.00133033 | 0.660209 | -1.51467 |
| 18201204 | FBtr0273357 // CG3 | CG31105 | FBtr0273357 | FBtr0273357 | 0.00134088 | 0.596133 | -1.67748 |
| 18201018 | NM_170497 // PH4a | PH4alphaSG1 | NM_170497 | FBtr0085686 | 0.00136658 | 0.59184 | -1.68965 |
| 18189261 | FBtr0085710 // Cpr1 | Cpr100A | FBtr0085710 | FBtr0085710 | 0.00140875 | 1.5333 | 1.5333 |
| 18182065 | NR_073972 // CR43 | CR43878 | NR_073972 | FBtr0332737 | 0.00142058 | 0.656438 | -1.52337 |
| 18176455 | FBtr0077180 // CG1 | CG10673 | FBtr0077180 | FBtr0077180 | 0.00144854 | 0.615921 | -1.62358 |
| 18203732 | NR_074060 // CR43 | CR43847 | NR_074060 | FBtr0332549 | 0.00149003 | 1.55215 | 1.55215 |
| 18154233 | NR_001760 // snoR | snoRNA:U76:54Eb | NR_001760 | FBtr0086848 | 0.00149093 | 0.509278 | -1.96356 |
| 18154253 | NR_001770 // snoR | snoRNA:Me18S-A28a | NR_001770 | FBtr0086858 | 0.00151336 | 0.499767 | -2.00093 |
| 18184956 | FBtr0303219 // CG1 | CG14608 | FBtr0303219 | FBtr0303219 | 0.00151849 | 1.85767 | 1.85767 |
| 18186310 | FBtr0082957 // CG1 | CG14852 | FBtr0082957 | FBtr0082957 | 0.0015473 | 1.93968 | 1.93968 |
| 18131155 | NR_001600 // snRN | snRNA:U3:22A | NR_001600 | FBtr0077928 | 0.0015651 | 2.32855 | 2.32855 |
| 18151681 | FBtr0086463 // CG1 | CG15120 | FBtr0086463 | FBtr0086463 | 0.00163443 | 1.92511 | 1.92511 |
| 18141223 | FBtr0077892 // CG1 | CG15358 | FBtr0077892 | FBtr0077892 | 0.00164432 | 0.612235 | -1.63336 |
| 18148206 | NM_166184 // Cdk4 | Cdk4 | NM_166184 | FBtr0087103 | 0.00164462 | 0.646837 | -1.54598 |
| 18155832 | NR_048125 // CR43 | CR43416 | NR_048125 | FBtr0308763 | 0.0016592 | 1.58878 | 1.58878 |
| 18207391 | FBtr0332493 // CG3 | CG3603 | FBtr0332493 | FBtr0332493 | 0.00167127 | 0.601042 | -1.66378 |
| 18154360 | NR_003823 // snoR | snoRNA:Psi18S-1389b | NR_003823 | FBtr0113526 | 0.00168896 | 1.62062 | 1.62062 |
| 18139519 | FBtr0080166 // piwi | piwi | FBtr0080166 | FBtr0080166 | 0.00172263 | 0.661219 | -1.51236 |
| 18174590 | FBtr0076299 // can / | can | FBtr0076299 | FBtr0076299 | 0.00174633 | 0.607577 | -1.64588 |
| 18175099 | FBtr0075207 // Jon7 | Jon74E | FBtr0075207 | FBtr0075207 | 0.00175573 | 1.75713 | 1.75713 |
| 18138734 | NR_073785 // CR43 | CR43763 | NR_073785 | FBtr0331275 | 0.00181435 | 0.634286 | -1.57657 |
| 18179849 | FBtr0076222 // CG3 | CG32074 | FBtr0076222 | FBtr0076222 | 0.00186523 | 2.04778 | 2.04778 |
| 18178827 | FBtr0075033 // CG1 | CG10424 | FBtr0075033 | FBtr0075033 | 0.00189227 | 0.618097 | -1.61787 |
| 18213687 | FBtr0307095 // CG1 | CG17636 | FBtr0307095 | FBtr0307095 | 0.00191361 | 0.589732 | -1.69569 |
| 18149890 | FBtr0310142 // CG1 | CG12910 | FBtr0310142 | FBtr0310142 | 0.00193857 | 0.653424 | -1.5304 |
| 18217486 | FBtr0070881 // CG3 | CG32750 | FBtr0070881 | FBtr0070881 | 0.00194846 | 1.61348 | 1.61348 |
| 18146698 | NR_047928 // CR43 | CR43621 | NR_047928 | FBtr0309976 | 0.00195414 | 2.18315 | 2.18315 |
| 18177840 | FBtr0075936 // CG1 | CG10943 | FBtr0075936 | FBtr0075936 | 0.00196075 | 0.574676 | -1.74011 |
| 18208103 | FBtr0071158 // CG1 | CG18262 | FBtr0071158 | FBtr0071158 | 0.00198412 | 0.621792 | -1.60826 |
| 18187583 | NM_142794 // Pebp | Pebp1 | NM_142794 | FBtr0084255 | 0.00200193 | 0.626036 | -1.59735 |
| 18169870 | NM_079410 // mRpS | mRpS26 | NM_079410 | FBtr0075121 | 0.00203021 | 0.644817 | -1.55083 |
| 18172649 | FBtr0301252 // CG4 | CG42553 | FBtr0301252 | FBtr0301252 | 0.00206758 | 1.51795 | 1.51795 |
| 18193265 | FBtr0082662 // mus | mus308 | FBtr0082662 | FBtr0082662 | 0.00209787 | 1.75722 | 1.75722 |
| 18146640 | NR_048017 // CR43 | CR43414 | NR_048017 | FBtr0308759 | 0.00215807 | 1.58551 | 1.58551 |
| 18195856 | FBtr0081773 // CG3 | CG3014 | FBtr0081773 | FBtr0081773 | 0.00216834 | 0.64699 | -1.54562 |
| 18158542 | FBtr0089062 // Tsp4 | Tsp42Ep | FBtr0089062 | FBtr0089062 | 0.00217819 | 1.51394 | 1.51394 |
| 18182219 | FBtr0301800 // CG4 | CG42598 | FBtr0301800 | FBtr0301800 | 0.00222383 | 2.77869 | 2.77869 |
| 18136612 | FBtr0302224 // CG3 | CG31687 | FBtr0302224 | FBtr0302224 | 0.00222458 | 0.518634 | -1.92814 |
| 18185555 | FBtr0082225 // CG3 | CG3999 | FBtr0082225 | FBtr0082225 | 0.00226019 | 1.59768 | 1.59768 |
| 18177768 | FBtr0332644 // CG1 | CG10654 | FBtr0332644 | FBtr0332644 | 0.00226421 | 1.5219 | 1.5219 |
| 18160832 | FBtr0086883 // CG6 | CG6406 | FBtr0086883 | FBtr0086883 | 0.00233508 | 0.666222 | -1.501 |
| 18153634 | FBtr0273311 // CG3 | CG30268 | FBtr0273311 | FBtr0273311 | 0.00235174 | 0.663168 | -1.50791 |
| 18208052 | FBtr0273372 // CG1 | CG1409 | FBtr0273372 | FBtr0273372 | 0.0024166 | 1.66953 | 1.66953 |
| 18203730 | NR_074050 // CR43 | CR43846 | NR_074050 | FBtr0332548 | 0.00242194 | 1.72483 | 1.72483 |
| 18179973 | NR_048293 // CR32 | CR32194 | NR_048293 | FBtr0306733 | 0.00242889 | 0.562341 | -1.77828 |
| 18141140 | FBtr0077985 // Tfb4 | Tfb4 | FBtr0077985 | FBtr0077985 | 0.00247098 | 0.646175 | -1.54757 |
| 18217518 | FBtr0070703 // CG3 | CG32773 | FBtr0070703 | FBtr0070703 | 0.00249674 | 1.56242 | 1.56242 |
| 18145531 | NM_206014 // CheB | CheB38a | NM_206014 | FBtr0081453 | 0.00258128 | 0.635113 | -1.57452 |
| 18179847 | FBtr0076230 // CG3 | CG32073 | FBtr0076230 | FBtr0076230 | 0.00260104 | 0.645316 | -1.54963 |
| 18200430 | FBtr0085719 // CG1 | CG15545 | FBtr0085719 | FBtr0085719 | 0.00263982 | 0.611303 | -1.63585 |
| 18136549 | FBtr0078008 // Nnf1 | Nnf1b | FBtr0078008 | FBtr0078008 | 0.00268247 | 0.617569 | -1.61925 |
| 18145527 | NM_206013 // CheB | CheB38b | NM_206013 | FBtr0081454 | 0.00268851 | 1.79492 | 1.79492 |
| 18188849 | FBtr0085328 // CG1 | CG14529 | FBtr0085328 | FBtr0085328 | 0.00269343 | 1.55071 | 1.55071 |
| 18133376 | FBtr0331628 // CG1 | CG17264 | FBtr0331628 | FBtr0331628 | 0.00271668 | 1.6608 | 1.6608 |
| 18180564 | FBtr0290083 // CG3 | CG34025 | FBtr0290083 | FBtr0290083 | 0.00274958 | 2.13275 | 2.13275 |
| 18180577 | NR_073977 // CR40 | CR40053 | NR_073977 | FBtr0334764 | 0.00275568 | 1.63737 | 1.63737 |
| 18154235 | NR_001757 // snoR | snoRNA:Me28S-A1666a | NR_001757 | FBtr0086845 | 0.00276068 | 0.535142 | -1.86866 |
| 18144657 | FBtr0079324 // CG3 | CG31636 | FBtr0079324 | FBtr0079324 | 0.0027836 | 0.522806 | -1.91276 |
| 18185993 | NM_169484 // Cyp3 | Cyp304a1 | NM_169484 | FBtr0082691 | 0.00280519 | 1.75525 | 1.75525 |
| 18165923 | FBtr0089585 // TpnC | TpnC73F | FBtr0089585 | FBtr0089585 | 0.00281897 | 0.642287 | -1.55694 |
| 18137464 | FBtr0112355 // CG3 | CG34164 | FBtr0112355 | FBtr0112355 | 0.00285761 | 1.52846 | 1.52846 |
| 18154441 | FBtr0112401 // CG3 | CG34208 | FBtr0112401 | FBtr0112401 | 0.00287954 | 0.606391 | -1.6491 |
| 18135060 | NM_080363 // Gr33 | Gr33a | NM_080363 | FBtr0080337 | 0.00288815 | 2.16527 | 2.16527 |
| 18173351 | NR_048295 // CR43 | CR43280 | NR_048295 | FBtr0306721 | 0.00291573 | 1.71169 | 1.71169 |
| 18180792 | NR_003865 // snoR | snoRNA:Me18S-G962 | NR_003865 | FBtr0114353 | 0.00296148 | 0.658612 | -1.51834 |
| 18192084 | FBtr0303835 // CG4 | CG42828 | FBtr0303835 | FBtr0303835 | 0.00297047 | 0.608589 | -1.64315 |
| 18154364 | NR_003807 // snoR | snoRNA:Psi18S-1347b | NR_003807 | FBtr0113514 | 0.00298618 | 0.496267 | -2.01505 |
| 18170040 | FBtr0332755 // Cpr7 | Cpr76Bd | FBtr0332755 | FBtr0332755 | 0.0029915 | 0.645878 | -1.54828 |
| 18187281 | FBtr0083968 // CG1 | CG15696 | FBtr0083968 | FBtr0083968 | 0.00312586 | 1.53429 | 1.53429 |
| 18177346 | FBtr0331581 // CG6 | CG6685 | FBtr0331581 | FBtr0331581 | 0.0034324 | 1.57176 | 1.57176 |
| 18152089 | FBtr0071720 // CG9 | CG9294 | FBtr0071720 | FBtr0071720 | 0.00346974 | 0.624011 | -1.60254 |
| 18165426 | FBtr0303474 // Hsp6 | Hsp67Bb | FBtr0303474 | FBtr0303474 | 0.00347358 | 1.86333 | 1.86333 |
| 18204225 | NR_003113 // sphin | sphinx | NR_003113 | FBtr0111045 | 0.00354107 | 0.61601 | -1.62335 |
| 18205233 | FBtr0302236 // CG4 | CG42619 | FBtr0302236 | FBtr0302236 | 0.00354416 | 1.5165 | 1.5165 |
| 18157906 | NM_079097 // Or59 | Or59a | NM_079097 | FBtr0072023 | 0.00354684 | 2.24543 | 2.24543 |
| 18181917 | NM_001274925 // C | CG43678 | NM_001274925 | FBtr0310458 | 0.00355516 | 1.56894 | 1.56894 |
| 18196834 | FBtr0082504 // CG3 | CG3397 | FBtr0082504 | FBtr0082504 | 0.00362701 | 0.628547 | -1.59097 |
| 18180764 | NR_003840 // snoR | snoRNA:Me28S-C788b | NR_003840 | FBtr0114338 | 0.00365231 | 0.514899 | -1.94213 |
| 18134570 | FBtr0079867 // CG4 | CG4017 | FBtr0079867 | FBtr0079867 | 0.00368303 | 1.67121 | 1.67121 |
| 18138490 | NR_047959 // CR43 | CR43357 | NR_047959 | FBtr0307107 | 0.00368886 | 0.599821 | -1.66717 |
| 18199408 | FBtr0084729 // CG1 | CG13641 | FBtr0084729 | FBtr0084729 | 0.00381788 | 1.533 | 1.533 |
| 18192497 | FBtr0309178 // CG4 | CG43447 | FBtr0309178 | FBtr0309178 | 0.00392117 | 1.70589 | 1.70589 |
| 18203842 | FBtr0302249 // CG4 | CG42621 | FBtr0302249 | FBtr0302249 | 0.00392801 | 1.69162 | 1.69162 |
| 18138259 | NR_047913 // mir-9 | mir-932 | NR_047913 | FBtr0304243 | 0.00399455 | 1.74634 | 1.74634 |
| 18209064 | FBtr0074033 // CG6 | CG6299 | FBtr0074033 | FBtr0074033 | 0.00400532 | 1.54695 | 1.54695 |
| 18138427 | FBtr0306282 // CG4 | CG43230 | FBtr0306282 | FBtr0306282 | 0.00400594 | 1.51881 | 1.51881 |
| 18203810 | FBtr0302251 // CG4 | CG40155 | FBtr0302251 | FBtr0302251 | 0.00401007 | 1.50948 | 1.50948 |
| 18176596 | FBtr0077067 // Jon6 | Jon65Ai | FBtr0077067 | FBtr0077067 | 0.00402501 | 1.81143 | 1.81143 |
| 18144309 | FBtr0308305 // lecti | lectin-21Cb | FBtr0308305 | FBtr0308305 | 0.00421741 | 1.65001 | 1.65001 |
| 18215350 | FBtr0332298 // CG1 | CG11816 | FBtr0332298 | FBtr0332298 | 0.00422015 | 1.70947 | 1.70947 |
| 18211710 | NR_047840 // mir-9 | mir-974 | NR_047840 | FBtr0304268 | 0.00425713 | 1.55742 | 1.55742 |
| 18142330 | FBtr0302518 // CG1 | CG13793 | FBtr0302518 | FBtr0302518 | 0.00441986 | 0.580834 | -1.72166 |
| 18156058 | NR_073876 // CR43 | CR43811 | NR_073876 | FBtr0332254 | 0.00442297 | 0.581472 | -1.71977 |
| 18179568 | FBtr0290214 // CG1 | CG15024 | FBtr0290214 | FBtr0290214 | 0.00446812 | 2.36175 | 2.36175 |
| 18171553 | FBtr0076806 // CG3 | CG32373 | FBtr0076806 | FBtr0076806 | 0.00459528 | 1.56626 | 1.56626 |
| 18167307 | FBtr0072871 // CG8 | CG8960 | FBtr0072871 | FBtr0072871 | 0.00462695 | 0.453254 | -2.20627 |
| 18140556 | NM_078883 // Ugt3 | Ugt37a1 | NM_078883 | FBtr0081372 | 0.00466029 | 1.81101 | 1.81101 |
| 18185335 | FBtr0082016 // HP1e | HP1e | FBtr0082016 | FBtr0082016 | 0.0046954 | 0.629014 | -1.58979 |
| 18159442 | NM_136730 // Obp4 | Obp46a | NM_136730 | FBtr0088359 | 0.00495845 | 0.604753 | -1.65357 |
| 18211732 | NR_047813 // mir-1 | mir-12 | NR_047813 | FBtr0304288 | 0.00526512 | 1.60827 | 1.60827 |
| 18218636 | NR_047858 // mir-1 | mir-1007 | NR_047858 | FBtr0304483 | 0.00535768 | 0.620963 | -1.6104 |
| 18132226 | NM_080269 // Ugt3 | Ugt37b1 | NM_080269 | FBtr0079242 | 0.00539615 | 1.66818 | 1.66818 |
| 18192598 | NR_074011 // CR43 | CR43642 | NR_074011 | FBtr0310043 | 0.00548812 | 1.68724 | 1.68724 |
| 18208910 | FBtr0073818 // CG1 | CG11134 | FBtr0073818 | FBtr0073818 | 0.00554451 | 1.5128 | 1.5128 |
| 18133338 | FBtr0077722 // CG2 | CG2964 | FBtr0077722 | FBtr0077722 | 0.00557427 | 0.637905 | -1.56763 |
| 18173568 | NR_073909 // CR43 | CR43701 | NR_073909 | FBtr0321280 | 0.00563549 | 0.553547 | -1.80653 |
| 18154229 | NR_001764 // snoR | snoRNA:snR38:54Eb | NR_001764 | FBtr0086852 | 0.00564236 | 0.443735 | -2.2536 |
| 18160841 | FBtr0089560 // CG6 | CG6385 | FBtr0089560 | FBtr0089560 | 0.00564393 | 0.641331 | -1.55926 |
| 18198503 | FBtr0083935 // CG4 | CG4000 | FBtr0083935 | FBtr0083935 | 0.00567185 | 0.653727 | -1.52969 |
| 18138665 | NR_073681 // CR43 | CR43717 | NR_073681 | FBtr0329833 | 0.0058284 | 0.604295 | -1.65482 |
| 18200651 | FBtr0308027 // Ugt8 | Ugt86Dg | FBtr0308027 | FBtr0308027 | 0.00584083 | 1.8053 | 1.8053 |
| 18210822 | NM_167237 // Rab9 | Rab9D | NM_167237 | FBtr0071490 | 0.00597518 | 0.544011 | -1.8382 |
| 18201947 | FBtr0304612 // CG3 | CG33337 | FBtr0304612 | FBtr0304612 | 0.0060589 | 0.623573 | -1.60366 |
| 18146030 | NM_136296 // Ir40a | Ir40a | NM_136296 | FBtr0336971 | 0.00608937 | 0.641754 | -1.55823 |
| 18197802 | FBtr0083376 // CG3 | CG3678 | FBtr0083376 | FBtr0083376 | 0.00612022 | 1.9156 | 1.9156 |
| 18154142 | NM_001014533 // V | Vkor | NM_001014533 | FBtr0091510 | 0.0061325 | 1.87057 | 1.87057 |
| 18193664 | FBtr0083502 // Edg9 | Edg91 | FBtr0083502 | FBtr0083502 | 0.00620917 | 1.88002 | 1.88002 |
| 18203806 | FBtr0302250 // CG1 | CG15831 | FBtr0302250 | FBtr0302250 | 0.00635958 | 1.73598 | 1.73598 |
| 18189740 | FBtr0332552 // mthl | mthl11 | FBtr0332552 | FBtr0332552 | 0.0064113 | 0.609793 | -1.6399 |
| 18171214 | FBtr0075953 // CG3 | CG32105 | FBtr0075953 | FBtr0075953 | 0.00642546 | 1.58271 | 1.58271 |
| 18211864 | NR_047780 // CR43 | CR43297 | NR_047780 | FBtr0306827 | 0.00657176 | 0.628422 | -1.59129 |
| 18180415 | FBtr0302886 // CG3 | CG33062 | FBtr0302886 | FBtr0302886 | 0.00669126 | 0.604246 | -1.65495 |
| 18138589 | NR_047931 // mir-4 | mir-4970 | NR_047931 | FBtr0309610 | 0.0066951 | 1.5027 | 1.5027 |
| 18132155 | FBtr0112925 // Traf4 | Traf4 | FBtr0112925 | FBtr0112925 | 0.00670887 | 0.611355 | -1.63571 |
| 18144526 | FBtr0081310 // TotF | TotF | FBtr0081310 | FBtr0081310 | 0.00674128 | 1.60876 | 1.60876 |
| 18192354 | FBtr0306929 // CG4 | CG43335 | FBtr0306929 | FBtr0306929 | 0.00687069 | 0.487981 | -2.04926 |
| 18188592 | FBtr0085103 // CG1 | CG14258 | FBtr0085103 | FBtr0085103 | 0.00705149 | 0.622601 | -1.60617 |
| 18182313 | FBtr0085614 // CecA | CecA2 | FBtr0085614 | FBtr0085614 | 0.00721852 | 0.543909 | -1.83854 |
| 18155896 | NR_048045 // snoR | snoRNA:gp210-a | NR_048045 | FBtr0309778 | 0.00730145 | 1.51944 | 1.51944 |
| 18162416 | NM_079084 // Gr58 | Gr58b | NM_079084 | FBtr0071732 | 0.00731806 | 1.64396 | 1.64396 |
| 18162874 | FBtr0071697 // CG3 | CG30289 | FBtr0071697 | FBtr0071697 | 0.00780402 | 1.53309 | 1.53309 |
| 18137364 | NR_003778 // snoR | snoRNA:Psi18S-525i | NR_003778 | FBtr0113553 | 0.00788397 | 1.73283 | 1.73283 |
| 18195297 | FBtr0083938 // KaiRI | KaiRIA | FBtr0083938 | FBtr0083938 | 0.00789781 | 1.55423 | 1.55423 |
| 18190618 | FBtr0084613 // CG3 | CG33341 | FBtr0084613 | FBtr0084613 | 0.0080011 | 1.50957 | 1.50957 |
| 18186466 | FBtr0083089 // CG5 | CG5038 | FBtr0083089 | FBtr0083089 | 0.00804169 | 1.54699 | 1.54699 |
| 18161473 | FBtr0071616 // CG9 | CG9752 | FBtr0071616 | FBtr0071616 | 0.00812308 | 0.604383 | -1.65458 |
| 18140456 | FBtr0330647 // CG1 | CG11592 | FBtr0330647 | FBtr0330647 | 0.00813524 | 0.650022 | -1.53841 |

**Supplementary Table S3:** Genes altered by endurance training in exercised *Ra* flies. 4442 genes were identified, with transcripts upregulated by exercise indicated by (+) fold-change and transcripts downregulated by exercise with (-).
